# Chemical Screening Identifies Enhancers of Mutant Oligodendrocyte Survival and Unmasks a Distinct Pathological Phase in Pelizaeus-Merzbacher Disease

**DOI:** 10.1016/j.stemcr.2018.07.015

**Published:** 2018-08-23

**Authors:** Matthew S. Elitt, H. Elizabeth Shick, Mayur Madhavan, Kevin C. Allan, Benjamin L.L. Clayton, Chen Weng, Tyler E. Miller, Daniel C. Factor, Lilianne Barbar, Baraa S. Nawash, Zachary S. Nevin, Angela M. Lager, Yan Li, Fulai Jin, Drew J. Adams, Paul J. Tesar

**Affiliations:** 1 Department of Genetics and Genome Sciences, Case Western Reserve University School of Medicine, Cleveland, OH 44106, USA; 2 Department of Neurosciences, Case Western Reserve University School of Medicine, Cleveland, OH 44106, USA; 3 Department of Population and Quantitative Health Sciences, Case Western Reserve University School of Medicine, Cleveland, OH 44106, USA; 4 Department of Engineering and Computer Science, Case Western Reserve University School of Medicine, Cleveland, OH 44106, USA; 5 Case Comprehensive Cancer Center, Case Western Reserve University School of Medicine, Cleveland, OH 44106, USA

**Keywords:** iPSC disease modeling, high-throughput screening, oligodendrocyte progenitor cells, oligodendrocytes, myelin, Pelizaeus-Merzbacher disease, proteolipid protein 1, endoplasmic reticulum stress, PLP1, rare disease

## Abstract

Pelizaeus-Merzbacher disease (PMD) is a fatal X-linked disorder caused by loss of myelinating oligodendrocytes and consequent hypomyelination. The underlying cellular and molecular dysfunctions are not fully defined, but therapeutic enhancement of oligodendrocyte survival could restore functional myelination in patients. Here we generated pure, scalable quantities of induced pluripotent stem cell-derived oligodendrocyte progenitor cells (OPCs) from a severe mouse model of PMD, *Plp1*^*jimpy*^. Temporal phenotypic and transcriptomic studies defined an early pathological window characterized by endoplasmic reticulum (ER) stress and cell death as OPCs exit their progenitor state. High-throughput phenotypic screening identified a compound, Ro 25–6981, which modulates the ER stress response and rescues mutant oligodendrocyte survival in *jimpy*, *in vitro* and *in vivo*, and in human PMD oligocortical spheroids. Surprisingly, increasing oligodendrocyte survival did not restore subsequent myelination, revealing a second pathological phase. Collectively, our work shows that PMD oligodendrocyte loss can be rescued pharmacologically and defines a need for multifactorial intervention to restore myelination.

## Highlights

• Oligodendrocyte differentiation triggers ER stress and early cell loss in *jimpy*
• HTS reveals Ro 25–6981, an enhancer of *jimpy* oligodendrocytes *in vitro* and *in vivo*
• Ro 25–6981 increases human mutant oligodendrocytes in 3D cortical spheroids
• Rescued oligodendrocytes fail to myelinate, revealing a second phase of pathology

## Teaser

Tesar and colleagues interrogate the severe genetic myelin disorder Pelizaeus-Merzbacher disease (PMD) using iPSC-derived oligodendrocyte progenitor cells (OPCs). Using high-throughput phenotypic screening, they rescue cell death of mutant oligodendrocytes immediately after initiation of differentiation with chemical modulators, including Ro 25–6981. Interestingly oligodendrocyte restoration did not lead to widespread myelination, unmasking a new phase of the disease.

## Introduction

Leukodystophies are a class of rare, heritable disorders characterized by a loss of central nervous system (CNS) myelin. Collectively these diseases impact approximately 1 in 7,500 live births, and are typified by extensive neurological impairment with reduced life expectancy (Bonkowsky et al., 2010, Parikh et al., 2015). While the causative mutations for these disorders are well established, a comprehensive understanding of the molecular and cellular phenotypes of each mutation remains elusive. Genotype-specific, molecular, and cellular phenotyping of oligodendrocytes—the myelinating glia of the CNS—during key pathological events would be invaluable for filling this deficiency and underpin future precision medicine initiatives.

We sought to establish the technology to systemically interrogate the archetypal, cell-autonomous leukodystrophy called Pelizaeus-Merzbacher disease (PMD) (MIM no. 312080) (Garbern, 2007, van der Knaap and Bugiani, 2017). PMD is a fatal X-linked disorder of the CNS caused by mutations in *proteolipid protein 1* (*PLP1*). PLP and its splice isoform DM20 are almost exclusively expressed in oligodendrocytes and oligodendrocyte progenitor cells (OPCs), respectively (Mallon et al., 2002). While the disease is thought to be mediated through mutant PLP-induced endoplasmic reticulum (ER) stress (Garbern, 2007), complete appreciation of the cellular and molecular pathology in PMD remains elusive, in part due to complexities in accessing or appropriately modeling the disease-affected oligodendrocyte lineage during critical developmental windows (Bradl et al., 1999, Cerghet et al., 2001, Duncan et al., 2011, Gow et al., 1998, Knapp et al., 1986, Knapp et al., 1987, Southwood et al., 2002, Williams and Gard, 1997). Our group and others has recently begun to address these tractability issues using human PMD patient, induced pluripotent stem cell (iPSC)-based approaches (Nevin et al., 2017, Numasawa-Kuroiwa et al., 2014), but these cellular models are still constrained by multi-month differentiations and are confounded by contaminating cell types.

Our mouse pluripotent stem cell-based technology enables the rapid generation of high-purity, expandable OPCs that are capable of coordinated differentiation to oligodendrocytes *in vitro* over a defined 3-day period (Najm et al., 2011). This system provides precise developmental control to examine the oligodendrocyte lineage without artifact from contaminating cell types (Najm et al., 2011, Najm et al., 2015). Notably, these tools and similiar techniques have recently been coupled to high-throughput compound screening investigations and have identified novel therapeutics for autoimmune-mediated myelin disorders (Green et al., 2017, Helman et al., 2015, Hubler et al., 2018, Lee et al., 2013, Najm et al., 2015).

Here we extend this cellular technology to a well-established animal model for the severe, connatal form of PMD called the “*jimpy*” (*Plp1*^*jimpy*^) mouse (Duncan et al., 2011, Phillips, 1954, Yool et al., 2000), providing scalable access to the previously restricted PMD oligodendrocyte lineage. Using iPSC-derived *jimpy* OPCs, we first establish critical cellular and molecular deficits at key developmental stages during oligodendrocyte differentiation. We overcome this pathology using high-throughput chemical screening, identifying compounds capable of rescuing mutant oligodendrocyte survival in *jimpy* and human PMD cultures *in vitro*, and in *jimpy* mice *in vivo*. Surprisingly, these studies revealed that enhancing mutant oligodendrocyte survival does not, on its own, confer an expected restoration of myelin, unmasking a second stage of pathology and informing future phenotyping and therapeutic strategies for PMD.

## Results

### *Jimpy* iPSC-Derived OPCs Reveal Early Differentiation Pathology

We established three independent iPSC lines from *jimpy* and isogenic wild-type littermate mice (see Experimental Procedures). iPSC lines exhibited typical iPSC morphology and expressed the canonical pluripotency markers Oct3/4 and Nanog (Figures S1A–S1D). Sanger sequencing confirmed *jimpy* mutation status (Figure S1E) and all iPSC lines displayed grossly normal karyotypes (Figure S1F). We directed all six iPSC lines to form high-purity OPCs (see Experimental Procedures) expressing the canonical OPC transcription factors Sox10 and Olig2 (Figures S2A–S2E), which had no overt morphological differences (Figure S2F).

**Figure 1:**
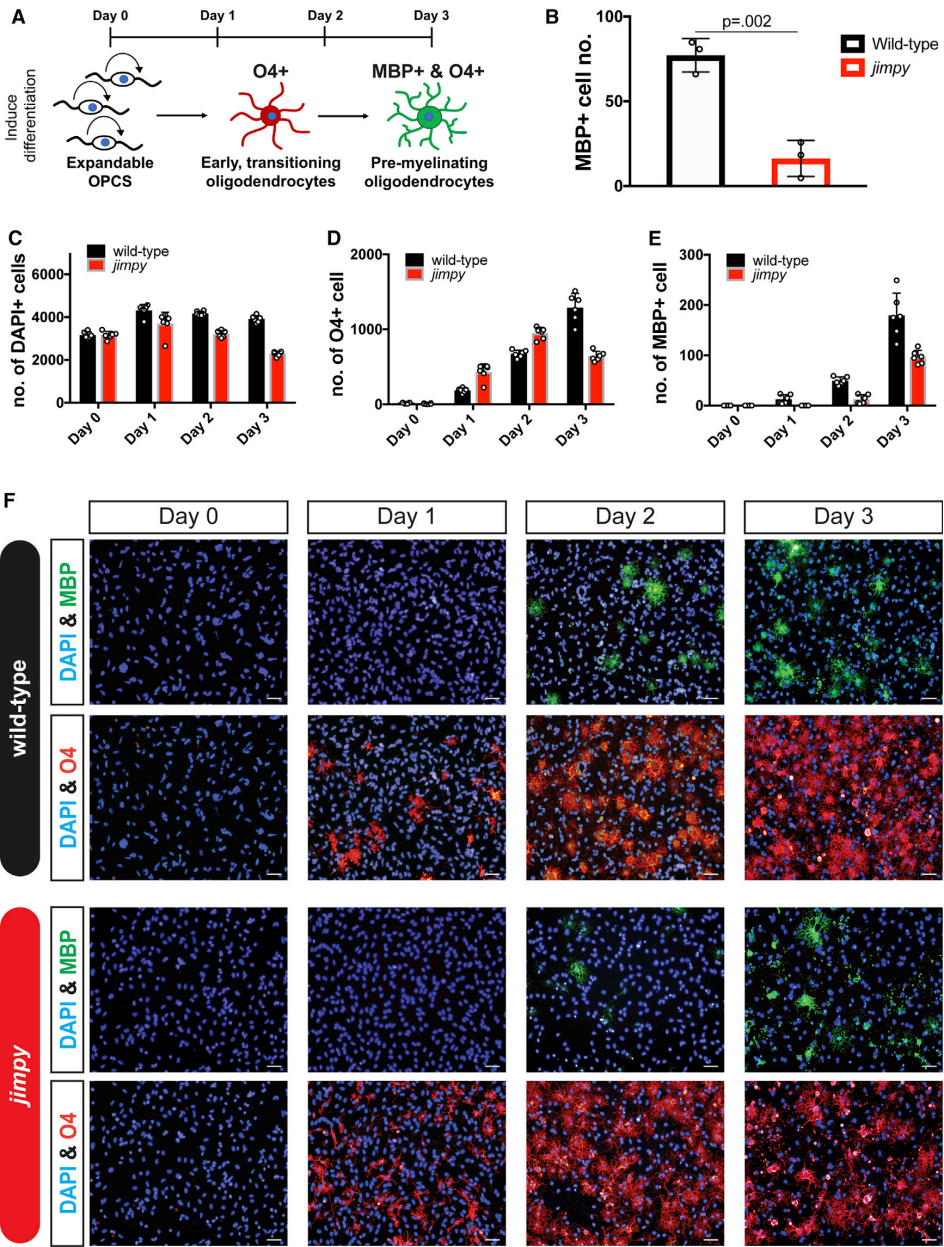
Temporal Dissection of Cellular Phenotypes Reveals Cell Death Just as *Jimpy* OPCs Commit to an Oligodendrocyte Fate (A) Schematic summary of the experimental assay. (B) Quantification of wild-type and *jimpy* MBP+ oligodendrocytes after oligodendrocyte differentiation. n = 3 distinct cell lines per genotype, with each replicate value (comprising a mean of n = 4 replicate wells per cell line) represented by a white circle. p values calculated using a two-way, unpaired t test between genotypes. (C–E) Wild-type and *jimpy* oligodendrocyte differentiation time-course data showing quantification of total (C) DAPI+ cells, (D) O4+ cells, and (E) MBP+ oligodendrocytes. n = 6 replicate wells per genotype, with each replicate value represented by a white circle. (F) Representative immunocytochemistry images from time course showing MBP+ oligodendrocytes (green), O4+ cells (red), and total cells (DAPI, blue). Scale bars, 50 μm. Error bars represent mean ± SD. See also Figures S1 and S2, and Video S1.

Previous reports have documented cellular phenotypes at discrete stages during oligodendrogenesis in PMD cellular and animal models (Gow et al., 1998, Nevin et al., 2017, Numasawa-Kuroiwa et al., 2014, Readhead et al., 1994). We directed all iPSC-derived *jimpy* and wild-type control OPC lines toward an oligodendrocyte fate (Figure 1A) and noted a striking absence of MBP+ oligodendrocytes in all *jimpy* lines (Figure 1B). We further parsed this cellular defect with time-course immunocytochemistry. As expected, *jimpy* MBP+ oligodendrocytes failed to accumulate across all time points (Figures 1E and 1F). Surprisingly we noted substantial cell death well before MBP+ oligodendrocyte formation, within 1 day after inducing differentiation (Figures 1C and 1F), which we further confirmed by time-lapse imaging (Video S1). Intriguingly, *jimpy* OPCs showed increased propensity to acquire the late OPC/immature oligodendrocyte marker O4 early in the differentiation process (Figures 1D and 1F), which may reflect a feedback loop to promote stem cell exit in the context of severe cell loss during oligodendrocyte differentiation. Together these results precisely define an early developmental susceptibility period during which transitioning *jimpy* OPCs die as they exit their progenitor cell state.

Video S1. Time-Lapse of Oligodendrocyte Differentiation, Related to Figure 1

### Transcriptome Profiling Reveals ER Stress in *Jimpy* Oligodendrocytes Immediately after the Onset of OPC Differentiation

**Figure 2:**
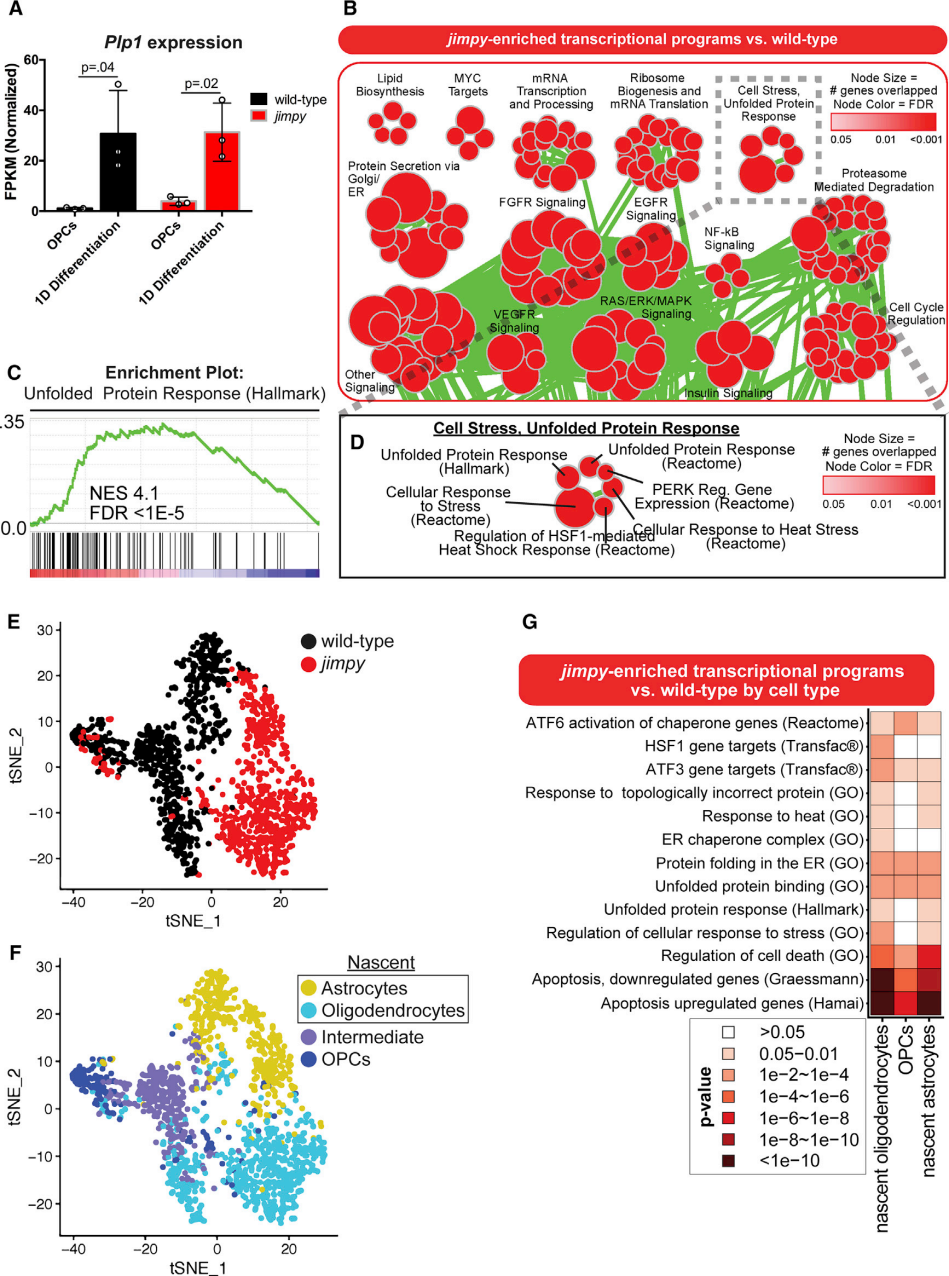
RNA-Seq and scRNA-Seq Reveal Transcriptome Signatures of ER Stress and Cell Death in Early, Differentiating *Jimpy* Oligodendrocytes (A) *Plp1* mRNA expression in wild-type and *jimpy* OPCs and in cultures during oligodendrocyte (at day 1). Error bars represent mean ± SD. n = 3 sequenced cell lines per genotype, with each replicate represented by a white circle. p values calculated using a two-way, unpaired t test for each genotype. (B) Gene ontology enrichment map for RNA-seq data showing enriched pathways in *jimpy* after 1 day of oligodendrocyte differentiation. Node size indicates the number genes represented in the pathway. Color intensity indicates false discovery rate (FDR). Node overlap indicates shared pathway genes. n = 3 cell lines, per genotype. (C) *Jimpy* enrichment plots for the hallmark unfolded protein response pathway genes relative to wild-type. The upper plot (green line) details the running sum statistic of the ranked list of genes with the normalized enrichment score (NES) and FDR. The lower plot displays a gene's position in the ranked list of genes for the pathway. (D) Enlargement of the “cell stress, unfolded protein response” node group. (E and F) tSNE plots showing (E) genotype and (F) cell-type distributions. n = 1 cell line per genotype. (G) Heatmap showing pathway enrichment in *jimpy* cell types relative to their wild-type counterparts. Scale corresponds to p value. See also Figure S3, Tables S1, S2, and S3.

We analyzed transcriptomic changes between *jimpy* and wild-type cultures after 1 day of oligodendrocyte differentiation by gene set enrichment analysis (GSEA) (Table S1), at the onset of *jimpy* cell death and upregulation of the disease-causative gene, *Plp1* (Figure 2A). *Jimpy* cultures showed multiple dysregulated pathways comprising several gene sets linked to the ER stress response (Figure 2B; Table S1), including the unfolded protein response (UPR) pathway (Figures 2C and 2D).

We next sought to precisely define the cell types exhibiting these pathological stress signatures using single-cell RNA sequencing (scRNA-seq). Unsupervised clustering separated *jimpy* and wild-type cells in t-distributed stochastic neighbor embedding (tSNE) space (Figure 2E), while also arranging OPCs, nascent oligodendrocytes, nascent astrocytes, and transitioning cells (between OPC and nascent oligodendrocyte) into distinct populations defined by marker expression (Figures 2F and S3B–S3G). We then generated a list of differentially expressed genes between genotypes for each cell type present in both cultures (Table S2). GSEA on the nascent oligodendrocyte populations revealed a significant enrichment of ER stress and cell death pathways in this early *jimpy* oligodendrocyte population (Figures 2G; Table S3), confirming the presence of canonical PMD molecular pathology in the disease-affected cell type.

Interestingly we also noted enrichment for similar pathways in nascent *jimpy* astrocytes, indicating possible astrocyte contribution to disease in PMD (Skoff, 1976). However these transcriptional changes could simply represent a response to the substantial cell death in the cultures. To test this, we first directed iPSC-derived *jimpy* and wild-type OPCs toward an astrocyte fate. We observed no difference in the percentage of GFAP+ astrocytes in wild-type and *jimpy* cultures (Figures S3H–S3J). Next we performed astrocyte co-culture experiments to examine the effect of *jimpy* and wild-type astrocytes on oligodendrocyte survival. This analysis revealed no astrocyte-mediated effect on mutant or wild-type oligodendrocytes (Figures S3K–S3P). Together these data demonstrate that astrocytes are not the primary mediators of oligodendrocyte death in *jimpy* cultures and that pathological changes in *jimpy* astrocytes are likely secondary to primary oligodendrocyte death.

### High-Throughput Screening Reveals Chemical Enhancers of *Jimpy* Oligodendrocyte Survival

**Figure 3:**
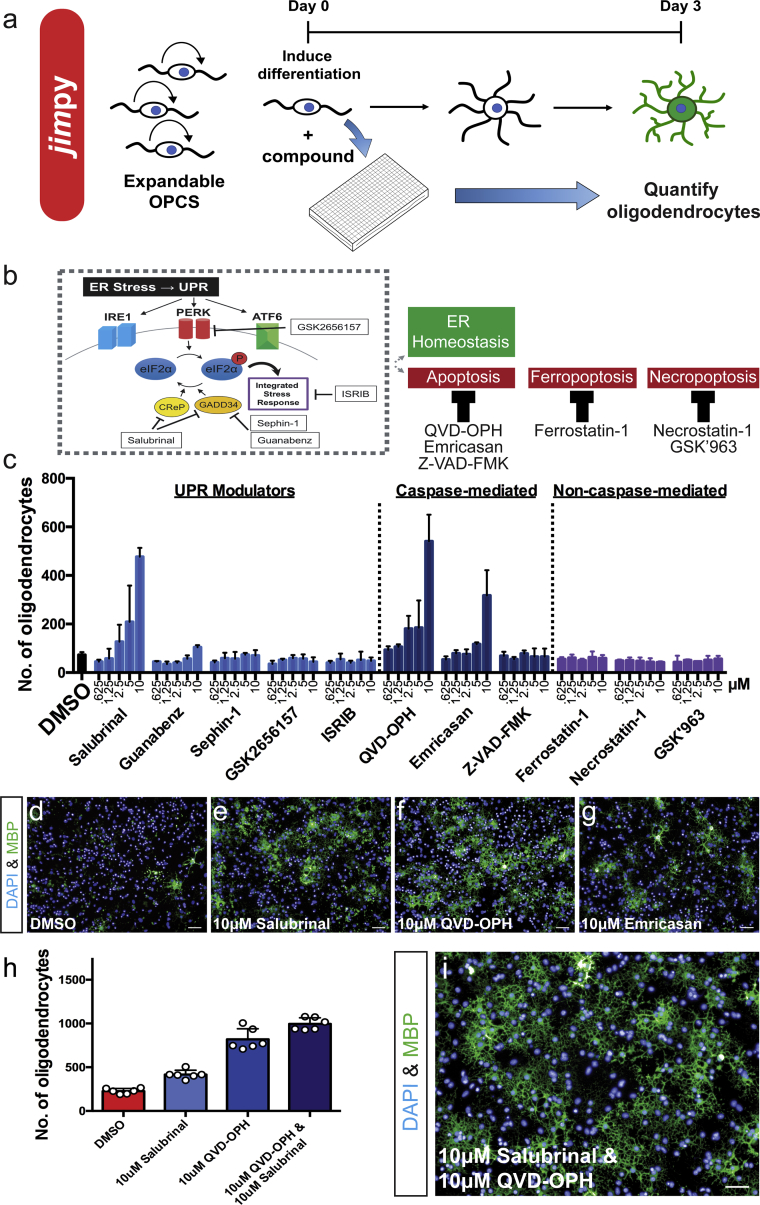
High-Throughput Chemical Screening Identifies Cellular and Molecular Modulators of *Jimpy* Pathology (A) Chemical screening schematic. (B) Chemical-targeting of ER stress-related sequelae by UPR modulation or inhibition of caspase-mediated apoptosis. (C) Quantification of MBP+ oligodendrocytes per compound dose (five doses, 10 μM to 625 nM) after 3-day oligodendrocyte differentiation of iPSC-derived *jimpy* OPCs. n = 3 replicate wells per dose, per compound. (D–G) Representative immunocytochemistry images after oligodendrocyte differentiation of *jimpy* OPCs treated with (D) DMSO vehicle, (E) 10 μM salubrinal, (F) 10 μM Q-VD-OPh, and (G) 10 μM emricasan, showing MBP+ oligodendrocytes (green) and total DAPI+ cells (blue). (H) Quantification of MBP+ *jimpy* oligodendrocytes for DMSO vehicle (red), 10 μM salubrinal (light blue), 10 μM Q-VD-OPh (blue), and 10 μM salubrinal and 10 μM Q-VD-OPh (dark blue). n = 6 replicate wells per treatment, with each replicate value represented by a white circle. (I) Representative immunocytochemistry images after oligodendrocyte differentiation of *jimpy* OPCs treated with 10 μM salubrinal and 10 μM Q-VD-OPh, showing MBP+ oligodendrocytes (green) and total DAPI+ cells (blue). Error bars represent mean ± SD. Scale bars, 50 μm. See also Figure S4.

Our stem cell platform provides scalable access to a defined developmental window for potential therapeutic intervention in *jimpy*. To explore whether small molecules could be used to resolve the molecular and cellular pathology in early differentiating *jimpy* OPCs, we developed a chemical screening pipeline to identify compounds that improved the survival of MBP+ *jimpy* oligodendrocytes in conditions promoting oligodendrocyte differentiation (Figure 3A). We first performed a hypothesis-driven compound screen to interrogate the protein kinase R-like ER kinase (PERK) arm of the UPR, as well as a variety of cell death pathways that could drive oligodendrocyte loss during unresolved ER stress (Figure 3B) (Nevin et al., 2017). Using *jimpy* iPSC-derived OPCs we applied each compound in triplicate over a 5-point dose curve (10 μM to 625 nM) with simultaneous initiation of oligodendrocyte differentiation with thyroid hormone and then quantified MBP+ oligodendrocyte number after 3 days. Out of five compounds that modulate distinct targets in the PERK pathway, only salubrinal (Figures 3C–3E), a dual GADD34 and CReP inhibitor (Boyce et al., 2005), showed a dose-dependent ability to enhance *jimpy* oligodendrocyte survival. Examination of cell death-modulating compounds identified the high-efficacy pan-caspase inhibitors, emricasan and Q-VD-OPh, as effective mediators of *jimpy* oligodendrocyte death (Figures 3C, 3D, 3F, and 3G). Strikingly, the combination of Q-VD-OPh and salubrinal demonstrated an additive ability to restore *jimpy* cell number back to wild-type levels and enhance *jimpy* oligodendrocyte survival (Figures 3H, 3I, and S4). Collectively, these data show that loss of *jimpy* oligodendrocytes can be prevented by modulating ER stress and caspase-mediated cell death during the immediate transition from OPC to oligodendrocyte.

**Figure 4:**
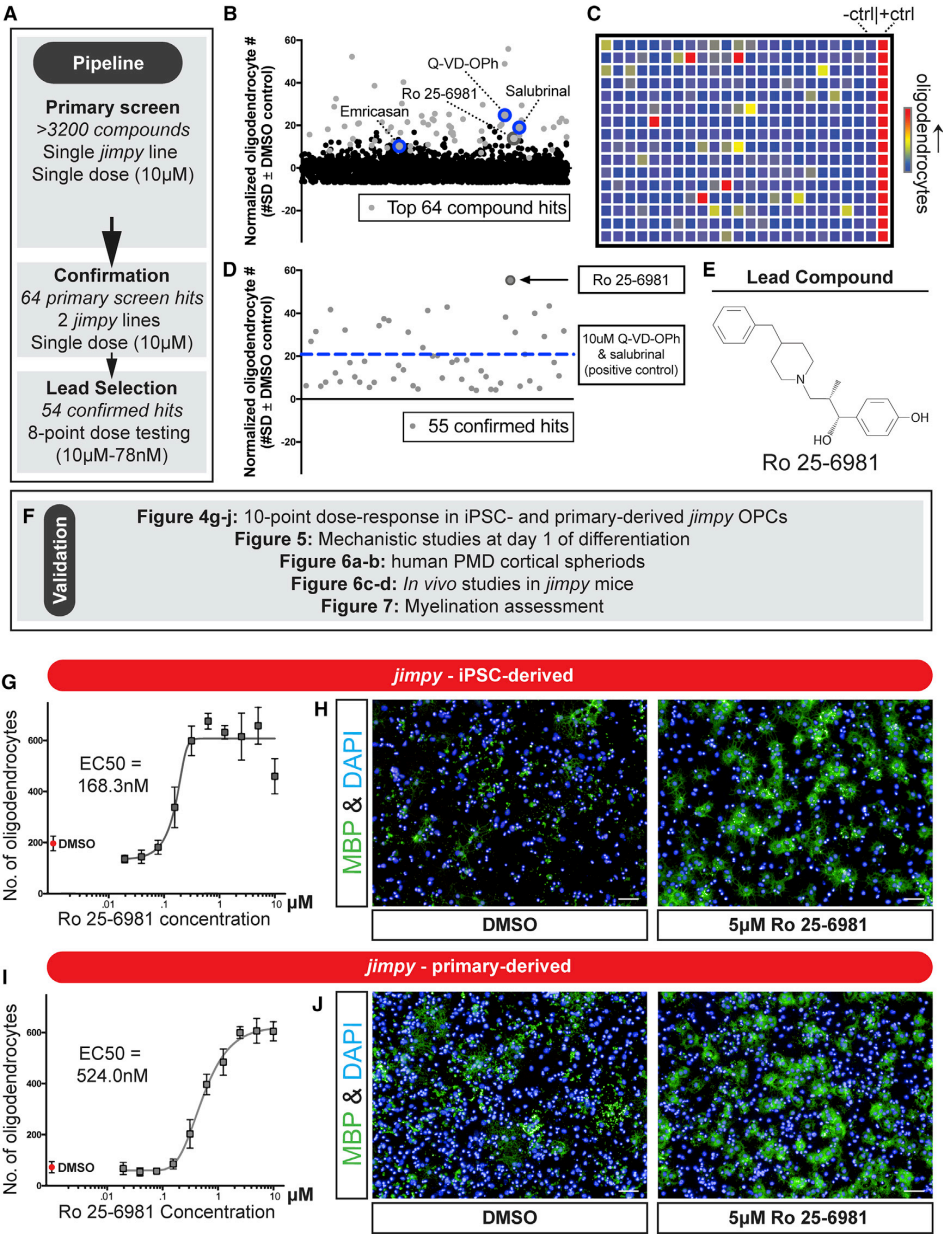
High-Throughput Chemical Screening Identifies Cellular and Molecular Modulators of *Jimpy* Pathology (A) Overview of screening pipeline with filtering steps for lead compound selection. (B) Plot of primary screen results showing top hit (gray circles) and non-hit (black circles) compounds listed by alphabetical order with their corresponding SD divergence from DMSO vehicle on the MBP+ *jimpy* oligodendrocyte metric after 3-day oligodendrocyte differentiation. n = 1 well per tested compound. (C) Heatmap of a representative primary screen plate depicting high (red) or low (blue) quantified MBP+ *jimpy* oligodendrocytes for each compound, as well as negative and positive controls. (D) Plot of confirmed hits (gray circles) listed in alphabetical order showing their maximal SD divergence from DMSO vehicle for MBP+ *jimpy* oligodendrocytes in an 8-point (10 μM to 78 nM) dose response. n = 1 well per compound dose. n = 16 replicate wells for the positive and negative control samples. (E) Chemical structure of lead compound Ro 25–6981. (F) Overview of validation pipeline for lead compound Ro 25–6981. (G and I) Quantification of MBP+ *jimpy* oligodendrocytes after differentiation of (G) iPSC-derived and (I) cerebral cortical-derived OPCs, treated with Ro 25–6981 (10-point dose response) or DMSO vehicle. Individual points represent mean ± SD. n = 3 replicate wells for each dose, n = 16 replicate wells for the DMSO vehicle control. (H and J) Representative immunocytochemistry images after oligodendrocyte differentiation of (H) iPSC-derived or (J) cerebral cortical-derived OPCs treated with DMSO vehicle or 5 μM Ro 25–6981 showing MBP+ oligodendrocytes (green) and total DAPI+ cells (blue). Scale bars, 50 μm. See also Figure S5, Tables S4, and S5.

We scaled this screening pipeline to an unbiased collection of >3,000 bioactive compounds to identify additional nodes of intervention for *jimpy* pathology. To gauge screening performance we included the combination of salubrinal plus Q-VD-OPh as a positive control and a DMSO vehicle negative control. We performed a high-throughput primary screen at a single dose of 10 μM followed by rigorous secondary screening to filter to a lead compound (Figure 4A). We evaluated the performance of our primary screen based on the dynamic range of quantified oligodendrocytes between our positive and negative controls using the *Z* factor (*Z’* = 0.566 ± 0.23 [mean ± SD]) (Figures S5A–S5C), indicating a high quality and reproducible screening assay based on established performance analytics (Zhang et al., 1999). Automated image analysis scored each well based on enhancement of MBP+ oligodendrocyte number on a per-plate basis (Figure 4C). As an orthogonal filter we also used total cell number as an overall marker of increased cell survival. All compounds were ranked (Table S4) and our top 64 hits (Figure 4B) were defined as being at least four SDs above the negative control for oligodendrocyte number and two SDs above negative control for total cell number. Of note, three of the top compound hits included Q-VD-OPh, salubrinal, and emricasan (Figure 4B), which were identified and validated in our previous hypothesis-driven chemical screen.

Next we used a stringent secondary screening pipeline to prioritize these hits to a single lead compound (Figure 4A). First, all hits were tested in OPCs generated from two additional independent *jimpy* iPSC lines using the same primary screen dose of 10 μM and primary screen hit selection criteria. The efficacy of 55 out of 64 hits was confirmed in at least one additional *jimpy* cell line (Table S5). Next, each confirmed compound was examined over an 8-point dose response (from 10 μM to 78 nM), and compounds were ranked by their potency to enhance oligodendrocyte survival (Figure 4D; Table S5). This analysis highlighted the lead compound Ro 25–6981 (Figures 4D and 4E; Table S5), which showed efficacy far superior even to the combination of salubrinal and Q-VD-OPh (Figure 4D), our screening positive control.

Next we initiated a rigorous validation procedure to comprehensively define Ro 25–6981's molecular and cellular effects in multiple, orthogonal contexts (Figure 4F). We first sought to assess the potency of Ro 25–5981 using a 10-point dose response (from 10 μM to 20 nM). This analysis revealed strong efficacy extending to nanomolar doses (half maximal effective concentration [EC_50_] = 168.3 nM) (Figures 4G and 4H). To rule out possible iPSC-derived OPC artifacts we demonstrated that Ro 25–6981 had remarkably similar dose-dependent efficacy and potency to enhance oligodendrocyte survival in primary cultures of OPCs isolated from postnatal *jimpy* mice (EC_50_ = 524.0 nM) (Figures 4I and 4J).

To examine if the cellular or molecular effects of Ro 25–6981 were specific to mutant cells we first assessed oligodendrocyte enhancement using Ro 25–5981 in a 10-point dose response (from 10 μM to 20 nM) or DMSO vehicle on cultures of differentiating wild-type and *jimpy* OPCs. In addition we also examined the effects of our *jimpy* cellular pathology positive control compounds, salubrinal and Q-VD-OPh. As expected all compounds robustly (∼3- to 5-fold) enhanced *jimpy* oligodendrocytes (Figure S6B). In contrast these same compounds showed only a modest (∼1.5- to 2-fold) enhancement of wild-type oligodendrocytes (Figure S6A).

### Mechanistic Studies Reveal a Non-canonical, UPR-Mediator Role for Ro 25–6981 in Differentiating *Jimpy* OPCs

Ro 25–6981 is an NR2B-selective N-methyl-D-aspartate (NMDA) receptor antagonist (Fischer et al., 1997). However, we noted absent expression in our RNA-seq data profiling OPC and early oligodendrocyte differentiation in all iPSC-derived wild-type and *jimpy* cell cultures (fragments per kilobase of transcript per million mapped reads < 0.15; Table S1), consistent with publicly available datasets (Zhang et al., 2014). Still, a handful of studies have documented expression of the NR2B subunit in oligodendrocytes and astrocytes in normal and pathological contexts (Karadottir et al., 2005, Krebs et al., 2003, Salter and Fern, 2005).

To address the possibility of an NR2B-mediated effect we first sought to investigate *Grin2b* (NR2B subunit gene) expression throughout the OPC to oligodendrocyte transition using time course qRT-PCR in iPSC-derived *jimpy* and wild-type cultures. This comprehensive profiling failed to detect *Grin2b* expression at any time point (Figure S6C). We also performed functional testing of 29 unique NMDA receptor modulators (including NR2B-selective and pan-NMDA receptor antagonist drugs with high potency to their annotated targets) in 10-point dose response (from 10 μM to 20 nM) on iPSC-derived *jimpy* OPCs. These data failed to establish a correlation between NR2B-NMDA receptor antagonism and the enhancement of *jimpy* oligodendrocyte survival (Table S6). Furthermore, structure-activity relationship analysis revealed that only the Ro 25–6981 scaffold could potently increase *jimpy* oligodendrocyte survival. Together, these data eliminated the NR2B-NMDA receptor as the functional target in *jimpy* cultures.

**Figure 5:**
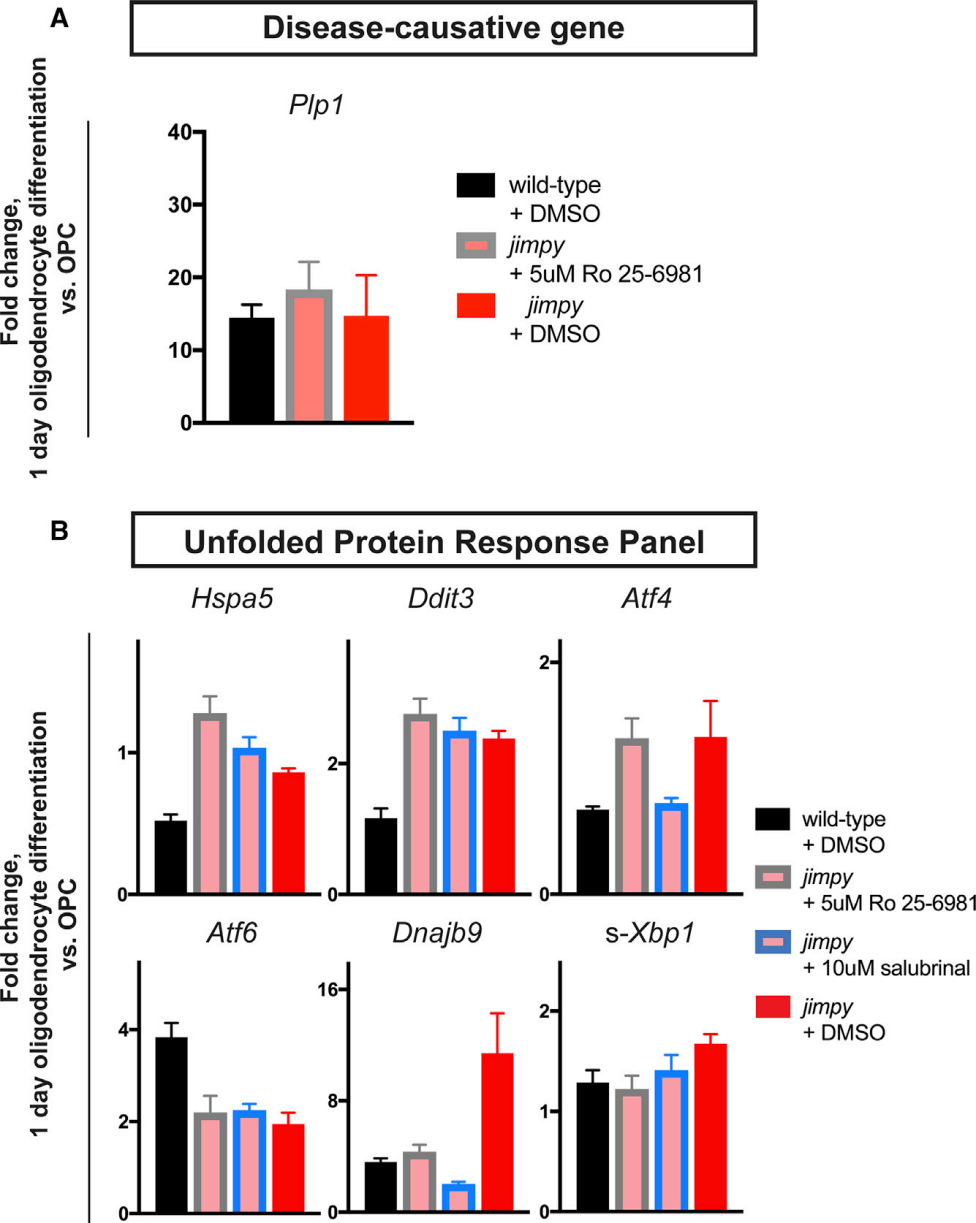
Ro 25–6981 Mechanistic Studies Reveal UPR Modulation in *Jimpy* (A) qRT-PCR of *Plp1* for wild-type and *jimpy* at day 1 of oligodendrocyte differentiation with indicated treatment. Vehicle-treated controls same as Figure S6D. For *Atf6* n = 3 technical replicates per sample. n = 4 technical replicates per sample. (B) qRT-PCR of UPR-related panel for wild-type and *jimpy* at day 1 of oligodendrocyte differentiation with indicated treatment. Vehicle-treated controls same as Figure S6D. For *Atf6* n = 3 technical replicates per sample. For all other probes n = 4 technical replicates per sample. Error bars represent mean ± SD. See also Figure S6 and Table S6.

To investigate alternative mechanisms for Ro 25–6981's action on *jimpy* oligodendrocyte survival *in vitro* we employed targeted qRT-PCR assessment during *jimpy* and wild-type oligodendrocyte differentiation. First we assessed Ro 25–6981's ability to alter *Plp1* expression levels as reduction of mutant PLP is beneficial to PMD cellular and animal models (Karim et al., 2010, Prukop et al., 2014). We found no effect of Ro 25–6981 on *Plp1* induction in *jimpy* (Figure 5A). We next examined Ro 25–6981's ability to modulate *jimpy*-dysregulated UPR-related factors curated from our transcriptome data including *Ddit3*, *Hspa5*, *Dnajb9*, spliced*-Xbp1* (s*-XBP1*), *Atf4*, and *Atf6*. As a comparator we also assessed the effect of the known UPR modulator salubrinal. Both compounds showed reductions of *Dnajb9* and *s-Xbp1* to wild-type levels, no change in *Atf6*, and increased expression of *Hspa5* (Figure 5B). Of note, salubrinal restored *Atf4* to wild-type levels while Ro 25–6981 showed no change. In contrast Ro 25–6981 modestly increased Ddit3, above the salubrinal-mediated effect (Figure 5B). Interestingly, while these compounds produced remarkably similar profiles, their points of divergence may provide a clue to their vastly different efficacies in *jimpy* oligodendrocyte restoration. The *Ddit3* and *Hspa5* results are especially intriguing as depletion of DDIT3 has been shown to exacerbate disease in PMD animal models (Southwood et al., 2002), while reduction of HSPA5 is deleterious to oligodendrocyte survival in both normal and autoimmune disease contexts (Hussien et al., 2015) and is depleted in PMD models (Numata et al., 2013).

We further examined these UPR expression effects testing Ro 25–6981 as well as salubrinal on differentiating wild-type cultures. Surprisingly the vast majority of the transcriptional changes were *jimpy* specific, with *Atf6* and *Ddit3* even showing completely opposite trends in the Ro 25–6981 condition (Figure S6D). Together these data suggested that Ro 25–6981 functioned by modulating the UPR in a genotype-specific manner that was highly specific to *jimpy* cultures.

### Ro 25–6981 Increases Oligodendrocytes in Human PMD Patient iPSC-Derived Cultures and *Jimpy* Mice

**Figure 6:**
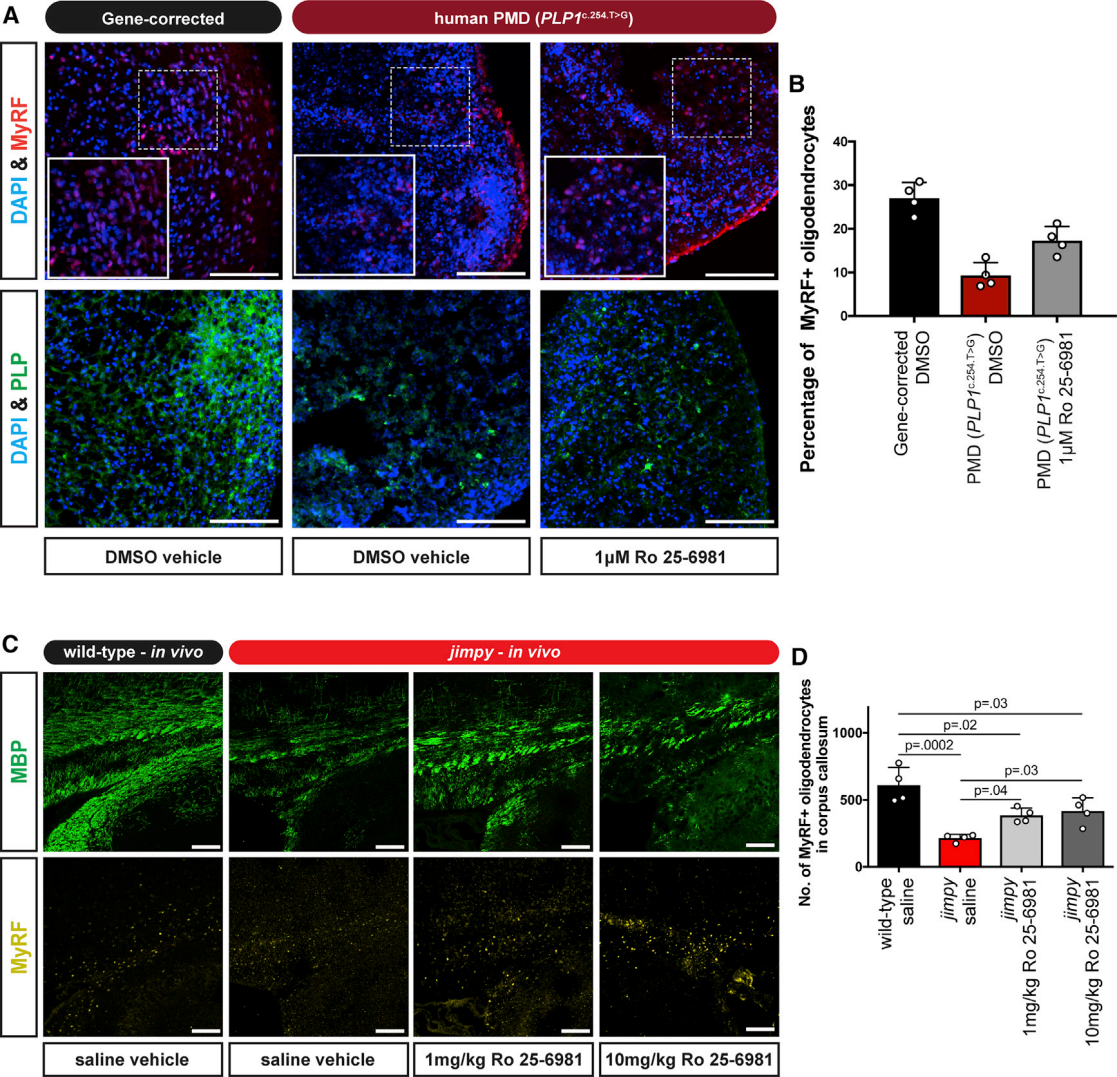
Ro 25–6981 Enhances Mutant Oligodendrocyte Survival in Human Patient PMD Spheroids and in *Jimpy* Mice (A) Representative immunocytochemistry images of human PMD patient (*PLP1*^c.254.T>G^) and wild-type iPSC-derived oligocortical spheroids showing MyRF+ (red) and PLP+ (green) oligodendrocytes, as well as total DAPI+ cells (blue) after treatment. (B) Percentage of MyRF+ oligodendrocytes relative to total DAPI+ cells in human PMD patient (*PLP1*^c.254.T>G^) and wild-type iPSC-derived oligocortical spheroids after treatment. n = 4 independent spheroids per treatment. (C) Representative immunocytochemistry images of the medial corpus callosum of wild-type and *jimpy* mice after treatment showing MBP+ oligodendrocytes (green) and MyRF+ oligodendrocytes (yellow). (D) Quantification of MyRF+ oligodendrocytes within the medial corpus callosum of wild-type and *jimpy* mice after treatment. n = 4 injected animals per treatment. Scale bars, 100 μm. Error bars represent mean ± SD, with each replicate value indicated by a white circle. p values calculated using a one-way ANOVA with Holm-Sidak correction for multiple comparisons.

To test if Ro 25–6981's effect was mutation or organism specific, we applied this compound to human PMD oligocortical spheroids (Madhavan et al., 2018) generated using iPSCs from a severe connatal PMD patient with a *PLP1*^c.254T>G^ point mutation (Nevin et al., 2017). We applied 1 μM Ro 25–6981 or DMSO vehicle to mutant spheroids during a 20-day period of oligodendrocyte generation and quantified oligodendrocytes as a percentage of total cell number using an antibody to the oligodendrocyte-specific transcription factor, myelin regulatory factor (MyRF). In addition, we administered DMSO vehicle to control, isogenic gene-corrected spheroids. Ro 25–6981 treatment profoundly increased the percentage of mutant oligodendrocytes in the human PMD spheroids (Figures 6A and 6B), confirming the ability to target common cellular pathology and enhance mutant oligodendrocyte survival across distinct PMD genotypes and species. Of note these levels of oligodendrocytes were still below those of the isogenic control, suggestive of remaining pathology, potentially also reflected by the unrestored PLP expression pattern (Figure 6A).

Next we examined if Ro 25–6981 treatment could increase oligodendrocytes in *jimpy* mice, *in vivo*. We administered 1 or 10 mg/kg Ro 25–6981 or saline vehicle to *jimpy* mice and saline vehicle to wild-type mice from postnatal days 5–14, and quantified MyRF+ oligodendrocytes along the medial rostral caudal axis of the corpus callosum. Ro 25–6981 treatment significantly increased *jimpy* oligodendrocytes in a dose-dependent manner, but below wild-type levels (Figures 6C and 6D). These results confirmed that Ro 25–6981's effect was not limited to *in vitro* contexts and could enhance oligodendrocyte survival in *jimpy* mice *in vivo*.

### Increasing *Jimpy* Oligodendrocyte Survival Uncovers a Second Pathological Phase during Myelination

Oligodendrogenesis is thought to be closely coupled to myelination (Mayoral and Chan, 2016)—a process which involves the generation and extension of specialized oligodendrocyte membrane around neuronal axons. Given the strong enhancement of early oligodendrocyte survival across multiple contexts we expected to see a frank restoration of myelinating oligodendrocytes. Surprisingly we found only a slight increase in myelinating cells in Ro 25–6981-treated *jimpy* mice as marked by MBP (Figure 6C).

**Figure 7:**
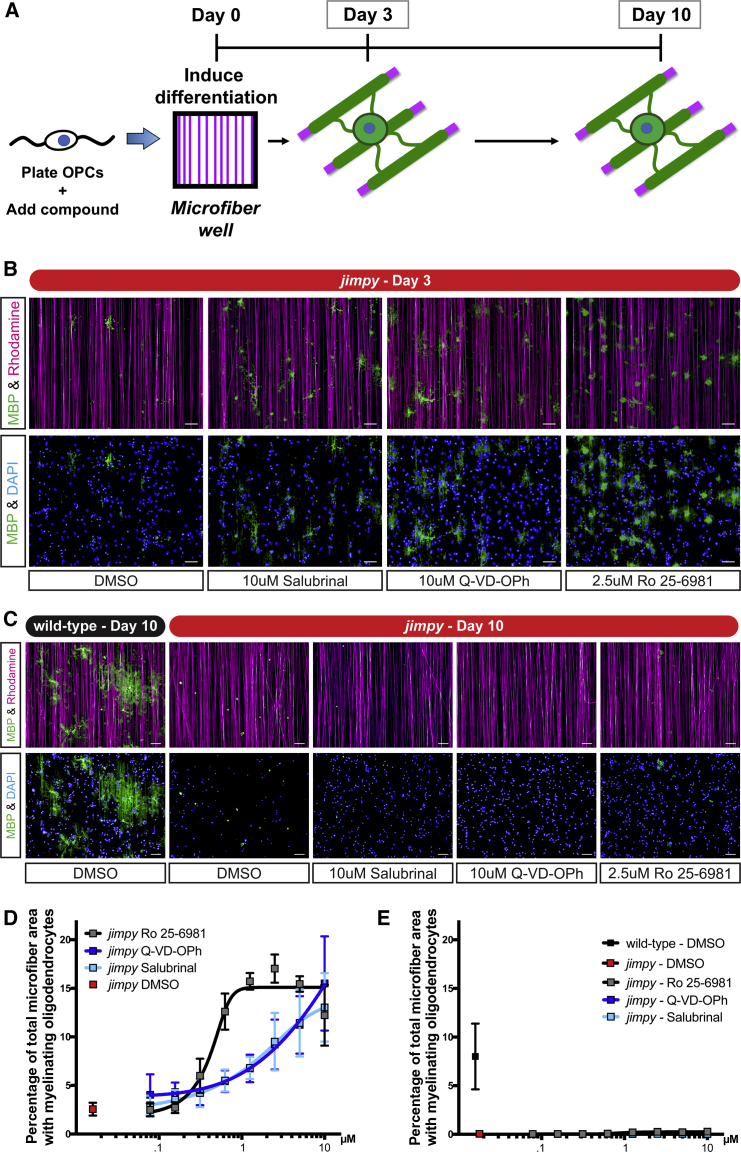
Increased *Jimpy* Oligodendrocyte Survival during Differentiation Unmasks a Second Pathological Phase during Myelination (A) Schematic of the *in vitro* myelination assay using 4-μm diameter synthetic microfiber fibers detailing the examination of myelinating oligodendrocytes at day 3 and 10. (B and C) Representative immunocytochemistry images of MBP+ myelinating oligodendrocytes (green), rhodamine+ microfibers (purple), and total DAPI+ cells (blue) at (B) day 3 and (C) day 10. Compound-treated cultures shown at their maximum effective concentration. (D and E) Quantification of percent total microfiber area overlap with MBP+ myelinating oligodendrocytes at (D) day 3 and (E) day 10 with indicated treatment and genotype. Scale bars, 50 μm. n = 3 replicate wells per compound treatment. n = 16 replicate wells per vehicle control. Error bars represent mean ± SD. See also Figure S7.

To appreciate why increased oligodendrocyte survival did not translate to a broad restoration of myelin we employed a defined system using non-biological poly-lactide microfibers. These microfibers serve as a robust, reproducible, and scalable myelination substrate (Bechler et al., 2015). We plated *jimpy* OPCs in the presence of Ro 25–6981 in an 8-point (10 μM to 78 nM) dose-response format or DMSO vehicle. To account for Ro 25-6981-specific effects on myelination we also tested our *jimpy* cellular pathology positive control compounds, salubrinal and Q-VD-OPh. We induced oligodendrocyte differentiation, allowed for microfiber ensheathment (*in vitro* “myelination”), and quantified MBP+ myelinating oligodendrocytes (Figure 7A) using an automated image analysis pipeline (Figures S7A–S7E). Despite showing a robust, dose-dependent increase in *jimpy* oligodendrocyte survival and early myelination (Figures 7B and 7D), Ro 25–6981, salubrinal, and Q-VD-OPh were unable maintain myelin by day 10 across all tested doses. This result was specific to *jimpy*, as wild-type DMSO vehicle-treated cultures showed extensive myelination at day 10 (Figures 7C and 7E). The ability to restore *jimpy* oligodendrocytes without a subsequent restoration of mutant myelin was unexpected and revealed that there are two distinct stages of disease pathology: (1) ER stress-mediated cell death as cells transition from OPCs to oligodendrocytes and (2) dysfunctional myelination due to the presence of mutant PLP protein in surviving oligodendrocytes. Collectively, our results show that the first phase can be resolved with small molecules and that effective clinical intervention for PMD will need to address both phases of pathology.

## Discussion

In this study we established a genotype-specific, scalable, and tractable system to investigate a severe PMD point mutation modeled in the *jimpy* mouse. Coupled with stringent control over oligodendrocyte differentiation, this system enabled precise, temporal dissection of *jimpy* pathology. Using a simple developmental trigger (induction of oligodendrocyte differentiation) we recapitulated known cellular and molecular PMD phenotypes including ER stress and oligodendrocyte death (Ikeda et al., 2018, Southwood et al., 2002), and defined a surprisingly early susceptibility period. Chemical screening demonstrated a reversal of *jimpy* oligodendrocyte loss by inhibiting specific targets in the UPR and apoptosis pathways. Furthermore, this system allowed us to identify Ro 25–6981, a highly effective and potent modulator of *jimpy* pathology with a previously undefined effect on the UPR.

While our studies were designed to identify cellular and molecular phenotypes as well as compound modulators on a genotype-specific basis, we reasoned that UPR effectors could counteract the sequelae seen in alternate, human PMD mutations by targeting common pathology in this disease (Nevin et al., 2017). We tested Ro 25–6981 on PMD patient-derived human oligocortical spheroids and demonstrated a robust increase in mutant oligodendrocytes, confirming efficacy in human disease-relevant tissue and suggesting modulation of shared disease components.

Given the striking enhancement of mutant oligodendrocytes across multiple contexts (cellular, genotypic, and species), we expected to observe a substantial increase in myelin after Ro 25–6981 treatment, *in vivo*. While Ro 25–6981 increased the quantity of *jimpy* oligodendrocytes, it did not restore myelination to wild-type levels. As oligodendrogenesis and myelination are thought to be distinct processes (Mayoral and Chan, 2016), we examined myelination in *jimpy* with Ro 25–6981 treatment using non-biologic, synthetic microfibers. These data revealed that Ro 25–6981 was unable to restore myelination in *jimpy*, revealing a second stage of disease, refractory to treatment that enhances early oligodendrocyte survival. As such, our results establish a dual therapeutic paradigm: (1) restore oligodendrocyte loss during differentiation and then (2) enhance myelination capacity of surviving, mutant oligodendrocytes. Importantly, approaches that simply target these rare, surviving oligodendrocytes are unlikely to be successful without first enhancing the survival during the early OPC to oligodendrocyte transition. This understanding has substantial impact on therapeutic development for PMD and potentially other disorders where ER stress is implicated.

The dichotomy of compound-mediated increased mutant oligodendrocyte survival without increased myelination could be explained by the unique demands of the latter process, which involves immense synthesis of myelin lipids and proteins (Baron and Hoekstra, 2010), including PLP which has been estimated to constitute approximately 50% of the total myelin protein (Baumann and Pham-Dinh, 2001). As such the myelination stage may necessitate engagement of completely separate targets for pharmacological interventions. Prior studies by us and others have shown, paradoxically, that modulating opposing responses in the UPR pathway, including inhibition of PERK or GADD34, leads to phenotypic benefit in PMD and other myelinating disorders (D'Antonio et al., 2013, Musner et al., 2016, Nevin et al., 2017). Future studies will need to parse the precise target of Ro 25–6981's UPR-modulating effect and assess the potential use of combinatorial therapies.

In addition, the presence mutant PLP protein could lead to specific sequelae (Koizume et al., 2006, Kramer-Albers et al., 2006, Roussel et al., 1987), distinct from the pathology that develops immediately after *jimpy* OPCs begin to differentiate to oligodendrocytes. Interestingly, *PLP1* duplications, which constitute ∼70% of human patients (Inoue, 2005), may be especially amendable to UPR-modulating compounds such as Ro 25–6981, as these cases are typified by overly abundant but normal PLP protein. As such, moderating ER overload may be a viable strategy to restore oligodendrocytes and myelin in these patients.

More broadly, given that ER stress is a fundamental component of many diseases of myelin including other leukodystrophies and multiple sclerosis (Clayton and Popko, 2016, Way et al., 2015), *jimpy* modulators such as Ro 25-6981 may provide effects beyond PMD, to other genetic disorders of myelin and multiple sclerosis.

## Experimental Procedures

### Animal Studies

All animal procedures and experimentation was approved by Case Western Reserve University’s Institutional Animal Care and Use Committee.

### Generation of *Jimpy* iPSCs

Tail-tip fibroblasts were derived from *jimpy* mutant and wild-type littermates reprogrammed to iPSCs using a lentivirus encoding a doxycycline-inducible polycistronic Oct4, Sox2, Klf4, and c-Myc construct as described previously (Somers et al., 2010, Sommer et al., 2009).

### Generation of *jimpy* OPCs

iPSCs were differentiated to high purity, expandable OPCs as described previously (Najm et al., 2011) (Lagar et al., 2018). OPCs were propagated in DMEM/F12 (11320082, Thermo Fisher Scientific), 1× N2 supplement (AR009, R&D Systems), 1× B-27 without vitamin A supplement (12,587-010, Thermo Fisher Scientific), and 1× Glutamax, supplemented with 20 ng/mL fibroblast growth factor 2 (233-FB, R&D Systems) and 20 ng/mL platelet-derived growth factor-AA (221-AA, R&D Systems).

### Differentiation of *Jimpy* OPCs to Oligodendrocytes

OPCs were differentiated to oligodendrocytes with differentiation medium that consisted of DMEM/F12, 1× N2 supplement, 1× B-27 without vitamin A supplement, supplemented with 100 ng/mL noggin (3344-NG, R&D Systems), 10 ng/mL neurotrophin-3 (NT-3) (267-N3, R&D Systems), 50 μM cAMP (D0260, Sigma), 100 ng/mL insulin-like growth factor-1 (291-G1, R&D Systems) NT-3, and 40 ng/mL triiodothyronine (thyroid hormone; T-6397, Sigma).

### Compound Screening

OPCs were seeded on 384-well poly-D-lysine CellCarrier Ultra plates (6057500, PerkinElmer), compound was added, and oligodendrocyte differentiation was induced. Three days later plates were fixed with 4% paraformaldehyde (PFA), and immunostained using rat anti-MBP and goat anti-Sox10, followed by counterstaining with DAPI. Images were captured and quantified using the Operetta High Content Imaging and Analysis system (PerkinElmer), Harmony software (PerkinElmer), and Acapella software (PerkinElmer).

### qRT-PCR

RNA was prepared with the RNeasy Mini Kit (74104, QIAGEN). qRT-PCR was performed using pre-designed TaqMan gene expression assays (Thermo Fisher Scientific) and probes values were normalized to an *Actb* endogenous control.

### Human PMD Cultures

Human PMD patient (*PLP1*^c.254T>G^) and gene-corrected (isogenic control) iPSCs were previously generated as described (Nevin et al., 2017) and used to generate oligocortical spheroids (Madhavan et al., 2018). Spheroids were treated with DMSO vehicle or Ro 25–6981 from days 60 to 90. Spheroids were fixed with 4% PFA, and immunostained using rabbit anti-MyRF and rat anti-PLP, followed by counterstaining with DAPI. Images were captured and quantified using Adobe Photoshop (Abobe Systems).

### In Vivo *Jimpy* Studies

*Jimpy* and wild-type control mice were administered Ro 25–6981 (1594, R&D Systems) or saline vehicle from postnatal day 5 to 14. Mice were sacrificed and tissue sections were immunostained using rabbit anti-MyRF and rat anti-MBP antibody. Complete corpus callosum (medial, sagittal sections) images were captured and quantified using Adobe Photoshop.

### *In Vitro* Myelination Assay

OPCs were seeded on Mimetix 384-well plates containing synthetic microfibers (AMS-TECL-015, AMSBio), compound was added, and oligodendrocyte differentiation was induced. At day 3 and 10 after seeding, plates were fixed with 4% PFA, and immunostained using rat anti-MBP, followed by counterstaining with DAPI. Images were captured and quantified using the Operetta High Content Imaging and Analysis system, Harmony software, and Acapella software.

### Quantitation and Statistical Analyses

GraphPad Prism was used to perform all statistical analyses unless otherwise indicated. Statistical tests as well as replicate descriptions are detailed in each figure legend. A p value < 0.05 was considered significant for all analyses, unless otherwise denoted.

## Author Contributions

M.S.E. and P.J.T. conceived and initiated the project. H.E.S. and M.S.E. harvested animal tissues. D.C.F. and T.E.M. generated and analyzed the RNA-seq data, and generated the associated figures. C.W., Y.L., and F.J. performed Drop-Seq, data analysis, and generated the associated figures. B.L.L.C. and M.S.E. executed the astrocyte studies. K.C.A., L.B., and M.S.E. performed qRT-PCR and analyzed the data. H.E.S., M.M., and M.S.E. isolated primary cell cultures. D.J.A. and M.S.E. designed the high-throughput primary compound screen and analyzed the data. M.M., L.B., and B.S.N. carried out immunohistochemistry and quantifications. M.M., Z.S.N, M.S.E., and H.E.S. cultured human spheroids and generated associated figures. M.S.E. and P.J.T. wrote the manuscript with feedback from all authors.

## Accession Numbers

The accession number for the RNA-seq data reported in this paper is GEO: GSE111605.

## Supplemental Information

Document S1. Supplemental Experimental Procedures, Figures S1–S7, and Tables S5 and S6

Table S1. *Jimpy* Pathway Enrichment during Bulk Culture Oligodendrocyte Differentiation, Related to Figure 2 GSEA of bulk culture RNA-seq showing enriched pathways in *jimpy* OPCs relative to wild-type OPCs, after 1 day of oligodendrocyte differentiation.

Table S2. Differential Expression between Wild-Type and *Jimpy* Cell Types during Oligodendrocyte Differentiation, Related to Figure 2 scRNA-seq data showing differentially expressed genes (p < 01) between wild-type and *jimpy* OPCs, oligodendrocytes, and astrocytes, respectively, after 1 day of oligodendrocyte differentiation.

Table S3. *Jimpy* Cell-Type Specific Pathway Enrichment during Oligodendrocyte Differentiation, Related to Figure 2 GSEA of scRNA-seq showing enriched pathways and corresponding p values in *jimpy* OPCs, oligodendrocytes, and astrocytes relative to their respective wild-type counterparts.

Table S4. Primary Compound Screening Results, Related to Figure 4 Primary (>3,000) bioactive compound screening data showing results of each tested compound.

Document S2. Article plus Supplemental Information
