# Supplementary material for: Chemical Screening Identifies Enhancers of Mutant Oligodendrocyte Survival and Unmasks a Distinct Pathological Phase in Pelizaeus-Merzbacher Disease
Source: Stem Cell Reports. 2018 Aug 23;11(3):711–26. doi: 10.1016/j.stemcr.2018.07.015 (PMC6135742; doi:10.1016/j.stemcr.2018.07.015)
Supplement: Document S1. Supplemental Experimental Procedures, Figures S1–S7, and Tables S5 and S6 [file mmc1.pdf]

Stem Cell Reports, Volume *11*

Supplemental Information

Chemical Screening Identifies Enhancers of Mutant Oligodendrocyte Survival and Unmasks a Distinct Pathological Phase in Pelizaeus-Merzbacher Disease

Matthew S. Elitt, H. Elizabeth Shick, Mayur Madhavan, Kevin C. Allan, Benjamin L.L. Clayton, Chen Weng, Tyler E. Miller, Daniel C. Factor, Lilianne Barbar, Baraa S. Nawash, Zachary S. Nevin, Angela M. Lager, Yan Li, Fulai Jin, Drew J. Adams, and Paul J. Tesar

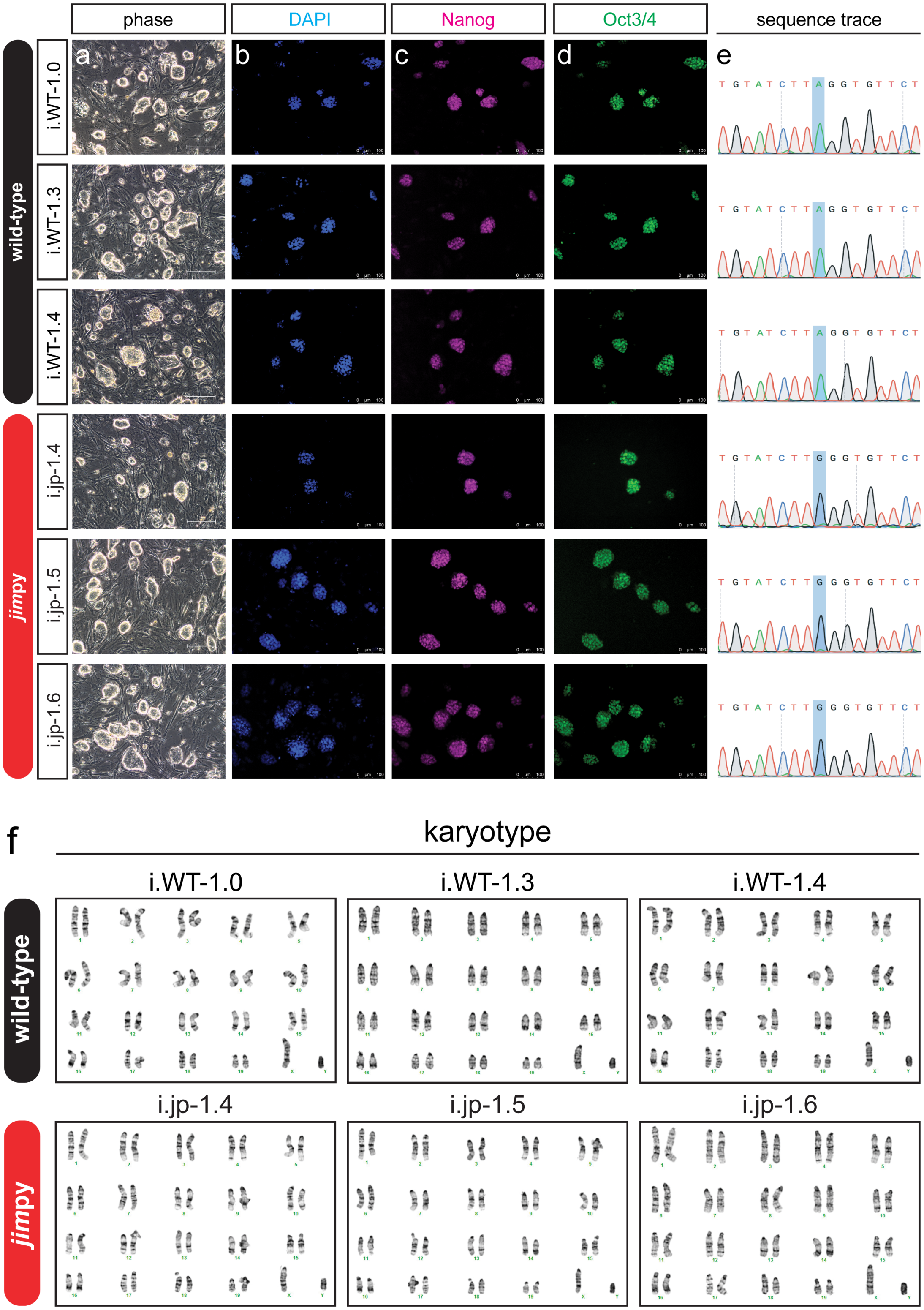
phase
DAPI
Nanog
Oct3/4
sequence trace
a
b
c
d
e
wild-type
i.WT-1.0
i.WT-1.3
i.WT-1.4
jimpy
i.jp-1.4
i.jp-1.5
i.jp-1.6
f
karyotype
i.WT-1.0
i.WT-1.3
i.WT-1.4
wild-type
i.jp-1.4
i.jp-1.5
i.jp-1.6
jimpy

Figure S1. Wild-type and *jimpy* iPSCs exhibit grossly normal morphology and karyotypes, and express canonical pluripotency markers. Related to Figure 1

(a) Representative phase contrast images of wild-type and *jimpy* iPSC lines. Scale bar, 200µm. **(a-d)** Representative immunocytochemistry images of wild-type and *jimpy* iPSC lines showing **(b)** DAPI, **(c)** Nanog, and **(d)** Oct3/4 staining. Scale bar, 100µm. **(e)** 5' to 3' Sanger sequencing traces showing *jimpy* mutation status (highlighted with blue bar, A=wild-type and G=*jimpy*) for wild-type and *jimpy* iPSC lines. **(f)** Metaphase spreads for wild-type and *jimpy* iPSC lines displaying grossly normal karyotypes. Three lines exhibited loss or gain of the Y chromosome, a common occurrence in mouse iPSC lines, in a subset of cells (2, 5, 6, and 2 out of 20 for i.wt-1.0, i.wt-1.4 i.jp-1.4, i.jp-1.5, respectively).

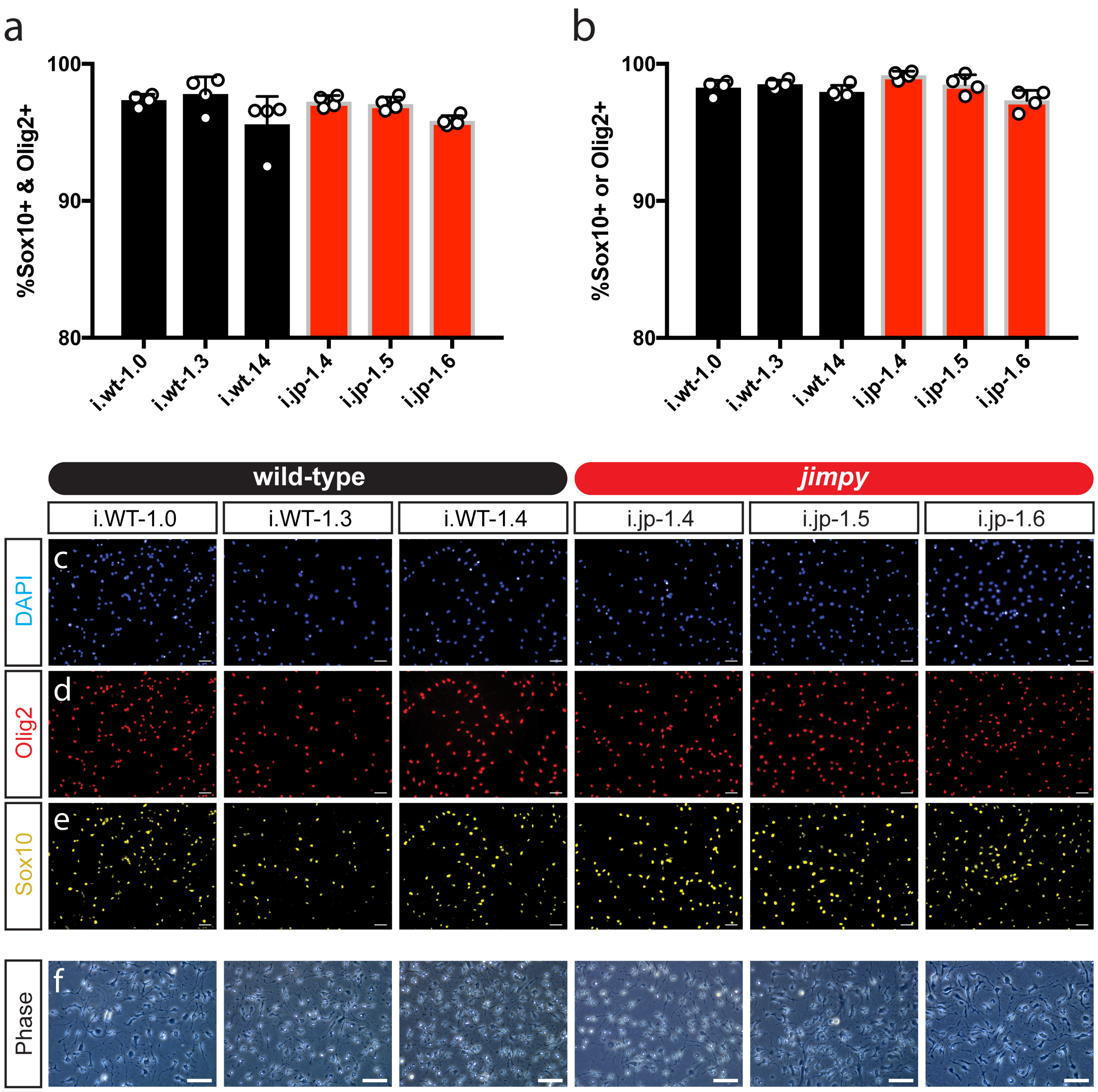

Figure S2. Wild-type and *jimpy* iPSC-derived OPCs lines are highly pure based on canonical OPC markers expression. Related to Figure 1

(a) Percentage of double Sox10+ & Olig2+ OPCs in wild-type (black) and *jimpy* (red) cell lines. **(b)** Percentage of Sox10+ or Olig2+ OPCs in wild-type (black) and *jimpy* (red) cell lines. **(c-e)** Representative immunocytochemistry images of wild-type and *jimpy* OPCs lines showing **(c)** DAPI, **(d)** Olig2, and **(e)** Sox10 staining. Scale bar, 50µm. **(f)** Representative phase contrast images of wild-type and *jimpy* OPCs lines. Scale bar, 100µm**.** Error bars represent mean ± SD. n=4 replicate wells quantified for each cell line. Each replicate value represented by a white circle.

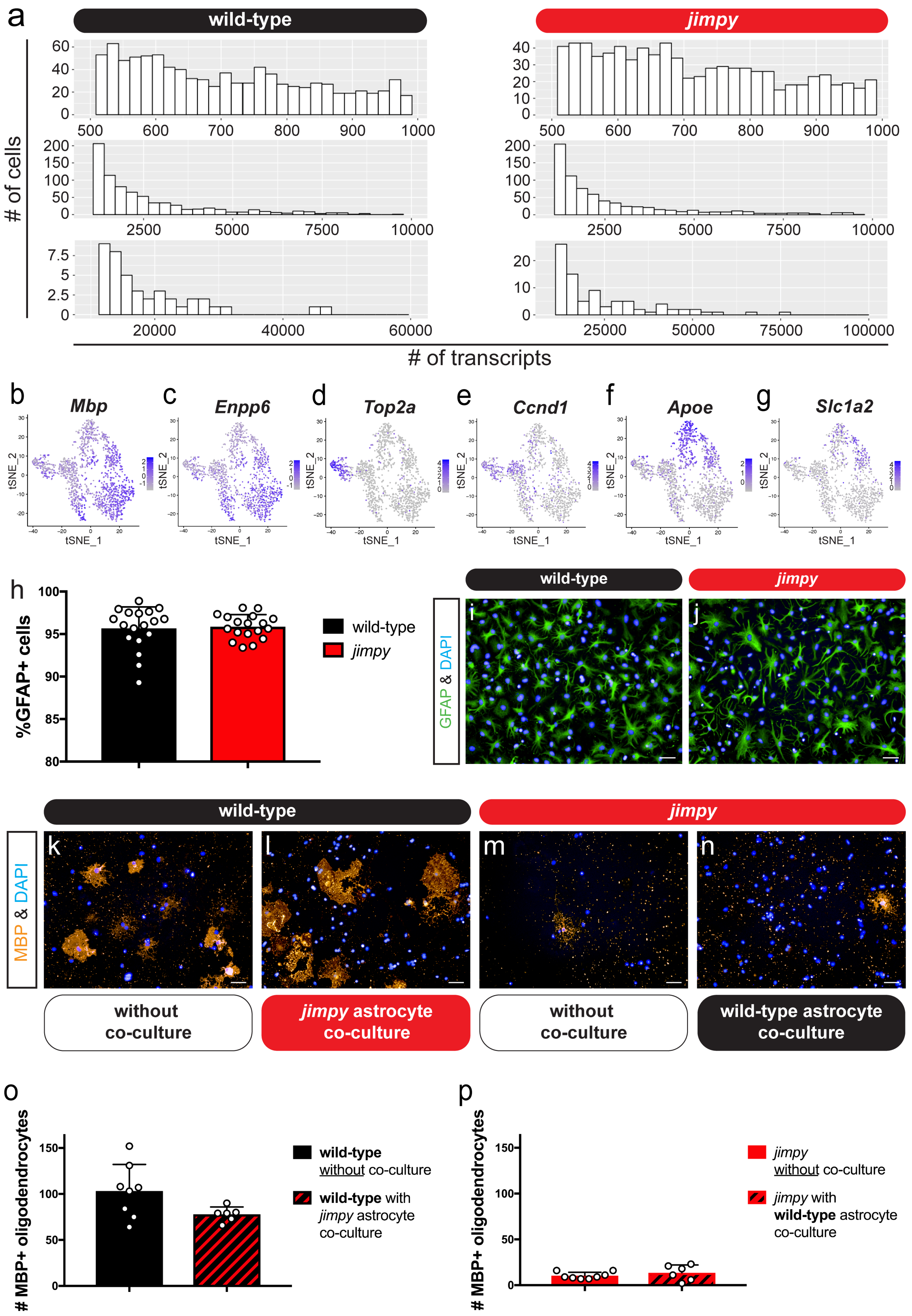

a
wild-type
jimpy
# of cells
# of transcripts
b Mbp
c Enpp6
d Top2a
e Ccnd1
f Apoe
g Slc1a2
tSNE_1
tSNE_2
h
%GFAP+ cells
wild-type
jimpy
GFAP & DAPI
i
j
MBP & DAPI
k
l
m
n
without co-culture
jimpy astrocyte co-culture
without co-culture
wild-type astrocyte co-culture
o
MBP+ oligodendrocytes
wild-type without co-culture
wild-type with jimpy astrocyte co-culture
p
jimpy without co-culture
jimpy with wild-type astrocyte co-culture

Figure S3. scRNA-seq quality control metrics and astrocyte characterization. Related to Figure 2
(a) Histogram of individual transcripts sequenced per cell at day one of oligodendrocyte differentiation for wild-type and *jimpy* cultures. **(b-g)** tSNE plots of both wild-type and *jimpy* cells at day one of oligodendrocyte differentiation showing normalized expression levels of **(b)** *Mbp* or **(e)** *Enpp6*, **(c)** *Top2a* or **(f)** *Ccnd1*, and **(d)** *Apoe* or **(g)** *Slc1a2* to inform identification of nascent oligodendrocytes, OPCs, and nascent astrocytes, respectively, after unsupervised clustering. **(h)** Quantification of %GFAP+ cells after 3 day astrocyte differentiation of iPSC-derived wild-type and *jimpy* OPCs. n=18 replicate wells per genotype. **(i-j)** Representative immunocytochemistry images after 3 day astrocyte differentiation of iPSC-derived **(i)** wild-type and **(j)** *jimpy* OPCs showing GFAP+ astrocytes (green) and total DAPI+ cells (blue). **(k-n)** Representative immunocytochemistry images after 3 day oligodendrocyte differentiation of iPSC-derived wild-type and *jimpy* OPCs showing MBP+ oligodendrocytes (orange) and total DAPI+ cells (blue) in wild-type cultures **(k)** alone and **(l)** with *jimpy* astrocyte co-culture and in *jimpy* cultures **(m)** alone and **(n)** with wild-type astrocyte co-culture. **(o)** Quantification of MBP+ oligodendrocytes after 3 day oligodendrocyte differentiation of iPSC-derived wild-type OPCs with (n=6 replicate wells) and without (n=8 replicate wells) *jimpy* astrocyte co-culture. **(p)** Quantification of MBP+ oligodendrocytes after 3 day oligodendrocyte differentiation of iPSC-derived *jimpy* OPCs with (n=6 replicate wells) and without (n=8 replicate wells) wild-type astrocyte co-culture. Error bars represent mean ± SD. Each replicate value represented by a white circle.

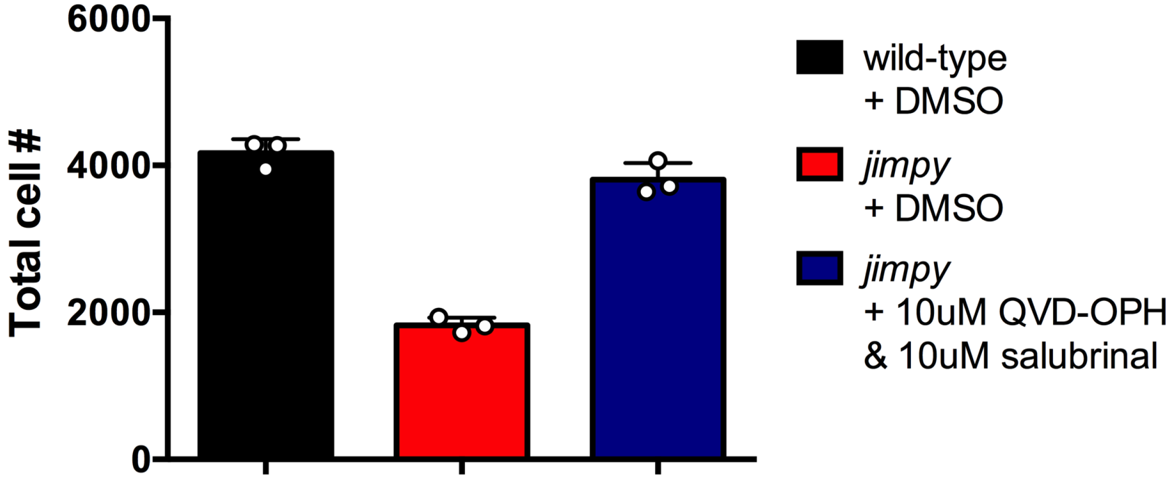

Figure S4. Q-VD-OPh and salubrinal, in combination, restore *jimpy* cell number back to wild-type levels. Related to Figure 3.
Quantification of total cell number after 3 day oligodendrocyte differentiation of wild-type OPCs treated with DMSO vehicle, and *jimpy* OPCs treated with DMSO vehicle or 10µM Q-VD-OPh & 10µM salubrinal. n=3 replicate wells per treatment. Error bars represent mean ± SD. Each replicate value represented by a white circle.

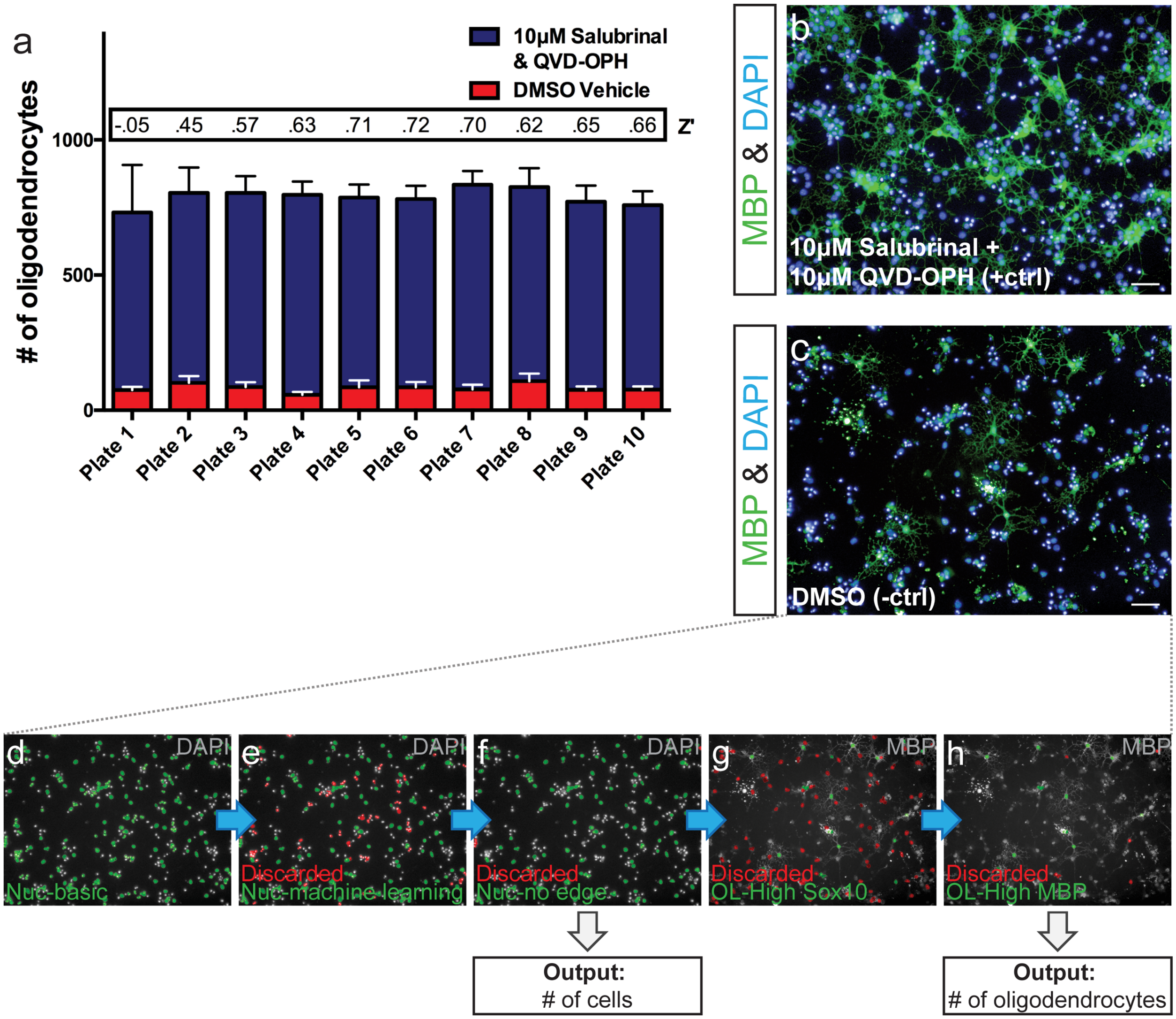

Figure S5. Primary screening controls and automated image analysis display robust performance. Related to Figure 4

(a) Primary screen negative control (DMSO vehicle) and positive control (10µM Q-VD-OPh and 10µM salubrinal) metrics on a per plate basis with corresponding Z' factor for oligodendrocyte number. n=16 replicate wells per control, per screening plate. Error bars represent mean ± SD. **(b and c)** Representative immunocytochemistry images of primary screen controls for **(b)** 10µM Q-VD-OPh and 10µM salubrinal and **(c)** DMSO vehicle treated *jimpy* OPCs after oligodendrocyte differentiation showing oligodendrocytes (MBP, green) and total cells (DAPI, blue). Scale bar, 50µm. **(d to h)** Image analysis pipeline**. (d)** Single-field capture of putative DAPI+ nuclei (green) with a basic nuclei finding analysis, further refined with **(e)** machine learning to discard pyknotic nuclei (red) and **(f)** remove nuclei contacting the image border (red) for a final output of bona-fide DAPI+ nuclei (green), defined as total cell number for the analyzed field. **(g)** DAPI+ nuclei with high levels of Sox10 expression (green) defined a putative oligodendrocyte population which was further refined using **(h)** high MBP expression (green) to define the oligodendrocyte number for the analyzed field.

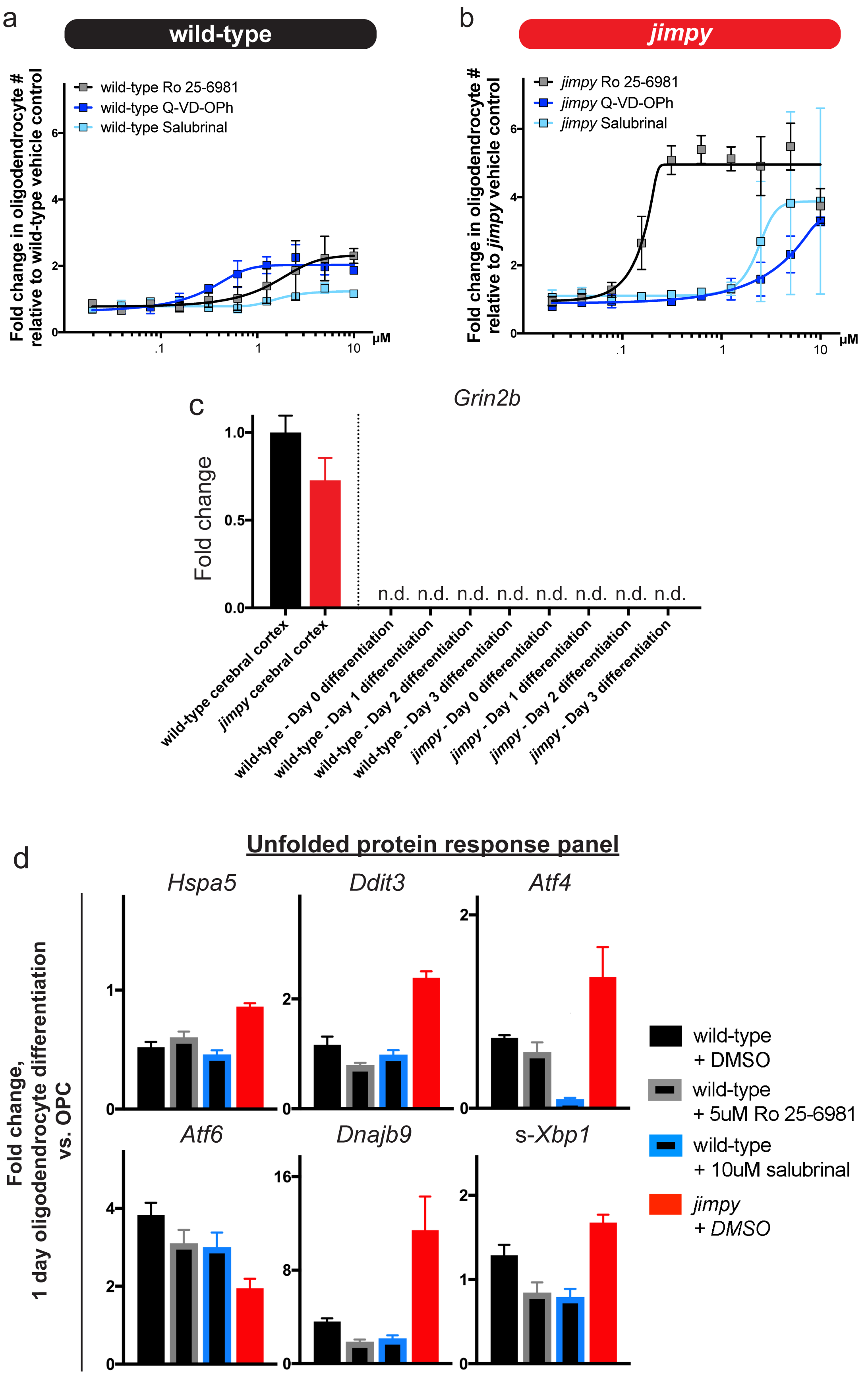

a
wild-type
Fold change in oligodendrocyte # relative to wild-type vehicle control
wild-type Ro 25-6981
wild-type Q-VD-OPh
wild-type Salubrinal
μM
b
jimpy
Fold change in oligodendrocyte # relative to jimpy vehicle control
jimpy Ro 25-6981
jimpy Q-VD-OPh
jimpy Salubrinal
μM
c
Grin2b
Fold change
n.d. n.d. n.d. n.d. n.d. n.d. n.d. n.d.
wild-type cerebral cortex
jimpy cerebral cortex
wild-type - Day 0 differentiation
wild-type - Day 1 differentiation
wild-type - Day 2 differentiation
wild-type - Day 3 differentiation
jimpy - Day 0 differentiation
jimpy - Day 1 differentiation
jimpy - Day 2 differentiation
jimpy - Day 3 differentiation
d
Unfolded protein response panel
Fold change, 1 day oligodendrocyte differentiation vs. OPC
Hspa5
Ddit3
Atf4
Atf6
Dnajb9
s-Xbp1
wild-type + DMSO
wild-type + 5uM Ro 25-6981
wild-type + 10uM salubrinal
jimpy + DMSO

Figure S6. Evaluation of *jimpy* modulators during oligodendrocyte differentiation in wild-type and *Grin2b* expression assessment. Related to Figure 5

(a) Fold change in MBP+ oligodendrocytes (relative to DMSO vehicle control) in 10-point (10µM-20nM) dose response testing for Ro 25-6981, salubrinal, and Q-VD-OPh after 3 day oligodendrocyte differentiation of iPSC-derived wild-type OPCs. n=3 replicate wells per compound treatment. n=16 replicate wells for vehicle control. **(b)** Fold change in MBP+ oligodendrocytes (relative to DMSO vehicle control) in 10-point (10µM-20nM) dose response testing for Ro 25-6981, salubrinal, and Q-VD-OPh after 3 day oligodendrocyte differentiation of iPSC-derived *jimpy* OPCs. n=3 replicate wells per compound treatment. n=16 replicate wells for vehicle control. **(c)** RT-qPCR of *Grin2b* for cerebral cortex harvested from postnatal day 19 wild-type (black) and *jimpy* (red) mice along with differentiating wild-type and *jimpy* OPCs collected at day 0, 1, 2, and 3 of oligodendrocyte differentiation. n=3 technical replicates for wild-type day 0 differentiation. n=4 technical replicates for all other samples. Samples where *Grin2b* was not detected (n.d.) lacked amplification through 36 thermocycles. **(d)** RT-qPCR of *Dnajb9*, *s-Xbp1*, *Hspa5*, *Ddit3*, *Atf4*, *Atf6* for wild-type and *jimpy* OPCs collected at day 1 of oligodendrocyte differentiation with indicated treatment. Vehicle treated controls same as Figure 5. *Atf6* n=3 technical replicates per sample. For all other probes n=4 technical replicates per sample. Error bars represent mean ± SD.

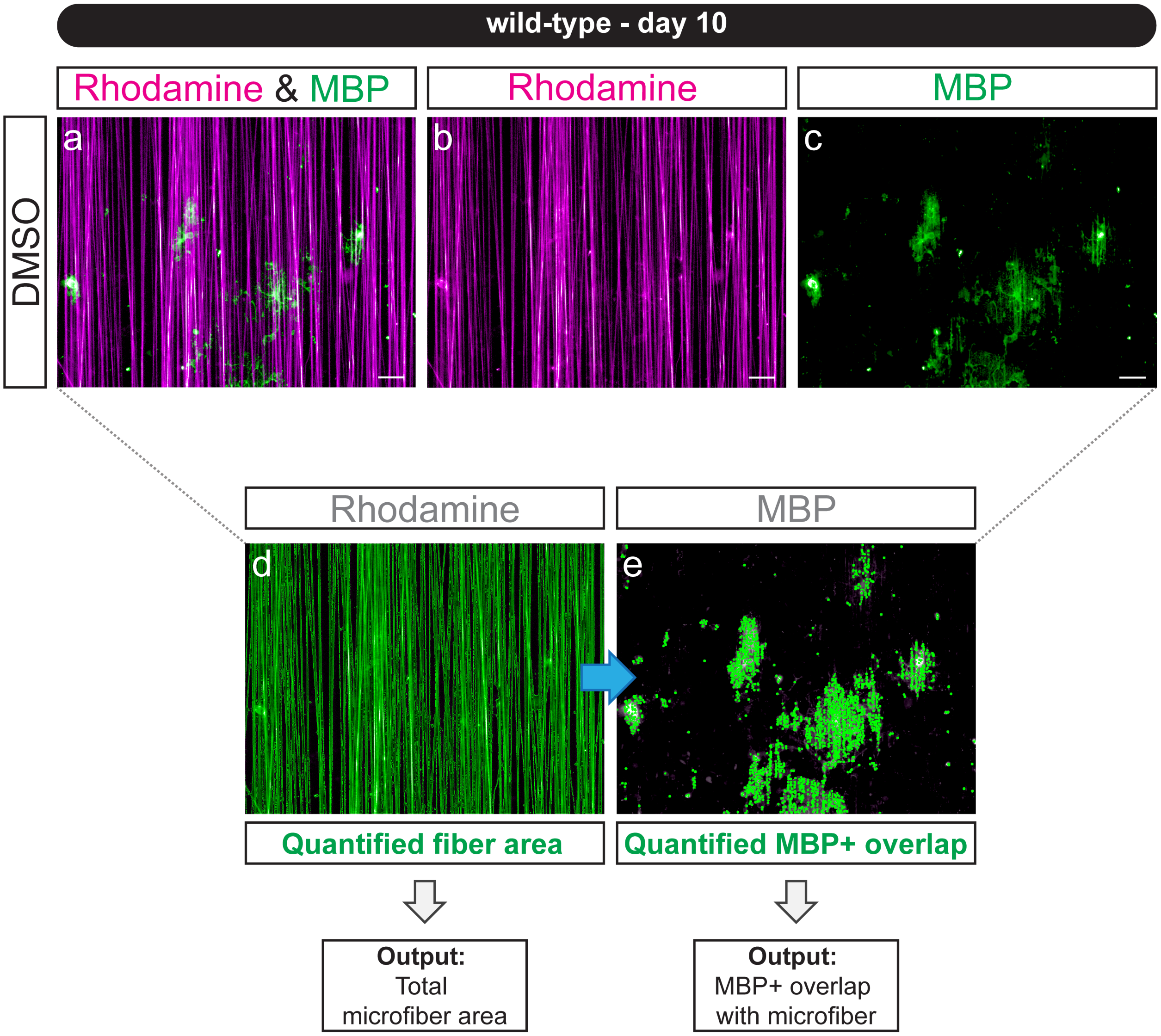

Figure S7. Automated image analysis accurately identifies myelinating oligodendrocytes. Related to Figure 7
(a-c) Single-field capture, representative immunocytochemistry input images are shown with the **(a)** composite of MBP+ myelinating oligodendrocytes (green) with rhodamine+ microfibers (purple) as well as single channel images of **(b)** rhodamine+ microfibers (purple) and **(c)** MBP+ myelinating oligodendrocytes (green) at day 10 for wild-type cultures treated with DMSO vehicle. **(d)** Detection of regions of high rhodamine+ (grey) intensity to define the total microfiber area (green). **(e)** Detection of regions of high MBP+ (grey) intensity to quantify MBP+ overlap with microfibers (green).

Compound Name	1° Screen Rank	Confirm in distinct *jimpy* lines (#)	10 uM	5 uM	2.5 uM	1.25 uM	625 nM	313 nM	156 nM	78 nM	Max. value
Ro 25-6981	45	2	28.6	48.4	50.7	52.0	55.4	53.3	29.2	23.6	**55.4**
Vesamicol	29	2	43.2	43.4	30.3	9.1	10.4	6.1	3.0	3.3	**43.4**
Medroxyprogesterone	32	2	42.9	10.9	8.2	5.9	2.5	-0.9	-1.3	2.1	**42.9**
Bifonazole	2	2	16.9	29.4	41.7	26.1	11.9	9.3	7.6	5.1	**41.7**
Ketoconazole	51	1	32.1	35.9	41.3	12.0	26.6	6.3	17.2	9.0	**41.3**
SB408124	22	2	21.2	20.5	26.5	34.6	40.0	29.5	8.2	1.1	**40.0**
Raltegravir	1	2	38.2	34.6	29.8	14.9	4.0	4.2	1.4	1.6	**38.2**
Estrone	7	2	36.4	31.0	37.4	34.9	35.0	23.0	6.2	-0.2	**37.4**
Fenspiride	15	1	36.6	17.5	15.1	11.5	4.2	6.2	4.9	6.3	**36.6**
EPZ005687	25	2	20.2	21.9	30.8	34.2	21.4	14.5	8.8	1.7	**34.2**
Climbazole	6	2	32.2	14.4	8.8	5.2	1.5	0.5	-0.1	2.0	**32.2**
Ziprasidone	13	2	19.0	31.8	20.6	10.5	3.3	1.4	2.8	0.8	**31.8**
Anisotropine	8	2	31.5	22.4	12.4	9.4	4.2	4.2	1.7	2.9	**31.5**
Ifenprodil	37	1	4.6	15.6	24.8	31.2	31.2	29.4	23.0	13.6	**31.2**
Ro 8-4304	42	2	31.1	10.9	-0.1	0.7	-1.2	-1.8	-0.6	-0.1	**31.1**
U-101958	23	2	29.2	20.7	11.6	6.6	4.2	0.1	1.0	0.0	**29.2**
Amorolfine	50	2	10.3	19.6	22.5	22.8	25.9	26.9	22.9	22.9	**26.9**
JNJ-40418677	10	2	24.0	6.0	5.6	-0.2	2.5	-0.2	0.8	2.0	**24.0**
SANT-1	54	1	20.9	19.7	11.2	11.7	4.5	5.0	3.2	4.7	**20.9**
Q-VD-Oph & salubrinal	Positive control		20.8								**20.8**
m-Iodobenzylguanidine	18	2	19.5	20.3	13.7	14.5	9.2	3.6	6.1	1.7	**20.3**
Luliconazole	53	2	20.1	10.7	4.5	3.8	2.6	0.8	-0.2	-1.1	**20.1**
Mestranol	59	2	18.2	11.7	7.1	5.3	4.1	4.5	1.5	0.9	**18.2**
Ondansetron	44	2	18.2	7.6	12.5	7.9	3.8	4.1	2.5	4.5	**18.2**
Dofetilide	60	2	17.6	6.8	6.3	0.3	0.4	3.8	-0.5	5.8	**17.6**
Megestrol	33	2	17.2	5.9	2.2	2.4	1.6	3.6	2.5	3.6	**17.2**
Vitamin D2	16	1	17.1	12.4	5.9	1.5	0.8	4.2	3.0	2.2	**17.1**
Clobetasol	43	1	17.0	10.9	16.5	8.4	3.7	8.6	2.4	3.3	**17.0**
Halobetasol	11	2	12.9	10.3	12.8	15.7	10.3	4.0	13.2	7.8	**15.7**
Idebenone	63	1	13.6	9.0	3.3	1.7	2.1	2.4	-0.5	0.7	**13.6**
Oxybutynin	30	2	13.2	10.7	11.6	7.3	6.2	2.1	2.8	1.1	**13.2**
Budesonide	3	1	12.2	10.0	5.4	5.0	6.3	6.1	6.1	5.3	**12.2**
Wnt-C59	28	1	10.8	3.4	1.4	2.1	2.3	1.7	-0.2	-0.2	**10.8**
Palmatine	61	1	10.5	6.2	7.4	6.7	3.6	1.7	3.0	3.2	**10.5**
Dexlansoprazole	14	2	10.4	6.7	4.3	2.6	-0.1	0.9	2.4	0.1	**10.4**
Malotilate	24	1	9.8	2.0	2.5	0.5	-0.6	1.3	0.8	0.1	**9.8**
GW3965	21	2	9.4	8.2	5.1	1.5	2.6	0.4	0.8	-0.2	**9.4**
Mosapride	9	2	9.2	2.3	6.6	5.0	1.8	1.1	3.0	1.1	**9.2**
SB216763	34	2	0.5	5.0	8.5	4.1	2.9	1.5	2.9	1.9	**8.5**
Butenafine	49	1	8.1	5.6	2.6	2.5	1.1	1.4	-1.0	0.6	**8.1**
Betamethasone	36	2	7.9	4.5	2.4	5.9	2.9	3.0	2.6	3.3	**7.9**
Diphemanil	35	1	7.9	2.9	2.2	0.5	-1.4	1.0	0.2	-0.2	**7.9**
Enzalutamide	12	1	7.8	5.7	4.7	5.4	4.5	2.5	4.3	4.9	**7.8**

Q-VD-OPh	4	2	7.5	6.1	-3.4	1.6	0.4	0.1	-0.1	-0.6	**7.5**
AGI-6780	55	1	6.2	3.6	2.5	0.7	1.7	-0.1	1.0	-0.6	**6.2**
Isoconazole	17	2	25.9	18.2	14.0	9.9	10.4	5.9	4.2	4.1	**6.2**
AZD7545	52	2	6.0	3.7	2.1	4.4	0.6	-0.2	0.1	2.9	**6.0**
Salubrinal	57	1	3.3	5.0	6.0	5.2	4.8	2.3	3.2	0.4	**6.0**
Emricasan	40	1	5.5	-1.7	-0.4	2.0	3.3	0.9	1.2	0.0	**5.5**
OSI-930	46	1	-1.4	5.0	-0.5	1.7	1.8	-0.1	-1.4	0.5	**5.0**
Tubastatin A	47	1	4.9	1.5	0.9	3.4	5.0	3.7	2.1	0.3	**5.0**
IU1	36	1	4.7	2.2	1.0	1.7	-0.2	0.5	-1.3	-0.4	**4.7**
Progesterone	27	1	4.5	1.8	1.5	1.1	1.6	0.0	-0.1	-0.5	**4.5**
Pyrimethamine	5	1	2.8	4.2	1.6	3.4	3.8	2.3	4.3	3.0	**4.3**
Ouabain	20	1	0.0	1.6	1.1	3.0	-0.3	0.0	0.7	4.1	**4.1**
Allylthiourea	48	0									
Biperiden	39	0									
Cilostazol	64	0									
Cyproterone	31	0									
Fluorometholone	41	0									
Lubiprostone	26	0									
Praziquantel	19	0									
Sorafenib	58	0									
Ticlopidine	56	0									
ZM 336372	62	0									

Table S5. Hit confirmation and lead compound selection. Related to Figure 4
64 compound hits from primary (>3000) bioactive screen were tested in two additional iPSC-derived *jimpy* OPC lines at 10µM, single replicate per cell line. Hits that met the primary screening hit selection criterion in at least one cell line highlighted in black and considered confirmed compounds. Compounds that failed confirmation testing are highlighted in red. Confirmed hits were tested in the iPSC-derived *jimpy* OPC line used in the primary screen over an eight-point dose response (from 10µM to 78nM), with a single replicate well per compound dose. Values indicate the number of SDs above the mean of the negative control (DMSO vehicle) for quantified MBP+ oligodendrocytes. Values that met the MBP+ oligodendrocyte primary screening criterion are indicated by green. Compounds were then rank ordered based on their maximal responses, highlighting the lead compound Ro 25-6981 (indicated in grey).

Compound Name	10uM	5uM	2.5uM	1.25 uM	625 nM	313 nM	156 nM	78nM	39nM	20nM
Eliprodil	1.3	15.8	9.3	1.0	0.7	-0.2	0.0	-0.5	0.1	-0.3
Ifenprodil	28.2	5.3	18.4	10.7	6.8	0.7	0.6	0.7	0.0	0.3
Lubeluzole	2.0	-0.5	-0.6	0.4	-0.3	-0.5	0.1	0.1	0.1	-0.2
Rislenemdaz	-0.3	0.0	1.5	1.5	-0.3	-0.9	-0.2	-0.6	0.4	0.0
Ro 8-4304	3.8	0.0	-0.2	1.2	0.0	-0.9	-0.3	-0.8	0.1	0.1
Ro 25-6981	22.9	40.4	34.0	14.2	3.3	0.7	-0.2	-0.8	-0.2	-0.3
TCS 46b	22.9	6.4	0.6	-0.5	-0.8	-0.5	-0.2	-0.9	0.0	-0.5
TCN 237	2.3	-0.6	0.4	0.0	0.3	0.6	0.1	0.9	0.6	-0.2
(±)-2-Amino-3-phosphonopropionic acid	1.8	0.7	1.2	-0.5	0.0	0.0	0.6	2.4	0.7	2.3
(±)-2-Amino-4-phosphonobutyric acid	2.6	1.3	0.9	1.8	0.6	-0.3	0.0	-0.6	-0.2	-0.5
(±)-2-Amino-5-phosphonopentanoic acid	-0.5	1.6	0.4	0.9	2.0	-0.3	-0.6	1.5	0.0	-0.3
D(-)-2-Amino-5-phosphonopentanoic acid	0.7	0.0	1.2	0.1	0.9	0.1	-0.5	-0.3	-0.5	-0.5
Arcaine	1.0	0.7	0.0	0.1	-0.2	-0.5	-0.3	-0.3	0.0	-0.5
CNS-1102	-0.9	-0.6	-0.6	-0.2	-0.2	-0.5	-0.6	-0.8	-0.3	0.1
(±)-CPP	0.6	0.3	-0.2	1.8	0.4	1.3	0.3	1.0	-0.3	0.4
Dextromethorphan	-0.5	-0.2	-0.6	-0.6	-0.3	-0.3	0.0	-0.3	-0.3	-0.5
5,7-Dichlorokynurenic acid	-0.5	1.2	0.4	0.0	0.4	0.7	0.1	0.0	-0.5	0.3
5-Fluoroindole-2-carboxylic acid	0.3	-0.6	0.1	1.2	-0.2	0.3	1.0	-0.6	0.1	-0.5
Flupirtine	0.6	-0.2	0.9	0.3	-0.2	-0.3	0.1	-0.3	-0.6	-0.2
(±)-HA-966	-0.5	-0.3	-0.5	0.1	1.0	0.3	-0.3	-0.5	-0.9	-0.2
L-701,324	-0.5	0.4	0.4	1.3	1.0	-0.2	-0.2	-0.5	0.3	-0.5
Kynurenic acid	0.4	0.9	0.0	0.1	0.7	0.0	0.0	-0.2	1.0	0.4
Memantine	-0.2	-0.6	-0.3	0.3	0.0	0.6	2.3	0.0	1.2	0.0
MK-801	-0.9	0.9	2.3	-0.3	0.3	1.5	2.0	0.1	1.3	0.7
O-Phospho-L-serine	0.0	0.1	0.3	0.3	0.7	0.6	0.4	0.6	-0.2	1.6

Pentamidine	-0.9	-0.8	1.0	0.0	-0.6	-0.2	-0.9	0.0	0.7	-0.3
Quinolinic acid	1.3	-0.2	0.0	-0.5	0.4	0.1	0.4	0.0	0.7	-0.6
Spermidine	2.6	0.9	0.7	0.7	0.0	0.4	1.5	0.3	1.2	-0.2
Spermine	1.5	0.7	1.3	0.6	0.1	-0.2	2.0	0.3	0.1	0.6

Table S6. 10-point dose testing of 29 NMDA modulators on differentiating *jimpy* OPCs. Related to Figure 5
Effects of 29 NMDA receptor modulators (from 10µM to 19.5nM) on *jimpy* iPSC-derived OPCs after 3 days of oligodendrocyte differentiation. n=1 replicate well per compound dose. Values indicate the number of SDs above the negative control (DMSO vehicle control) mean for MBP+ oligodendrocytes. Values that met the primary screening hit selection criterion for the MBP+ cell metric are highlighted in green. All other values highlighted in red. NR2B-NMDA receptor-selective antagonists highlighted in grey.

Supplemental Experimental Procedures

Mice

All animal work was sanctioned by Case Western Reserve University's Institutional Animal Care and Use Committee (IACUC). Wild-type (B6CBACa/J A^{w-J}/A; 001201, Jackson Laboratory) and *jimpy* mutant (B6CBACa-A^{w-J}/*A*-*Plp1*^{jp} *Eda*^{Ta}/J; 000287, Jackson Laboratory) mice were housed and bred at Case Western Reserve University in a temperature-controlled environment with a 12-hour light / 12-hour dark cycle. Mice were provided a standard diet and water, *ad libitum*. Genotyping was performed by a quantitative PCR assay (Transnetyx) or Sanger sequencing using the forward and reserve primers AACGCAAAGCAGCACATTTCA and AGTGCAGCTCTGGGGTTAAT, respectively.

Immunochemistry and staining

Samples were fixed in 4% paraformaldehyde (PFA; 15710, Electron Microscopy Sciences), permeabilized, and blocked with 10% normal donkey serum (NDS; 017-000-121, Jackson Labs). The following primary antibodies used: rabbit anti-GFAP (1:5000; Z033429-2, Dako), rat anti-GFAP (1:1000; 13-0300, Thermo Fisher), mouse anti-MBP (1:500; 808401, Biolegend), rat anti-MBP (1:100; ab7349, Abcam), rabbit anti-MyRF antibody (1:1000; kindly provided by Dr. Michael Wegner), rabbit anti-Nanog (1:500; AB21624, Abcam), mouse anti-O4 (1:10; Lerner Research Institute Hybridoma Core), mouse anti-Oct3/4 (1:500; SC-5279, Santa Cruz), rabbit anti-Olig2 (1:1000; 13999-1-AP, ProteinTech), mouse anti-O4 (1:10; Lerner Research Institute Hybridoma Core), rat anti-PLP (1:500; Lerner Research Institute Hybridoma Core), goat anti-Sox10 (1:100; AF2864, R&D Systems), and rat anti-PLP (1:500; Lerner Research Institute Hybridoma Core). For secondary immunostaining Alex Fluor antibodies (ThermoFisher) were used at 1:500. To identify nuclei cells were stained with 100ng/ml DAPI (D8417, Sigma).

Tissue and cell culture

All cell and tissue cultures were maintained at 37° C with 5% CO2 in a humidified chamber. All OPC, oligodendrocyte, and astrocyte cultures were maintained on plates with coated with 100 µg/mL poly(L-ornithine) (P3655, Sigma), followed by 10 µg/ml laminin (L2020, Sigma). Plates were also purchased with a poly(D-lysine) which substituted for poly(L-ornithine).

Imaging and quantification of *in vitro* cultures

Unless otherwise noted immunochemistry images were captured using the Operetta® High Content Imaging and Analysis system (PerkinElmer) and Harmony® software (PerkinElmer). Images were analyzed and quantified using Columbus™ software (PerkinElmer) and a custom Acapella® (PerkinElmer) script. All phase images were captured on a Leica DM IL LED inverted microscope.

Isolation of tail-tip fibroblasts

Tail tips of approximately two millimeters were obtained from postnatal day nine *jimpy* mutant mice (B6CBACa-A^{w-J}/*A*-*Plp1*^{jp} *Eda*^{Ta}/J; Jackson Laboratory) as well as wild-type (B6CBACa-A^{w-J}; Jackson Laboratory) littermates. Tissue was bisected, placed on Nunclon-Δ 12-well plates (150628, ThermoFisher), and covered with a circular glass coverslip (12-545-102; Fisher Scientific) to maintain tissue contact with the plate and enable fibroblast outgrowth. Tail-tip fibroblasts were cultured in 'fibroblast medium' consisting of DMEM (11960069, ThermoFisher) with 10% fetal bovine serum (FBS; 16000044, ThermoFisher), 1x non-essential amino acids (11140050, ThermoFisher), 1x Glutamax (35050061, ThermoFisher), and 0.1 mM 2-mercaptoethanol (M3148, Sigma Aldrich) supplemented with 100U/mL penicillin-streptomycin (15070-063, ThermoFisher). Medium was changed every third day.

TTF reprograming and iPSC generation

TTFs were lifted with Accutase (AT104, Innovative Cell Technologies) and were seeded at 1.3-2.0x10^4 cells/cm^2 on Nunclon-Δ dishes in fibroblast medium and were allowed to equilibrate overnight. The following day medium was removed and replaced with an equal volume of pHAGE2-TetOminiCMV-STEMCCA-W-loxp (Somers et al., 2010; Sommer et al., 2009) lentivirus encoding a floxed, doxycycline-inducible polycistronic Oct4, Sox2, Klf4, and c-Myc construct (Takahashi et al., 2007; Welstead et al., 2008) and pLVX-Tet-On-Puro (632162, Clontech) lentivirus supplemented with 8µg/mL polybrene (107689, Sigma). Lentivirus was prepared using the *Lenti*-X Packaging Single *Shots (631275, Clontech) according to manufacturer's instructions*. Three hours later lentivirus medium was removed and replaced with fibroblast medium supplemented with 2 µg/ml doxycycline (631311, Clontech). The following day media was removed and replaced with an equal volume of pHAGE2-TetOminiCMV-STEMCCA-W-loxp and pLVX-Tet-On-Puro lentivirus supplemented with 8µg/mL polybrene. Three hours later lentivirus media was

removed and replaced with fibroblast medium supplemented with 2 μg/ml doxycycline and 10^3 units/ml LIF. After four days medium was changed with fibroblast medium supplemented with 2 μg/ml doxycycline and 10^3 units/ml LIF. After expansion tail-tip fibroblasts were lifted using Accutase and seeded at 6.25-12.5x10^4 cells/cm^2 on Nunclon-Δ plates, atop a feeder layer of irradiated mouse embryonic fibroblasts (iMEFs; produced in-house) previously plated at 3.3x10^4 cells/cm^2 on 0.1% gelatin (1890, Sigma) coated Nunclon-Δ plates in "pluripotency medium" consisting of Knockout DMEM (10829-018, ThermoFisher), 5% FBS, 15% knockout replacement serum (10828028, ThermoFisher), 1x Glutamax, 1x nonessential amino acids, 0.1 mM 2-mercaptoethanol, and 10^3 units/ml LIF (LIF; ESG1107, EMD Millipore). Medium was changed every day until iPSC colonies began to emerge. Individual colonies were picked and dissociated in 0.25% Trypsin-EDTA (25200056, ThermoFisher) and were individually plated in single wells of Nunclon-Δ 12-well plates, atop an iMEF feeder layer. iPSCs were expanded in pluripotency medium supplemented with doxycycline and medium was changed every day. Three individual iPSC clones per genotype were expanded and cryopreserved for future use (wild-type line identifiers = i.wt-1.0, i.wt-1.3, and i.wt-1.4 and *jimpy* lines identifiers = i.jp-1.4, i.jp-1.5, and i.jp-1.6). G-banded chromosomal analysis was performed on all iPSC lines at the seventh passage after derivation (Cell Line Genetics; Madison, WI). Approximately twenty cells were chosen per line and chromosomes were analyzed in metaphase spreads. Wild-type or *jimpy* genotypes were verified by Sanger sequencing.

OPC derivation from iPSCs

iPSCs were differentiated to OPCs as previously described (Najm et al., 2011, Lager et al., 2018). In brief, iPSC were isolated from their iMEF feeder layer using 1.5mg/mL collagenase type IV (17104019, ThermoFisher) and dissociated with either 0.25% Typsin-EDTA or Accutase and seeded at 7.8x10^4 cells/cm^2 on Costar Ultra-Low attachment 6-well plates (3471, Corning). Cultures were then directed through a 9-day differentiation process to generate "unenriched OPCs". Unenriched OPCs were further purified over three passages using OPC-selective medium. OPCs were maintained in "OPC medium" consisting of DMEM/F12 (11320082, ThermoFisher), 1x N2 supplement (AR009, R&D Systems), 1x B-27 without vitamin A supplement (12587-010, ThermoFisher), and 1x Glutamax (collectively "N2B27 medium"), supplemented with 20 ng/mL fibroblast growth factor 2 (FGF2; 233-FB, R&D Systems) and 20 ng/mL platelet-derived growth factor-AA (PDGF-AA; 221-AA, R&D Systems). Medium was changed every other day. The expected genotype for each wild-type or *jimpy* OPC line was confirmed by Sanger sequencing.

Characterization and purity assessment of iPSC-derived OPCs

For characterization of OPCs from all wild-type and *jimpy* iPSC lines, OPCs were seeded at 2.6 x10^5 cells/cm^2 in quadruplicate wells on Nunclon-Δ plates. Actively growing OPC cultures were fixed with 4% PFA and OPCs were immunostained using goat anti-Sox10, and rabbit anti-Olig2, followed by counterstaining with DAPI. 20 fields were captured per well and the total number of DAPI+, Sox10+, and Olig2+ cells were used to quantify the percentage of Olig2 and Sox10 double or single positive OPCs for each cell line.

Oligodendrocyte assessment after oligodendrocyte differentiation

Cultures of all wild-type and *jimpy* iPSC-derived OPCs (n=3 independent cell lines per genotype) were harvested in parallel using Accutase and seeded at 30,000 cells per well (n=4 wells per cell line) on 96-well Nunclon-Δ plates in "oligodendrocyte differentiation medium" which consisted of N2B27 medium supplemented with 100ng/mL noggin (3344-NG, R&D Systems), 10ng/mL neurotrophin-3 (NT-3; 267-N3, R&D Systems), 50 μM cAMP (D0260, Sigma), 100ng/mL insulin-like growth factor-1 (IGF-1; 291-G1, R&D Systems) NT-3, and 40ng/mL triiodothyronine (thyroid hormone; T-6397, Sigma). At 3 day post-plating medium was removed and cells were fixed with 4% PFA. Cells were immunostained using rat anti-MBP, followed by counterstaining with DAPI. 8 fields were captured per well and the total number of MBP+ cells were quantified for each cell line. A two-way, unpaired t-test was performed using Graphpad Prism software to compare statistical differences between genotypes.

Temporal dynamics of OPC differentiation to oligodendrocytes

Cultures of wild-type and *jimpy* iPSC-derived OPCs (from iPSC lines i.wt-1.0 and i.jp-1.6, respectively) were harvested in parallel using Accutase and seeded at 30,000 cells per well in 96-well poly-D-lysine Viewplates (6005710, PerkinElmer) in oligodendrocyte differentiation medium (n=6 replicate wells per genotype). At 2hr (plating control), 1 day, 2 day, and 3 day post-plating medium was removed and cells were live stained with mouse anti-O4 followed by fixation with 4% PFA. Cells were then immunostained using rat anti-MBP, followed by counterstaining with DAPI. 10 fields were captured per well and the total number of MBP+ cells were quantified for each cell line. To control for differences in cell plating, cell counts were normalized to the 2hr plating control.

Live-cell imaging of OPC differentiation

Parallel cultures of wild-type and *jimpy* iPSC-derived OPCs (from iPSC lines i.wt-1.0 and i.jp-1.6, respectively) were harvested in parallel using Accutase and seeded at $3.6x10^4$ cells/cm^2 in oligodendrocyte differentiation medium on a Nunclon-Δ plate. This plate was then positioned in an environmental chamber with temperature, humidity, and CO_2 control, and regions of interest were marked manually using Leica Application Suite X software. Phase images were automatically captured every 15 minutes during 3 days of oligodendrocyte differentiation using a DMI6000 microscope (Leica). A single image series for each genotype was stitched together to generate a movie using Leica Application Suite X software, and each movie was processed for background correction and aligned using Image Pro Premier 9 software (Media Cybernetics). Wild-type and *jimpy* time-lapse movies were placed side-by-side in iMovie (version 10.1.5).

RNA-seq and Gene Ontology Enrichment Analysis

Parallel cultures of OPCs from wild-type and *jimpy* iPSC lines (n=3 independent cells lines, per genotype) were seeded at $2.3x10^4$ cells/cm^2 in oligodendrocyte differentiation or OPC medium on Nunclon-Δ flasks. One day later cells were homogenized in TRIzol (15596026, ThermoFisher). Samples were chloroform extracted and phase separated. RNA was isolated using the miRNeasy Mini kit (217004, Qiagen). Sequencing libraries were generated using the TruSeq Stranded Total RNA kit (20020596, Illumina) after rRNA depletion with RiboZero Gold (MRZG126, Illumina). 100bp paired-end sequencing was performed on an Illumina HiSeq 2500 at the Case Western Reserve University School of Medicine Genomics Core Facility. Reads were aligned to the iGenomes mm9 genome using default settings in Tophat v2.0.6 (Trapnell et al., 2009), without providing a reference transcriptome. Abundances of transcripts in the iGenomes mm9 RefSeq transcriptome were calculated using Cufflinks v2.0.2 (Trapnell et al., 2010) to generate data normalized FPKM values.

Expressed genes were used for gene ontology analysis. All biological replicates for each condition were averaged and genes with a FPKM >0.25 in at least one condition were considered expressed. Expressed genes were tabled by converting FPKM values <0.25 to 0.25. Day one oligodendrocyte differentiation conditions were normalized to their respective OPC condition. Normalized expression values were then compared between genotypes: [(*jimpy* D1 oligodendrocyte/*jimpy* OPC)]/[(wild-type D1 oligodendrocyte/wild-type OPC)]. Normalized, differential expression values were evaluated using Gene Set Enrichment Analysis software (Subramanian et al., 2005), generating gene set enrichment scores using Hallmark, C2.all, and C5.all Molecular Signatures Database (MSigDB) datasets. Gene sets with an FDR of <0.05 were processed with Cytoscape v3.2.1 (Shannon et al., 2003) and the Enrichment Map plug-in (Merico et al., 2010) to generate pathway networks. *Plp1* expression in OPCs and day 1 oligodendrocyte differentiation samples were assessed using data normalized FPKM values. Each day 1 oligodendrocyte differentiation sample was further normalized by dividing by the FPKM value of its respective OPC line.

scRNA-seq by Drop-Seq and GSEA

Wild-type and *jimpy* iPSC-derived OPCs (from iPSC lines i.wt-1.0 and i.jp-1.6, respectively) were harvested in parallel using Accutase and seeded at $6.6x10^4$ cells/cm^2 in oligodendrocyte differentiation medium on Nunclon-Δ flasks. One day later cells were harvested using Accutase and resuspended in N2B27 containing 0.1% bovine serum albumin (15260037, ThermoFisher) and immediately processed for Drop-Seq as previously described (Macosko et al., 2015). In brief, three individual syringes containing cell suspension at 200,000 cells/mL barcoded mRNA capture beads (MACOSKO-2011-10, ChemGenes) in lysis buffer (prepared according to instructions in Drop-Seq protocol (Macosko et al., 2015) at 200,000 beads/mL, and QX200 droplet generation oil (186-4006, Bio-Rad), respectively, were placed in a Legato™ 100 pump (788100, Analytical West). Each output was connected to a custom-made PDMS co-flow microfluidic droplet generation device (Nanoshift) under microscope supervision. Cell and bead flow was set to 4,000uL/hr, and oil flow was set to 15,000uL/hr during droplet generation. These settings were optimized to allow at most one bead per one cell. Droplets were collected and then broken to release beads. First strand cDNA synthesis with incorporation of a PCR handle was performed on the collected beads using the Maxima H Minus Reverse Transcriptase (EP0752, ThermoFisher). The resulting full-length cDNA library was prepared for sequencing as described (Macosko et al., 2015) and sequenced on an Illumina HiSeq 2500 at the Case Western Reserve University School of Medicine Genomics Core Facility. Raw reads were processed as described (Macosko et al., 2015). The resulting reads were aligned to iGenomes mm9 RefSeq transcriptome using STAR v.2.5 (Dobin et al., 2013) with default settings. Mapped reads were filtered to contain only uniquely mapped, exonic reads. Reads were then grouped by cell barcode, generating a digital UMI-count matrix quantifying the number of unique transcripts per gene. We confirmed that wild-type and *jimpy* cells had similar profiles of sequenced transcripts per cell (Supplementary Figure 3a).

To perform unsupervised clustering we first identified 500 genes with high inter-cell expression variability. To identify this gene set we employed cells with at least 1000 total transcripts. We summed all transcripts per gene and ordered genes by expression level (highest to lowest). The top 10,000 genes were divided into 10 bins by expression level, with each bin containing 1,000 genes. Coefficient of variation (CV) was calculated within each bin for each gene. We selected the 50 genes per bin with the highest variability and combined them into a final list of 500 highly-variable genes.

Basic clustering was executed using the Seurat package (Macosko et al., 2015; Satija et al., 2015) using default filtering parameters. Next we performed principle component analysis (PCA) PCA using our 500 highly-variable gene list (see above). With PC dimension from PC1 to PC10, cells were embedded in a K-nearest neighbor (KNN) graph. Smart local moving algorithm (SLM) was applied for grouping cells into communities. tSNE was performed with PC1 to PC10 as input and cell clusters were viewed in tSNE space.

To define cell identity, we used the Seurat *FindMarker* function to identify genes with high expression and cluster exclusivity. Expression of specific markers (*Top2a* and *Ccnd1* for OPCs, *Apoe* and *Slc1a2* for nascent astrocytes, *Mbp* and *Enpp6* for nascent oligodendrocytes) was used to apply cell-type classifications to the different clusters. For differential expression analysis we extracted the expression matrix for wild-type and *jimpy* OPCs, nascent oligodendrocytes, and nascent astrocytes.

We employed a generalized linear model using the R package *MASS* by command ***glm.nb*** as shown below:

$$\mathbf{\log(UMI) = \beta_0 + \beta_1 C + \beta_2 D + \log(sf)}$$

where **log(UMI)** is assumed to have a negative binomial distribution

UMI represents the number of transcripts
β_0 is the regression intercept
β_1 is the parameter for C
C represents the cell type, a factor data type in R to describe the cell type for a given cell
D represents the donor, a factor data type in R to describe the origin of a given cell
sf is the size factor. This is calculated by command ***computeSumFactors*** in Bioconductor package *scran* sf to describe the size of a given cell, as previously described (Lun et al., 2016)

The response variable is UMI, which is regressed on two predictors: C; D. The key factor is C, which models the variance between cell types for a given gene. D and sf are used to regress out the influence of different individual and the difference in cell origin and cell library size, respectively. With regards to the estimation for sf, since the dropout is zero in single cell data, we made use of deconvolution strategy to normalize and calculate cell library size using the command ***computeSumFactors*** in Bioconductor package ***scran* sf** as previously described (Lun et al., 2016). We modeled all genes across all cell types iteratively and used the Wald test to calculate per gene p-values. We adjusted each p-value with the Bioconductor package ***qvalue*** to generate estimated q-values.

Per gene fold-change differences between wild-type and *jimpy* cell types (OPCs, nascent oligodendrocytes, and nascent astrocytes) were calculated in two ways: 1) Mean fold change and 2) fold change as a percentage of expression. We filtered genes using the q-value (<0.05) and ranked them by the fold change. For enrichment analysis we selected genes with a p value <0.01 and fold change >1.2 or <0.8 to capture the top up or down regulated genes, respectively. Finally we performed GSEA using MSigDB datasets to show pathway enrichment in *jimpy* nascent oligodendrocytes, OPCs, or nascent astrocytes relative to wild-type nascent oligodendrocytes, OPCs, or nascent astrocytes, respectively.

<u>Astrocyte differentiation and co-culture</u>
Wild-type and *jimpy* iPSC-derived OPCs (from iPSC lines i.wt-1.0 and i.jp-1.6, respectively) were harvested in parallel using Accutase and seeded at 10,000 OPCs per well in two 96-well Nunclon-Δ plates in 'astrocyte medium' which consisted of 1:1 neurobasal media (211030-049, ThermoFisher) and DMEM (11960-044, ThermoFisher) with 1mM sodium pyruvate (ThermoFisher Cat#: 11360-070), 1x Glutamax, 1x N2 supplement, and 30uM N-acetyl-cysteine (A8199, Sigma) supplemented with 10ng/ml of CNTF (557-NT, R&D Systems) and 50ng/ml of BMP4 (314-BP, R&D Systems) (Mabie et al., 1999; Scholze et al., 2014). Three days later one astrocyte differentiation plate

(astrocyte characterization plate) was fixed with 4% PFA and immunostained using rabbit anti-GFAP followed by counterstaining with DAPI (n=18 replicate wells per genotype). Medium was changed to N2B27 medium on the remaining astrocyte differentiation plate (astrocyte co-culture plate). After three days separate cultures of wild-type and *jimpy* iPSC-derived OPCs (from iPSC lines i.wt-1.0 and i.jp-1.6, respectively) were harvested in parallel using Accutase and seeded at 30,000 OPCs per well in oligodendrocyte differentiation medium on *jimpy* or wild-type iPSC-derived astrocytes, respectively (astrocyte co-culture plate), or on a 96-well Nunclon-Δ plate (oligodendrocyte differentiation control plate). Three days later astrocyte co-culture and oligodendrocyte differentiation control plates were fixed with 4% PFA. The oligodendrocyte differentiation control (n=8 replicate wells per genotype) and astrocyte co-culture (n=6 replicate wells per genotype) plates were immunostained using mouse anti-MBP followed by counterstaining with DAPI. 9 fields were captured per well and the total number of DAPI+ and GFAP+ cells were used to quantify the percentage of GFAP+ cells for the astrocyte characterization plate. The total number of DAPI+ and MBP+ cells were used to quantify the number of oligodendrocytes for the oligodendrocyte differentiation control and astrocyte co-culture plates.

Compound screening and assessment

384-well poly-D-lysine CellCarrier Ultra plates (6057500, PerkinElmer) were coated with 10 µg/ml laminin solution in N2B27 using a Biotek EL406 Microplate Washer Dispenser with a 5µL dispenser cassette (7170011, Biotek) and incubated at 37°C. Bioactive compound libraries dissolved in DMSO were accessed from Case Western Reserve University's Small Molecule Drug Development Core. Compounds were added to the CellCarrier Ultra plates with a 50nL pin head coupled to a Perkin Elmer JANUS® automation workstation. The following compounds were used for compound screening and assessment: Salubrinal (SML0951, Sigma), Q-VD-OPh (OPH001, R&D systems), emricasan (HY-10396, MedChem Express), rislenemdaz (HY-106441A, MedChem Express), TCN 237 (4072, Tocris Bioscience), TCS 46b (2782, Tocris Bioscience). For the >3000 bioactive compound primary screen or compound cherry-picking, two separate compound libraries were used (L1700, Selleck Chemicals and LO1280, Sigma). Additionally DMSO vehicle negative control and 10µM Q-VD-OPh (R&D Systems) plus 10µM salubrinal (Sigma) positive control were added to column 23 and column 24 of each primary screen plate. For cell plating, OPCs were harvested using Accutase and seeded at 15,000 cells per well using the Biotek EL406 Microplate Washer Dispenser with a 5µL dispenser cassette. All assays were performed using i.jp-1.6 or i.wt-1.0 OPCs unless otherwise indicated. Final medium consisted of oligodendrocyte differentiation medium and appropriate concentration of compound. After three days plates were fixed and immunostained with rat anti-MBP and goat anti-Sox10, and counterstained with DAPI using a Biotek EL406 Washer Dispenser outfitted with a 96-well aspiration manifold and a 5µL dispenser cassette. Plates were imaged on the Operetta® High Content Imaging and Analysis system (PerkinElmer). 5 fields were captured and the total number of cells and MBP+ oligodendrocytes were quantified using a robust Acapella® (PerkinElmer) script (Figure S5d-h). All staining, imaging, and analysis steps were performed simultaneously for each screen using identical procedures to reduce plate-to-plate variability.

Isolation and expansion of primary *jimpy* OPCs

Postnatal day two *jimpy* pups were cyro-anesthetized and euthanized by rapid decapitation and whole brains were isolated in ice cold DMEM/F12 with 50U penicillin-streptomycin. Cerebral cortex was dissected from surrounding tissue and meninges were removed. Tissue was transferred to tissue dissociation solution (from kit 130-095-929, Miltenyi Biotec) and incubated at 37° C for 10 minutes with gentle trituration every 5 minutes. Cell solution was filtered through a 70µm Corning Falcon™ cell strainer (08-771-2, ThermoFisher) and cells were seeded at 2.6-7.1 x 10^4 cells/cm^2 in OPC medium supplemented with 100U/mL penicillin-streptomycin, on Nunclon-Δ plate. Medium was changed every other day. At 80-95% confluence cells were passaged 1:2 with Accutase for one passage in OPC selection medium, followed by one additional passage in OPC medium. Medium was changed every other day. Primary cortical cultures were used immediately after passage 2.

NR2B-NMDA receptor assessment by qRT-PCR

Parallel cultures of OPCs from wild-type and *jimpy* iPSC lines (i.wt-1.0 and i.jp-1.6, respectively) were simultaneously harvested using Accutase and seeded at 5.2x10^4 with oligodendrocyte differentiation medium on Nunclon-Δ plates. Cultures were harvested at days 0, 1, 2, and 3 using TRIzol reagent. Positive control samples consisting of cerebral cortex from age-matched postnatal week 3 wild-type and *jimpy* mice were collected using TRIzol reagent. All samples were chloroform extraction and phase separated in parallel. RNA was prepared with the RNeasy Mini kit (74104, Qiagen) according to the manufacturer's instructions. cDNA was generated using 1µg of total RNA per reaction using the iScript cDNA synthesis kit (1708890, Bio-Rad) according to the manufacturer's instructions. Quantitative RT-PCR was performed using 2.5ng RNA per reaction and the following pre-designed Taqman gene expression assays

(ThermoFisher): *Grin2b* (Mm00433820_m1) and *Actb* (Mm00607939_s1). *Actb* was used as an endogenous control to normalize expression values. n=3 technical replicates for the i.wt-1.0 day 0 sample and n=4 technical replicates for all other samples were analyzed on the Applied Biosystems 7300 Real-time PCR system. Samples were normalized to the wild-type cerebral cortical positive control.

Ro 25-6981 mechanistic studies by qRT-PCR

Parallel cultures of OPCs from wild-type and *jimpy* iPSC lines (i.wt-1.0 and i.jp-1.6, respectively) were simultaneously harvested using Accutase and seeded at 7.8x10^4 cells/cm^2 in oligodendrocyte differentiation or OPC medium containing DMSO vehicle on Nunclon-Δ plates. Additionally wild-type and *jimpy* OPCs were plated in oligodendrocyte differentiation medium containing 5µM Ro 25-6981 (R7150, Sigma) or 10µM salubrinal (Sigma). One day later RNA was harvested from all conditions using TRIzol reagent followed by chloroform extraction and phase separation. RNA and cDNA were prepared as described above. Quantitative RT-PCR was performed using 10ng RNA per reaction the following pre-designed Taqman gene expression assays (ThermoFisher): *Plp1* (Mm01297210_m1), *Ddit3* (Mm01135937_g1), *Atf4* (Mm00515325_g1), *Dnajb9* (Mm01622956_s1), *s-Xbp1* (Mm03464497_s1), *Hspa5* (Mm00517691_m1), and *Atf6* (Mm01295319_m1). *Actb* was used as an endogenous control to normalize expression values. n=3 technical replicates per *Atf6* sample and n=4 technical replicates for all other samples were analyzed on the Applied Biosystems 7300 Real-time PCR system. To assess for differentiation-specific expression changes all samples in oligodendrocyte differentiation medium were normalized to their respective OPC control lines.

Treatment of Human PMD Oligocortical Spheroids with Ro 25-6981

Human PMD cortical spheroids were generated using PMD *PLP1*$^{c.254T>G}$ and gene-corrected (isogenic control) iPSCs according to an established cortical spheroid protocol (Pasca et al., 2015) with modifications described in (Madhavan et al., 2018). From days 60-90 medium was supplemented with DMSO vehicle or 1 µM Ro 25-6981. Ro 25-6981 dose was chosen based its *in vitro* efficacy in *jimpy* cultures while also minimizing the possibility of potential toxicities from long-term culturing with the compound. At day 90 four spheroids (cultured in independent wells) per treatment were collected, fixed in 4% PFA for 12 minutes, cryoprotected in 30% sucrose, embedded in Tissue-Tek® Optimum Cutting Temperature compound (O.C.T.; 25608-930, VWR) and cryosectioned to produce 10 µm sections. Sections were immunostained using rat anti-PLP and rabbit anti-MyRF antibody, and counterstained with DAPI. Images were captured using a Leica SP8 Gated STED Microscope or a Leica DMi8 inverted microscope (for cell counts). Image analysis and cell counting was accomplished using Adobe Photoshop (Abobe Systems). The total number of DAPI+ and MyRF+ cells from equally sized and spaced fields per spheroid were counted and n=4 spheroids for each treatment condition were used to calculate the percentage of MyRF+ oligodendrocytes in each treatment group.

Treatment of postnatal *jimpy* pups with Ro 25-6981

From the onset of corpus callosal myelination at postnatal day 5 to day 14 jimpy pups were administered 1 mg/kg or 10mg/kg Ro 25-6981 (1594, R&D Systems), or saline vehicle control by intraperitoneal (i.p.) injection once a day. As an additional control wild-type male littermates were administered saline by i.p. injection once a day. Experimenters were blinded to the treatment being administered. At day 14 all injected mice were deeply anesthetized using isoflurane and transcardially perfused with PBS followed by fixation with 4% PFA. Tissue was cyroprotected with 30% sucrose, embedded in O.C.T. and cryosectioned at 20 µm thickness. Sections were immunostained using rabbit anti-MyRF and rat anti-MBP antibody. Images of the complete corpus callosum (medial, sagittal sections) were captured using a Leica SP8 Gated STED Microscope or a Leica DMi8 inverted microscope (for cell counts). Image analysis and cell counting of MyRF+ cells across the entire corpus callosum was accomplished using Adobe Photoshop, n=4 independent animals per treatment. One-way ANOVA with Holm-Sidak correction for multiple comparisons was performed using Graphpad Prism software to compare statistical differences between treatments.

Myelination assessment of Ro 25-6981 using synthetic microfibers

Mimetix® 384-well plates containing aligned 4 µm diameter poly-lactide fibers labeled with rhodamine-6G (AMS-TECL-015, AMSBio) were incubated in 70% ethanol. Next fibers were coated with 100 µg/mL poly(L-ornithine) followed by 10 µg/ml laminin. Wild-type and *jimpy* iPSC-derived OPCs (i.wt-1.0 and i.jp-1.6, respectively) were harvested using Accutase and, after removal of laminin solution, seeded at 15,000 OPCs per well in oligodendrocyte differentiation medium using a Biotek EL406 Microplate Washer Dispenser with a 5µL dispenser cassette (Biotek) and incubated at 37°C. Ro 25-6981 (R&D systems), as well as the additional modulators of *jimpy* pathology including salubrinal (Sigma) and Q-VD-OPh (R&D Systems), were all dissolved in DMSO. Compounds were added to the microfiber plates with a 50nL pin head coupled to a Perkin Elmer JANUS® automation workstation to achieve the

appropriate final concentration in each well. From days 0-3 medium was supplemented with 40ng/mL thyroid hormone to induce oligodendrocyte differentiation. On day 3 thyroid hormone was removed, and medium was subsequently changed every third day. Compounds or DMSO vehicle were added at each medium change. At day 3 and day 10 plates were fixed. All plates were immunostained with rat anti-MBP and counterstained with DAPI using a Biotek EL406 Washer Dispenser outfitted with a 96-well aspiration manifold and a 5μL dispenser cassette. Plates were imaged on the Operetta® High Content Imaging and Analysis system. A total of five fields were captured at 20x using Acapella® software, and images were analyzed using Harmony® software and Columbus™ software. Using this analysis software we developed an Acapella® script to quantify the total fiber area surrounded by MBP+ oligodendrocytes (Figure S7a-e). First, rhodamine signal was detected, quantified, and used as a mask in subsequent steps (Figure S7b,d). Next MBP signal was detected and a threshold was set to reduce false positive calls (Figure S7c,e). Finally the percent MBP+ coverage relative to total microfiber area was calculated. All staining, imaging, and analysis steps were performed simultaneously for each microfiber plate using identical procedures to reduce plate-to-plate variability.

Data Availability

All RNA-seq data have been deposited to the Gene Expression Omnibus (GEO) database under the accession number GSE111605.

Supplemental References

Dobin, A., Davis, C.A., Schlesinger, F., Drenkow, J., Zaleski, C., Jha, S., Batut, P., Chaisson, M., and Gingeras, T.R. (2013). STAR: ultrafast universal RNA-seq aligner. Bioinformatics *29*, 15-21.

Lun, A.T., Bach, K., and Marioni, J.C. (2016). Pooling across cells to normalize single-cell RNA sequencing data with many zero counts. Genome Biol *17*, 75.

Mabie, P.C., Mehler, M.F., and Kessler, J.A. (1999). Multiple roles of bone morphogenetic protein signaling in the regulation of cortical cell number and phenotype. The Journal of neuroscience : the official journal of the Society for Neuroscience *19*, 7077-7088.

Macosko, E.Z., Basu, A., Satija, R., Nemesh, J., Shekhar, K., Goldman, M., Tirosh, I., Bialas, A.R., Kamitaki, N., Martersteck, E.M.*, et al.* (2015). Highly Parallel Genome-wide Expression Profiling of Individual Cells Using Nanoliter Droplets. Cell *161*, 1202-1214.

Merico, D., Isserlin, R., Stueker, O., Emili, A., and Bader, G.D. (2010). Enrichment map: a network-based method for gene-set enrichment visualization and interpretation. PLoS One *5*, e13984.

Pasca, A.M., Sloan, S.A., Clarke, L.E., Tian, Y., Makinson, C.D., Huber, N., Kim, C.H., Park, J.Y., O'Rourke, N.A., Nguyen, K.D.*, et al.* (2015). Functional cortical neurons and astrocytes from human pluripotent stem cells in 3D culture. Nature methods *12*, 671-678.

Satija, R., Farrell, J.A., Gennert, D., Schier, A.F., and Regev, A. (2015). Spatial reconstruction of single-cell gene expression data. Nature biotechnology *33*, 495-502.

Scholze, A.R., Foo, L.C., Mulinyawe, S., and Barres, B.A. (2014). BMP signaling in astrocytes downregulates EGFR to modulate survival and maturation. PLoS One *9*, e110668.

Shannon, P., Markiel, A., Ozier, O., Baliga, N.S., Wang, J.T., Ramage, D., Amin, N., Schwikowski, B., and Ideker, T. (2003). Cytoscape: a software environment for integrated models of biomolecular interaction networks. Genome Res *13*, 2498-2504.

Subramanian, A., Tamayo, P., Mootha, V.K., Mukherjee, S., Ebert, B.L., Gillette, M.A., Paulovich, A., Pomeroy, S.L., Golub, T.R., Lander, E.S.*, et al.* (2005). Gene set enrichment analysis: a knowledge-based approach for interpreting genome-wide expression profiles. Proceedings of the National Academy of Sciences of the United States of America *102*, 15545-15550.

Takahashi, K., Tanabe, K., Ohnuki, M., Narita, M., Ichisaka, T., Tomoda, K., and Yamanaka, S. (2007). Induction of pluripotent stem cells from adult human fibroblasts by defined factors. Cell *131*, 861-872.

Trapnell, C., Pachter, L., and Salzberg, S.L. (2009). TopHat: discovering splice junctions with RNA-Seq. Bioinformatics *25*, 1105-1111.

Trapnell, C., Williams, B.A., Pertea, G., Mortazavi, A., Kwan, G., van Baren, M.J., Salzberg, S.L., Wold, B.J., and Pachter, L. (2010). Transcript assembly and quantification by RNA-Seq reveals unannotated transcripts and isoform switching during cell differentiation. Nature biotechnology *28*, 511-515.

Welstead, G.G., Brambrink, T., and Jaenisch, R. (2008). Generating iPS cells from MEFS through forced expression of Sox-2, Oct-4, c-Myc, and Klf4. J Vis Exp.
