# Supplementary material for: Chemical Screening Identifies Enhancers of Mutant Oligodendrocyte Survival and Unmasks a Distinct Pathological Phase in Pelizaeus-Merzbacher Disease
Source: Stem Cell Reports. 2018 Aug 23;11(3):711–26. doi: 10.1016/j.stemcr.2018.07.015 (PMC6135742; doi:10.1016/j.stemcr.2018.07.015)
Supplement: Document S2. Article plus Supplemental Information [file mmc7.pdf]

Stem Cell Reports
Article
ISSCR
OPEN ACCESS

# Chemical Screening Identifies Enhancers of Mutant Oligodendrocyte Survival and Unmasks a Distinct Pathological Phase in Pelizaeus-Merzbacher Disease

Matthew S. Elitt,[1] H. Elizabeth Shick,[1] Mayur Madhavan,[1] Kevin C. Allan,[1] Benjamin L.L. Clayton,[1] Chen Weng,[1] Tyler E. Miller,[1] Daniel C. Factor,[1] Lilianne Barbar,[1] Baraa S. Nawash,[1] Zachary S. Nevin,[1] Angela M. Lager,[1] Yan Li,[1] Fulai Jin,[1,3,4,5] Drew J. Adams,[1,5] and Paul J. Tesar[1,2,5,*]
[1]Department of Genetics and Genome Sciences, Case Western Reserve University School of Medicine, Cleveland, OH 44106, USA
[2]Department of Neurosciences, Case Western Reserve University School of Medicine, Cleveland, OH 44106, USA
[3]Department of Population and Quantitative Health Sciences, Case Western Reserve University School of Medicine, Cleveland, OH 44106, USA
[4]Department of Engineering and Computer Science, Case Western Reserve University School of Medicine, Cleveland, OH 44106, USA
[5]Case Comprehensive Cancer Center, Case Western Reserve University School of Medicine, Cleveland, OH 44106, USA
*Correspondence: paul.tesar@case.edu
https://doi.org/10.1016/j.stemcr.2018.07.015

## SUMMARY

Pelizaeus-Merzbacher disease (PMD) is a fatal X-linked disorder caused by loss of myelinating oligodendrocytes and consequent hypomyelination. The underlying cellular and molecular dysfunctions are not fully defined, but therapeutic enhancement of oligodendrocyte survival could restore functional myelination in patients. Here we generated pure, scalable quantities of induced pluripotent stem cell-derived oligodendrocyte progenitor cells (OPCs) from a severe mouse model of PMD, *Plp1*$^{jimpy}$. Temporal phenotypic and transcriptomic studies defined an early pathological window characterized by endoplasmic reticulum (ER) stress and cell death as OPCs exit their progenitor state. High-throughput phenotypic screening identified a compound, Ro 25–6981, which modulates the ER stress response and rescues mutant oligodendrocyte survival in *jimpy*, *in vitro* and *in vivo*, and in human PMD oligocortical spheroids. Surprisingly, increasing oligodendrocyte survival did not restore subsequent myelination, revealing a second pathological phase. Collectively, our work shows that PMD oligodendrocyte loss can be rescued pharmacologically and defines a need for multifactorial intervention to restore myelination.

## INTRODUCTION

Leukodystophies are a class of rare, heritable disorders characterized by a loss of central nervous system (CNS) myelin. Collectively these diseases impact approximately 1 in 7,500 live births, and are typified by extensive neurological impairment with reduced life expectancy (Bonkowsky et al., 2010; Parikh et al., 2015). While the causative mutations for these disorders are well established, a comprehensive understanding of the molecular and cellular phenotypes of each mutation remains elusive. Genotype-specific, molecular, and cellular phenotyping of oligodendrocytes—the myelinating glia of the CNS—during key pathological events would be invaluable for filling this deficiency and underpin future precision medicine initiatives.

We sought to establish the technology to systemically interrogate the archetypal, cell-autonomous leukodystrophy called Pelizaeus-Merzbacher disease (PMD) (MIM no. 312080) (Garbern, 2007; van der Knaap and Bugiani, 2017). PMD is a fatal X-linked disorder of the CNS caused by mutations in *proteolipid protein 1* (*PLP1*). PLP and its splice isoform DM20 are almost exclusively expressed in oligodendrocytes and oligodendrocyte progenitor cells (OPCs), respectively (Mallon et al., 2002). While the disease is thought to be mediated through mutant PLP-induced endoplasmic reticulum (ER) stress (Garbern, 2007), complete appreciation of the cellular and molecular pathology in PMD remains elusive, in part due to complexities in accessing or appropriately modeling the disease-affected oligodendrocyte lineage during critical developmental windows (Bradl et al., 1999; Cerghet et al., 2001; Duncan et al., 2011; Gow et al., 1998; Knapp et al., 1986, 1987; Southwood et al., 2002; Williams and Gard, 1997). Our group and others has recently begun to address these tractability issues using human PMD patient, induced pluripotent stem cell (iPSC)-based approaches (Nevin et al., 2017; Numasawa-Kuroiwa et al., 2014), but these cellular models are still constrained by multi-month differentiations and are confounded by contaminating cell types.

Our mouse pluripotent stem cell-based technology enables the rapid generation of high-purity, expandable OPCs that are capable of coordinated differentiation to oligodendrocytes *in vitro* over a defined 3-day period (Najm et al., 2011). This system provides precise developmental control to examine the oligodendrocyte lineage without artifact from contaminating cell types (Najm et al., 2011, 2015). Notably, these tools and similiar techniques have recently been coupled to high-throughput compound screening investigations and have identified novel therapeutics for autoimmune-mediated myelin disorders (Green et al., 2017; Helman et al., 2015; Hubler et al., 2018; Lee et al., 2013; Najm et al., 2015).

Here we extend this cellular technology to a well-established animal model for the severe, connatal form of


This is an open access article under the CC BY license (http://creativecommons.org/licenses/by/4.0/).

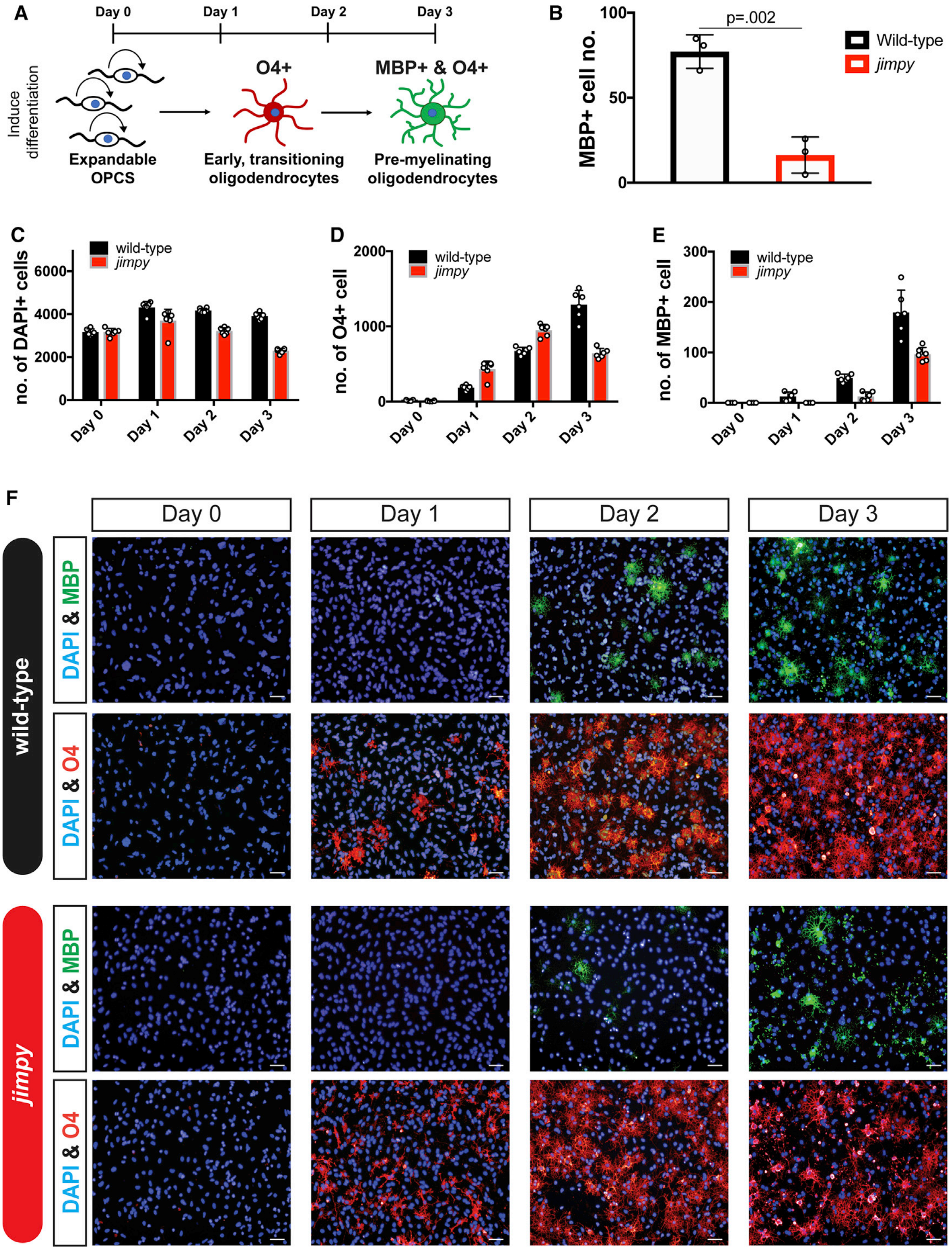


**Figure 1. Temporal Dissection of Cellular Phenotypes Reveals Cell Death Just as *Jimpy* OPCs Commit to an Oligodendrocyte Fate**

(A) Schematic summary of the experimental assay.

(B) Quantification of wild-type and *jimpy* MBP+ oligodendrocytes after oligodendrocyte differentiation. n = 3 distinct cell lines per genotype, with each replicate value (comprising a mean of n = 4 replicate wells per cell line) represented by a white circle. p values calculated using a two-way, unpaired t test between genotypes.

*(legend continued on next page)*

PMD called the "*jimpy*" (*Plp1*$^{jimpy}$) mouse (Duncan et al., 2011; Phillips, 1954; Yool et al., 2000), providing scalable access to the previously restricted PMD oligodendrocyte lineage. Using iPSC-derived *jimpy* OPCs, we first establish critical cellular and molecular deficits at key developmental stages during oligodendrocyte differentiation. We overcome this pathology using high-throughput chemical screening, identifying compounds capable of rescuing mutant oligodendrocyte survival in *jimpy* and human PMD cultures *in vitro*, and in *jimpy* mice *in vivo*. Surprisingly, these studies revealed that enhancing mutant oligodendrocyte survival does not, on its own, confer an expected restoration of myelin, unmasking a second stage of pathology and informing future phenotyping and therapeutic strategies for PMD.

## RESULTS

### *Jimpy* iPSC-Derived OPCs Reveal Early Differentiation Pathology

We established three independent iPSC lines from *jimpy* and isogenic wild-type littermate mice (see Experimental Procedures). iPSC lines exhibited typical iPSC morphology and expressed the canonical pluripotency markers Oct3/4 and Nanog (Figures S1A–S1D). Sanger sequencing confirmed *jimpy* mutation status (Figure S1E) and all iPSC lines displayed grossly normal karyotypes (Figure S1F). We directed all six iPSC lines to form high-purity OPCs (see Experimental Procedures) expressing the canonical OPC transcription factors Sox10 and Olig2 (Figures S2A–S2E), which had no overt morphological differences (Figure S2F).

Previous reports have documented cellular phenotypes at discrete stages during oligodendrogenesis in PMD cellular and animal models (Gow et al., 1998; Nevin et al., 2017; Numasawa-Kuroiwa et al., 2014; Readhead et al., 1994). We directed all iPSC-derived *jimpy* and wild-type control OPC lines toward an oligodendrocyte fate (Figure 1A) and noted a striking absence of MBP+ oligodendrocytes in all *jimpy* lines (Figure 1B). We further parsed this cellular defect with time-course immunocytochemistry. As expected, *jimpy* MBP+ oligodendrocytes failed to accumulate across all time points (Figures 1E and 1F). Surprisingly we noted substantial cell death well before MBP+ oligodendrocyte formation, within 1 day after inducing differentiation (Figures 1C and 1F), which we further confirmed by time-lapse imaging (Video S1). Intriguingly, *jimpy* OPCs showed increased propensity to acquire the late OPC/immature oligodendrocyte marker O4 early in the differentiation process (Figures 1D and 1F), which may reflect a feedback loop to promote stem cell exit in the context of severe cell loss during oligodendrocyte differentiation. Together these results precisely define an early developmental susceptibility period during which transitioning *jimpy* OPCs die as they exit their progenitor cell state.

### Transcriptome Profiling Reveals ER Stress in *Jimpy* Oligodendrocytes Immediately after the Onset of OPC Differentiation

We analyzed transcriptomic changes between *jimpy* and wild-type cultures after 1 day of oligodendrocyte differentiation by gene set enrichment analysis (GSEA) (Table S1), at the onset of *jimpy* cell death and upregulation of the disease-causative gene, *Plp1* (Figure 2A). *Jimpy* cultures showed multiple dysregulated pathways comprising several gene sets linked to the ER stress response (Figure 2B; Table S1), including the unfolded protein response (UPR) pathway (Figures 2C and 2D).

We next sought to precisely define the cell types exhibiting these pathological stress signatures using single-cell RNA sequencing (scRNA-seq). Unsupervised clustering separated *jimpy* and wild-type cells in t-distributed stochastic neighbor embedding (tSNE) space (Figure 2E), while also arranging OPCs, nascent oligodendrocytes, nascent astrocytes, and transitioning cells (between OPC and nascent oligodendrocyte) into distinct populations defined by marker expression (Figures 2F and S3B–S3G). We then generated a list of differentially expressed genes between genotypes for each cell type present in both cultures (Table S2). GSEA on the nascent oligodendrocyte populations revealed a significant enrichment of ER stress and cell death pathways in this early *jimpy* oligodendrocyte population (Figures 2G; Table S3), confirming the presence of canonical PMD molecular pathology in the disease-affected cell type.

Interestingly we also noted enrichment for similar pathways in nascent *jimpy* astrocytes, indicating possible astrocyte contribution to disease in PMD (Skoff, 1976). However these transcriptional changes could simply represent a response to the substantial cell death in the cultures. To test this, we first directed iPSC-derived *jimpy* and wild-type OPCs toward an astrocyte fate. We observed no difference in the percentage of GFAP+ astrocytes in wild-type and

(C–E) Wild-type and *jimpy* oligodendrocyte differentiation time-course data showing quantification of total (C) DAPI+ cells, (D) O4+ cells, and (E) MBP+ oligodendrocytes. n = 6 replicate wells per genotype, with each replicate value represented by a white circle. (F) Representative immunocytochemistry images from time course showing MBP+ oligodendrocytes (green), O4+ cells (red), and total cells (DAPI, blue).

Scale bars, 50 μm. Error bars represent mean ± SD. See also Figures S1 and S2, and Video S1.

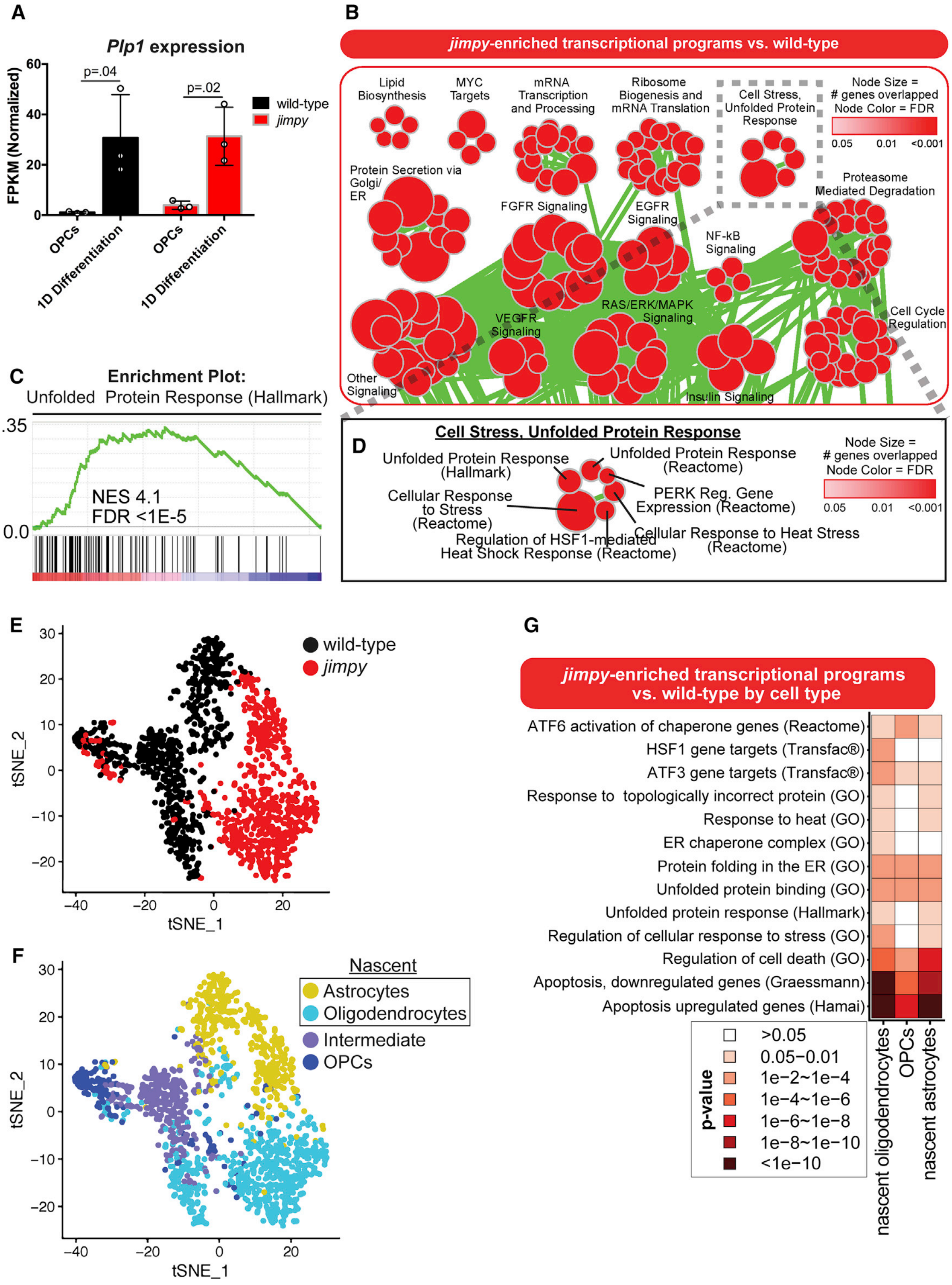


**Figure 2. RNA-Seq and scRNA-Seq Reveal Transcriptome Signatures of ER Stress and Cell Death in Early, Differentiating *Jimpy* Oligodendrocytes**

(A) *Plp1* mRNA expression in wild-type and *jimpy* OPCs and in cultures during oligodendrocyte (at day 1). Error bars represent mean ± SD. n = 3 sequenced cell lines per genotype, with each replicate represented by a white circle. p values calculated using a two-way, unpaired t test for each genotype.

*(legend continued on next page)*

*jimpy* cultures (Figures S3H–S3J). Next we performed astrocyte co-culture experiments to examine the effect of *jimpy* and wild-type astrocytes on oligodendrocyte survival. This analysis revealed no astrocyte-mediated effect on mutant or wild-type oligodendrocytes (Figures S3K–S3P). Together these data demonstrate that astrocytes are not the primary mediators of oligodendrocyte death in *jimpy* cultures and that pathological changes in *jimpy* astrocytes are likely secondary to primary oligodendrocyte death.

## High-Throughput Screening Reveals Chemical Enhancers of *Jimpy* Oligodendrocyte Survival

Our stem cell platform provides scalable access to a defined developmental window for potential therapeutic intervention in *jimpy*. To explore whether small molecules could be used to resolve the molecular and cellular pathology in early differentiating *jimpy* OPCs, we developed a chemical screening pipeline to identify compounds that improved the survival of MBP+ *jimpy* oligodendrocytes in conditions promoting oligodendrocyte differentiation (Figure 3A). We first performed a hypothesis-driven compound screen to interrogate the protein kinase R-like ER kinase (PERK) arm of the UPR, as well as a variety of cell death pathways that could drive oligodendrocyte loss during unresolved ER stress (Figure 3B) (Nevin et al., 2017). Using *jimpy* iPSC-derived OPCs we applied each compound in triplicate over a 5-point dose curve (10 μM to 625 nM) with simultaneous initiation of oligodendrocyte differentiation with thyroid hormone and then quantified MBP+ oligodendrocyte number after 3 days. Out of five compounds that modulate distinct targets in the PERK pathway, only salubrinal (Figures 3C–3E), a dual GADD34 and CReP inhibitor (Boyce et al., 2005), showed a dose-dependent ability to enhance *jimpy* oligodendrocyte survival. Examination of cell death-modulating compounds identified the high-efficacy pan-caspase inhibitors, emricasan and Q-VD-OPh, as effective mediators of *jimpy* oligodendrocyte death (Figures 3C, 3D, 3F, and 3G). Strikingly, the combination of Q-VD-OPh and salubrinal demonstrated an additive ability to restore *jimpy* cell number back to wild-type levels and enhance *jimpy* oligodendrocyte survival (Figures 3H, 3I, and S4). Collectively, these data show that loss of *jimpy* oligodendrocytes can be prevented by modulating ER stress and caspase-mediated cell death during the immediate transition from OPC to oligodendrocyte.

We scaled this screening pipeline to an unbiased collection of >3,000 bioactive compounds to identify additional nodes of intervention for *jimpy* pathology. To gauge screening performance we included the combination of salubrinal plus Q-VD-OPh as a positive control and a DMSO vehicle negative control. We performed a high-throughput primary screen at a single dose of 10 μM followed by rigorous secondary screening to filter to a lead compound (Figure 4A). We evaluated the performance of our primary screen based on the dynamic range of quantified oligodendrocytes between our positive and negative controls using the *Z* factor ($Z' = 0.566 \pm 0.23$ [mean ± SD]) (Figures S5A–S5C), indicating a high quality and reproducible screening assay based on established performance analytics (Zhang et al., 1999). Automated image analysis scored each well based on enhancement of MBP+ oligodendrocyte number on a per-plate basis (Figure 4C). As an orthogonal filter we also used total cell number as an overall marker of increased cell survival. All compounds were ranked (Table S4) and our top 64 hits (Figure 4B) were defined as being at least four SDs above the negative control for oligodendrocyte number and two SDs above negative control for total cell number. Of note, three of the top compound hits included Q-VD-OPh, salubrinal, and emricasan (Figure 4B), which were identified and validated in our previous hypothesis-driven chemical screen.

Next we used a stringent secondary screening pipeline to prioritize these hits to a single lead compound (Figure 4A). First, all hits were tested in OPCs generated from two additional independent *jimpy* iPSC lines using the same primary screen dose of 10 μM and primary screen hit selection criteria. The efficacy of 55 out of 64 hits was confirmed in at least one additional *jimpy* cell line (Table S5). Next, each confirmed compound was examined over an 8-point dose response (from 10 μM to 78 nM), and compounds were ranked by their potency to enhance oligodendrocyte survival (Figure 4D; Table S5). This analysis highlighted the lead compound Ro 25–6981 (Figures 4D and 4E; Table S5), which showed efficacy far superior even to the

(B) Gene ontology enrichment map for RNA-seq data showing enriched pathways in *jimpy* after 1 day of oligodendrocyte differentiation. Node size indicates the number genes represented in the pathway. Color intensity indicates false discovery rate (FDR). Node overlap indicates shared pathway genes. n = 3 cell lines, per genotype.
(C) *Jimpy* enrichment plots for the hallmark unfolded protein response pathway genes relative to wild-type. The upper plot (green line) details the running sum statistic of the ranked list of genes with the normalized enrichment score (NES) and FDR. The lower plot displays a gene's position in the ranked list of genes for the pathway.
(D) Enlargement of the "cell stress, unfolded protein response" node group.
(E and F) tSNE plots showing (E) genotype and (F) cell-type distributions. n = 1 cell line per genotype.
(G) Heatmap showing pathway enrichment in *jimpy* cell types relative to their wild-type counterparts. Scale corresponds to p value.
See also Figure S3, Tables S1, S2, and S3.

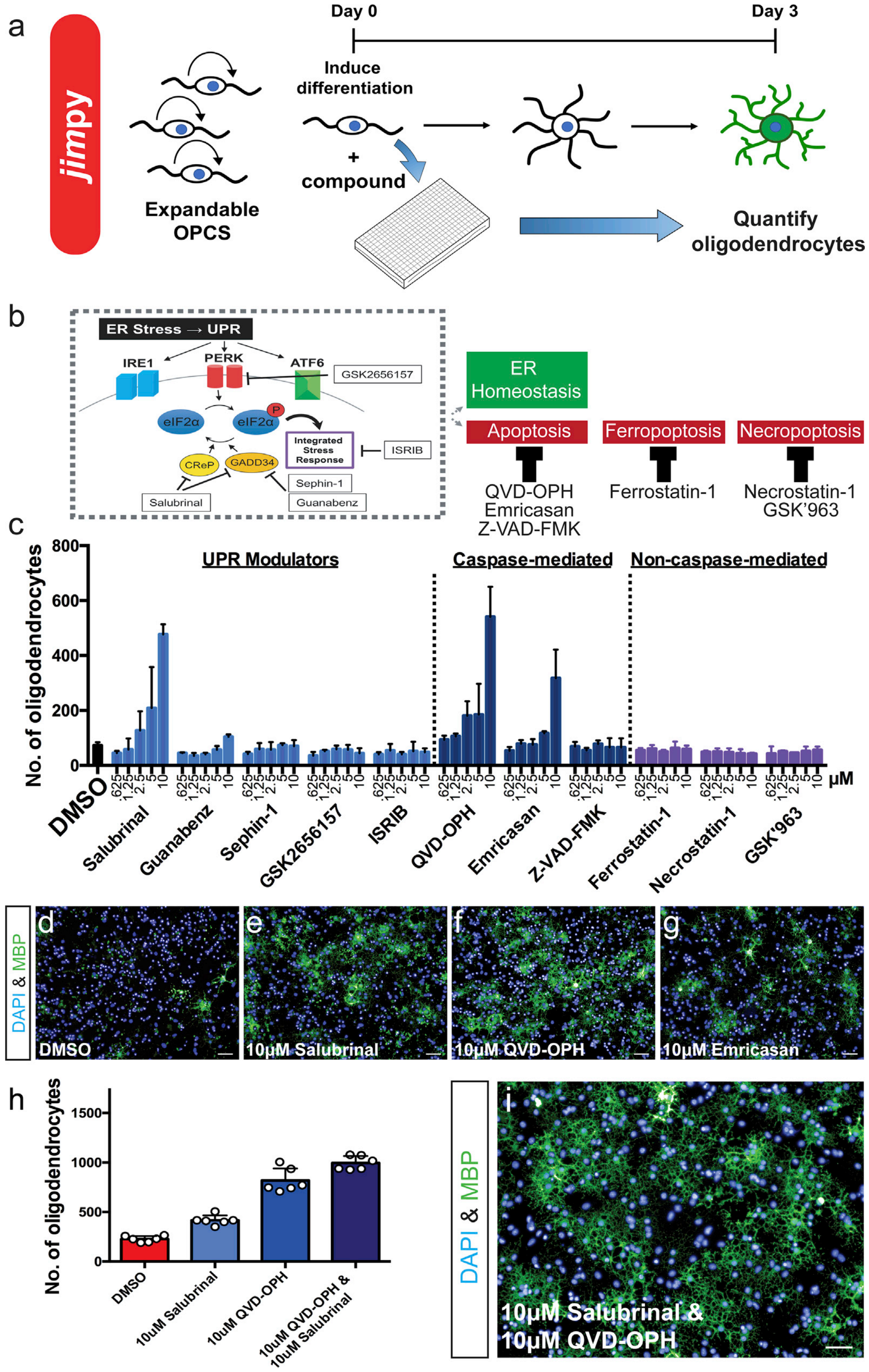


*(legend on next page)*

combination of salubrinal and Q-VD-OPh (Figure 4D), our screening positive control.

Next we initiated a rigorous validation procedure to comprehensively define Ro 25–6981's molecular and cellular effects in multiple, orthogonal contexts (Figure 4F). We first sought to assess the potency of Ro 25–5981 using a 10-point dose response (from 10 μM to 20 nM). This analysis revealed strong efficacy extending to nanomolar doses (half maximal effective concentration [$EC_{50}$] = 168.3 nM) (Figures 4G and 4H). To rule out possible iPSC-derived OPC artifacts we demonstrated that Ro 25–6981 had remarkably similar dose-dependent efficacy and potency to enhance oligodendrocyte survival in primary cultures of OPCs isolated from postnatal *jimpy* mice ($EC_{50}$ = 524.0 nM) (Figures 4I and 4J).

To examine if the cellular or molecular effects of Ro 25–6981 were specific to mutant cells we first assessed oligodendrocyte enhancement using Ro 25–5981 in a 10-point dose response (from 10 μM to 20 nM) or DMSO vehicle on cultures of differentiating wild-type and *jimpy* OPCs. In addition we also examined the effects of our *jimpy* cellular pathology positive control compounds, salubrinal and Q-VD-OPh. As expected all compounds robustly (~3- to 5-fold) enhanced *jimpy* oligodendrocytes (Figure S6B). In contrast these same compounds showed only a modest (~1.5- to 2-fold) enhancement of wild-type oligodendrocytes (Figure S6A).

### Mechanistic Studies Reveal a Non-canonical, UPR-Mediator Role for Ro 25–6981 in Differentiating *Jimpy* OPCs

Ro 25–6981 is an NR2B-selective N-methyl-D-aspartate (NMDA) receptor antagonist (Fischer et al., 1997). However, we noted absent expression in our RNA-seq data profiling OPC and early oligodendrocyte differentiation in all iPSC-derived wild-type and *jimpy* cell cultures (fragments per kilobase of transcript per million mapped reads < 0.15; Table S1), consistent with publicly available datasets (Zhang et al., 2014). Still, a handful of studies have documented expression of the NR2B subunit in oligodendrocytes and astrocytes in normal and pathological contexts (Karadottir et al., 2005; Krebs et al., 2003; Salter and Fern, 2005).

To address the possibility of an NR2B-mediated effect we first sought to investigate *Grin2b* (NR2B subunit gene) expression throughout the OPC to oligodendrocyte transition using time course qRT-PCR in iPSC-derived *jimpy* and wild-type cultures. This comprehensive profiling failed to detect *Grin2b* expression at any time point (Figure S6C). We also performed functional testing of 29 unique NMDA receptor modulators (including NR2B-selective and pan-NMDA receptor antagonist drugs with high potency to their annotated targets) in 10-point dose response (from 10 μM to 20 nM) on iPSC-derived *jimpy* OPCs. These data failed to establish a correlation between NR2B-NMDA receptor antagonism and the enhancement of *jimpy* oligodendrocyte survival (Table S6). Furthermore, structure-activity relationship analysis revealed that only the Ro 25–6981 scaffold could potently increase *jimpy* oligodendrocyte survival. Together, these data eliminated the NR2B-NMDA receptor as the functional target in *jimpy* cultures.

To investigate alternative mechanisms for Ro 25–6981's action on *jimpy* oligodendrocyte survival *in vitro* we employed targeted qRT-PCR assessment during *jimpy* and wild-type oligodendrocyte differentiation. First we assessed Ro 25–6981's ability to alter *Plp1* expression levels as reduction of mutant PLP is beneficial to PMD cellular and animal models (Karim et al., 2010; Prukop et al., 2014). We found no effect of Ro 25–6981 on *Plp1* induction in *jimpy* (Figure 5A). We next examined Ro 25–6981's ability to modulate *jimpy*-dysregulated UPR-related factors curated from our transcriptome data including *Ddit3*, *Hspa5*, *Dnajb9*, spliced-*Xbp1* (s-*XBP1*), *Atf4*, and *Atf6*. As a comparator we also assessed the effect of the known UPR modulator salubrinal. Both compounds showed reductions of *Dnajb9* and *s-Xbp1* to wild-type levels, no change in *Atf6*, and increased expression of *Hspa5* (Figure 5B). Of note,

**Figure 3. High-Throughput Chemical Screening Identifies Cellular and Molecular Modulators of *Jimpy* Pathology**
(A) Chemical screening schematic.
(B) Chemical-targeting of ER stress-related sequelae by UPR modulation or inhibition of caspase-mediated apoptosis.
(C) Quantification of MBP+ oligodendrocytes per compound dose (five doses, 10 μM to 625 nM) after 3-day oligodendrocyte differentiation of iPSC-derived *jimpy* OPCs. n = 3 replicate wells per dose, per compound.
(D–G) Representative immunocytochemistry images after oligodendrocyte differentiation of *jimpy* OPCs treated with (D) DMSO vehicle, (E) 10 μM salubrinal, (F) 10 μM Q-VD-OPh, and (G) 10 μM emricasan, showing MBP+ oligodendrocytes (green) and total DAPI+ cells (blue).
(H) Quantification of MBP+ *jimpy* oligodendrocytes for DMSO vehicle (red), 10 μM salubrinal (light blue), 10 μM Q-VD-OPh (blue), and 10 μM salubrinal and 10 μM Q-VD-OPh (dark blue). n = 6 replicate wells per treatment, with each replicate value represented by a white circle.
(I) Representative immunocytochemistry images after oligodendrocyte differentiation of *jimpy* OPCs treated with 10 μM salubrinal and 10 μM Q-VD-OPh, showing MBP+ oligodendrocytes (green) and total DAPI+ cells (blue).
Error bars represent mean ± SD. Scale bars, 50 μm. See also Figure S4.

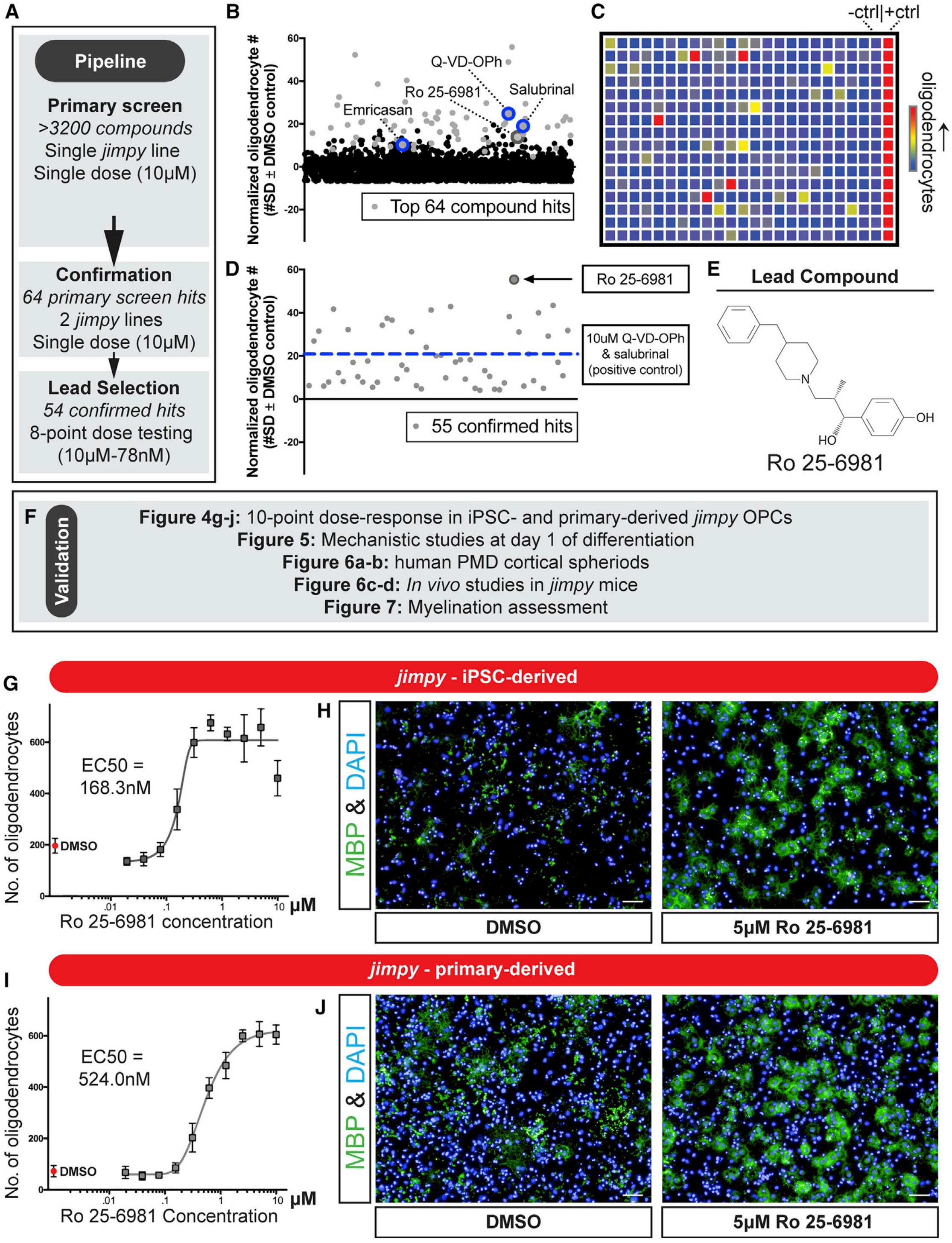


**Figure 4. High-Throughput Chemical Screening Identifies Cellular and Molecular Modulators of *Jimpy* Pathology**

(A) Overview of screening pipeline with filtering steps for lead compound selection.

(B) Plot of primary screen results showing top hit (gray circles) and non-hit (black circles) compounds listed by alphabetical order with their corresponding SD divergence from DMSO vehicle on the MBP+ *jimpy* oligodendrocyte metric after 3-day oligodendrocyte differentiation. n = 1 well per tested compound.

(legend continued on next page)

salubrinal restored *Atf4* to wild-type levels while Ro 25–6981 showed no change. In contrast Ro 25–6981 modestly increased Ddit3, above the salubrinal-mediated effect (Figure 5B). Interestingly, while these compounds produced remarkably similar profiles, their points of divergence may provide a clue to their vastly different efficacies in *jimpy* oligodendrocyte restoration. The *Ddit3* and *Hspa5* results are especially intriguing as depletion of DDIT3 has been shown to exacerbate disease in PMD animal models (Southwood et al., 2002), while reduction of HSPA5 is deleterious to oligodendrocyte survival in both normal and autoimmune disease contexts (Hussien et al., 2015) and is depleted in PMD models (Numata et al., 2013).

We further examined these UPR expression effects testing Ro 25–6981 as well as salubrinal on differentiating wild-type cultures. Surprisingly the vast majority of the transcriptional changes were *jimpy* specific, with *Atf6* and *Ddit3* even showing completely opposite trends in the Ro 25–6981 condition (Figure S6D). Together these data suggested that Ro 25–6981 functioned by modulating the UPR in a genotype-specific manner that was highly specific to *jimpy* cultures.

### Ro 25–6981 Increases Oligodendrocytes in Human PMD Patient iPSC-Derived Cultures and *Jimpy* Mice

To test if Ro 25–6981's effect was mutation or organism specific, we applied this compound to human PMD oligocortical spheroids (Madhavan et al., 2018) generated using iPSCs from a severe connatal PMD patient with a *PLP1*$^{c.254T>G}$ point mutation (Nevin et al., 2017). We applied 1 μM Ro 25–6981 or DMSO vehicle to mutant spheroids during a 20-day period of oligodendrocyte generation and quantified oligodendrocytes as a percentage of total cell number using an antibody to the oligodendrocyte-specific transcription factor, myelin regulatory factor (MyRF). In addition, we administered DMSO vehicle to control, isogenic gene-corrected spheroids. Ro 25–6981 treatment profoundly increased the percentage of mutant oligodendrocytes in the human PMD spheroids (Figures 6A and 6B), confirming the ability to target common cellular pathology and enhance mutant oligodendrocyte survival across distinct PMD genotypes and species. Of note these levels of oligodendrocytes were still below those of the isogenic control, suggestive of remaining pathology, potentially also reflected by the unrestored PLP expression pattern (Figure 6A).

Next we examined if Ro 25–6981 treatment could increase oligodendrocytes in *jimpy* mice, *in vivo*. We administered 1 or 10 mg/kg Ro 25–6981 or saline vehicle to *jimpy* mice and saline vehicle to wild-type mice from postnatal days 5–14, and quantified MyRF+ oligodendrocytes along the medial rostral caudal axis of the corpus callosum. Ro 25–6981 treatment significantly increased *jimpy* oligodendrocytes in a dose-dependent manner, but below wild-type levels (Figures 6C and 6D). These results confirmed that Ro 25–6981's effect was not limited to *in vitro* contexts and could enhance oligodendrocyte survival in *jimpy* mice *in vivo*.

### Increasing *Jimpy* Oligodendrocyte Survival Uncovers a Second Pathological Phase during Myelination

Oligodendrogenesis is thought to be closely coupled to myelination (Mayoral and Chan, 2016)—a process which involves the generation and extension of specialized oligodendrocyte membrane around neuronal axons. Given the strong enhancement of early oligodendrocyte survival across multiple contexts we expected to see a frank restoration of myelinating oligodendrocytes. Surprisingly we found only a slight increase in myelinating cells in Ro 25–6981-treated *jimpy* mice as marked by MBP (Figure 6C).

To appreciate why increased oligodendrocyte survival did not translate to a broad restoration of myelin we employed a defined system using non-biological poly-lactide microfibers. These microfibers serve as a robust, reproducible, and scalable myelination substrate (Bechler et al., 2015). We plated *jimpy* OPCs in the presence of Ro 25–6981 in an 8-point (10 μM to 78 nM) dose-response

(C) Heatmap of a representative primary screen plate depicting high (red) or low (blue) quantified MBP+ *jimpy* oligodendrocytes for each compound, as well as negative and positive controls.
(D) Plot of confirmed hits (gray circles) listed in alphabetical order showing their maximal SD divergence from DMSO vehicle for MBP+ *jimpy* oligodendrocytes in an 8-point (10 μM to 78 nM) dose response. n = 1 well per compound dose. n = 16 replicate wells for the positive and negative control samples.
(E) Chemical structure of lead compound Ro 25–6981.
(F) Overview of validation pipeline for lead compound Ro 25–6981.
(G and I) Quantification of MBP+ *jimpy* oligodendrocytes after differentiation of (G) iPSC-derived and (I) cerebral cortical-derived OPCs, treated with Ro 25–6981 (10-point dose response) or DMSO vehicle. Individual points represent mean ± SD. n = 3 replicate wells for each dose, n = 16 replicate wells for the DMSO vehicle control.
(H and J) Representative immunocytochemistry images after oligodendrocyte differentiation of (H) iPSC-derived or (J) cerebral cortical-derived OPCs treated with DMSO vehicle or 5 μM Ro 25–6981 showing MBP+ oligodendrocytes (green) and total DAPI+ cells (blue). Scale bars, 50 μm. See also Figure S5, Tables S4, and S5.

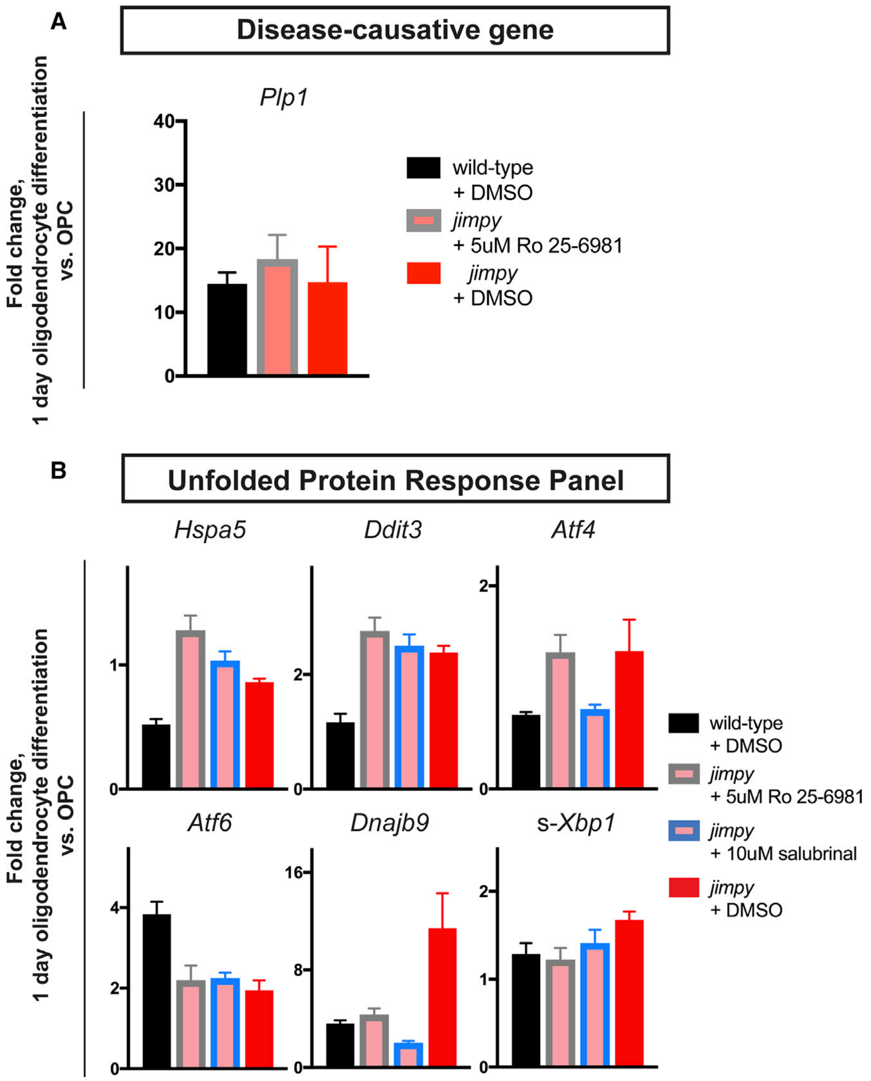


**Figure 5. Ro 25–6981 Mechanistic Studies Reveal UPR Modulation in *Jimpy***

(A) qRT-PCR of *Plp1* for wild-type and *jimpy* at day 1 of oligodendrocyte differentiation with indicated treatment. Vehicle-treated controls same as Figure S6D. For *Atf6* n = 3 technical replicates per sample. n = 4 technical replicates per sample.

(B) qRT-PCR of UPR-related panel for wild-type and *jimpy* at day 1 of oligodendrocyte differentiation with indicated treatment. Vehicle-treated controls same as Figure S6D. For *Atf6* n = 3 technical replicates per sample. For all other probes n = 4 technical replicates per sample.

Error bars represent mean ± SD. See also Figure S6 and Table S6.

format or DMSO vehicle. To account for Ro 25-6981-specific effects on myelination we also tested our *jimpy* cellular pathology positive control compounds, salubrinal and Q-VD-OPh. We induced oligodendrocyte differentiation, allowed for microfiber ensheathment (*in vitro* "myelination"), and quantified MBP+ myelinating oligodendrocytes (Figure 7A) using an automated image analysis pipeline (Figures S7A–S7E). Despite showing a robust, dose-dependent increase in *jimpy* oligodendrocyte survival and early myelination (Figures 7B and 7D), Ro 25–6981, salubrinal, and Q-VD-OPh were unable maintain myelin by day 10 across all tested doses. This result was specific to *jimpy*, as wild-type DMSO vehicle-treated cultures showed extensive myelination at day 10 (Figures 7C and 7E). The ability to restore *jimpy* oligodendrocytes without a subsequent restoration of mutant myelin was unexpected and revealed that there are two distinct stages of disease pathology: (1) ER stress-mediated cell death as cells transition from OPCs to oligodendrocytes and (2) dysfunctional myelination due to the presence of mutant PLP protein in surviving oligodendrocytes. Collectively, our results show that the first phase can be resolved with small molecules and that effective clinical intervention for PMD will need to address both phases of pathology.

## DISCUSSION

In this study we established a genotype-specific, scalable, and tractable system to investigate a severe PMD point mutation modeled in the *jimpy* mouse. Coupled with stringent control over oligodendrocyte differentiation, this system enabled precise, temporal dissection of *jimpy* pathology. Using a simple developmental trigger (induction of

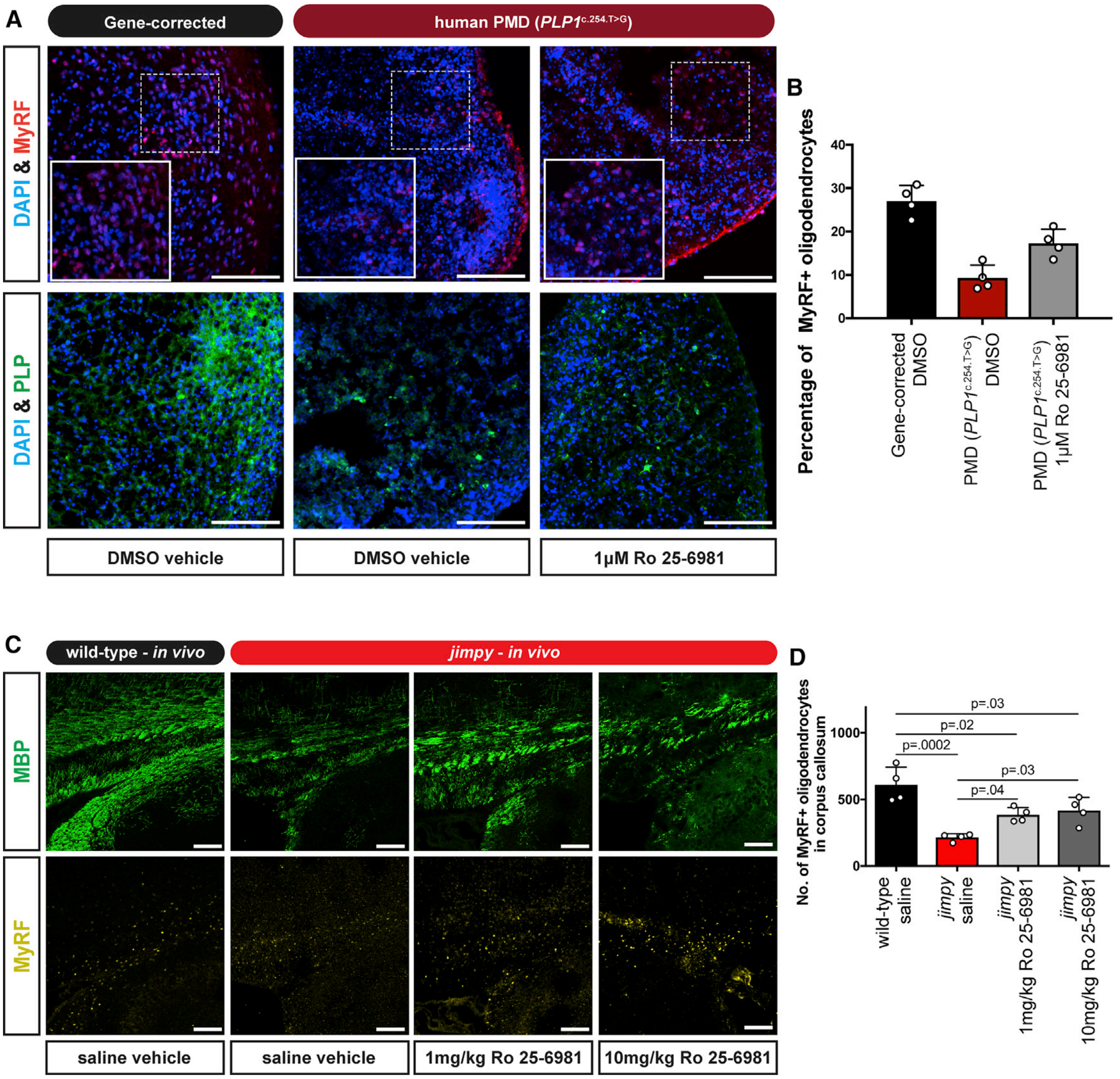


**Figure 6. Ro 25–6981 Enhances Mutant Oligodendrocyte Survival in Human Patient PMD Spheroids and in *Jimpy* Mice**

(A) Representative immunocytochemistry images of human PMD patient (*PLP1*$^{c.254.T>G}$) and wild-type iPSC-derived oligocortical spheroids showing MyRF+ (red) and PLP+ (green) oligodendrocytes, as well as total DAPI+ cells (blue) after treatment.

(B) Percentage of MyRF+ oligodendrocytes relative to total DAPI+ cells in human PMD patient (*PLP1*$^{c.254.T>G}$) and wild-type iPSC-derived oligocortical spheroids after treatment. n = 4 independent spheroids per treatment.

(C) Representative immunocytochemistry images of the medial corpus callosum of wild-type and *jimpy* mice after treatment showing MBP+ oligodendrocytes (green) and MyRF+ oligodendrocytes (yellow).

(D) Quantification of MyRF+ oligodendrocytes within the medial corpus callosum of wild-type and *jimpy* mice after treatment. n = 4 injected animals per treatment.

Scale bars, 100 μm. Error bars represent mean ± SD, with each replicate value indicated by a white circle. p values calculated using a one-way ANOVA with Holm-Sidak correction for multiple comparisons.

oligodendrocyte differentiation) we recapitulated known cellular and molecular PMD phenotypes including ER stress and oligodendrocyte death (Ikeda et al., 2018; Southwood et al., 2002), and defined a surprisingly early susceptibility period. Chemical screening demonstrated a reversal of *jimpy* oligodendrocyte loss by inhibiting specific targets in the UPR and apoptosis pathways. Furthermore, this system allowed us to identify Ro 25–6981, a highly effective and potent modulator of *jimpy* pathology with a previously undefined effect on the UPR.

While our studies were designed to identify cellular and molecular phenotypes as well as compound modulators on a genotype-specific basis, we reasoned that UPR effectors could counteract the sequelae seen in alternate,

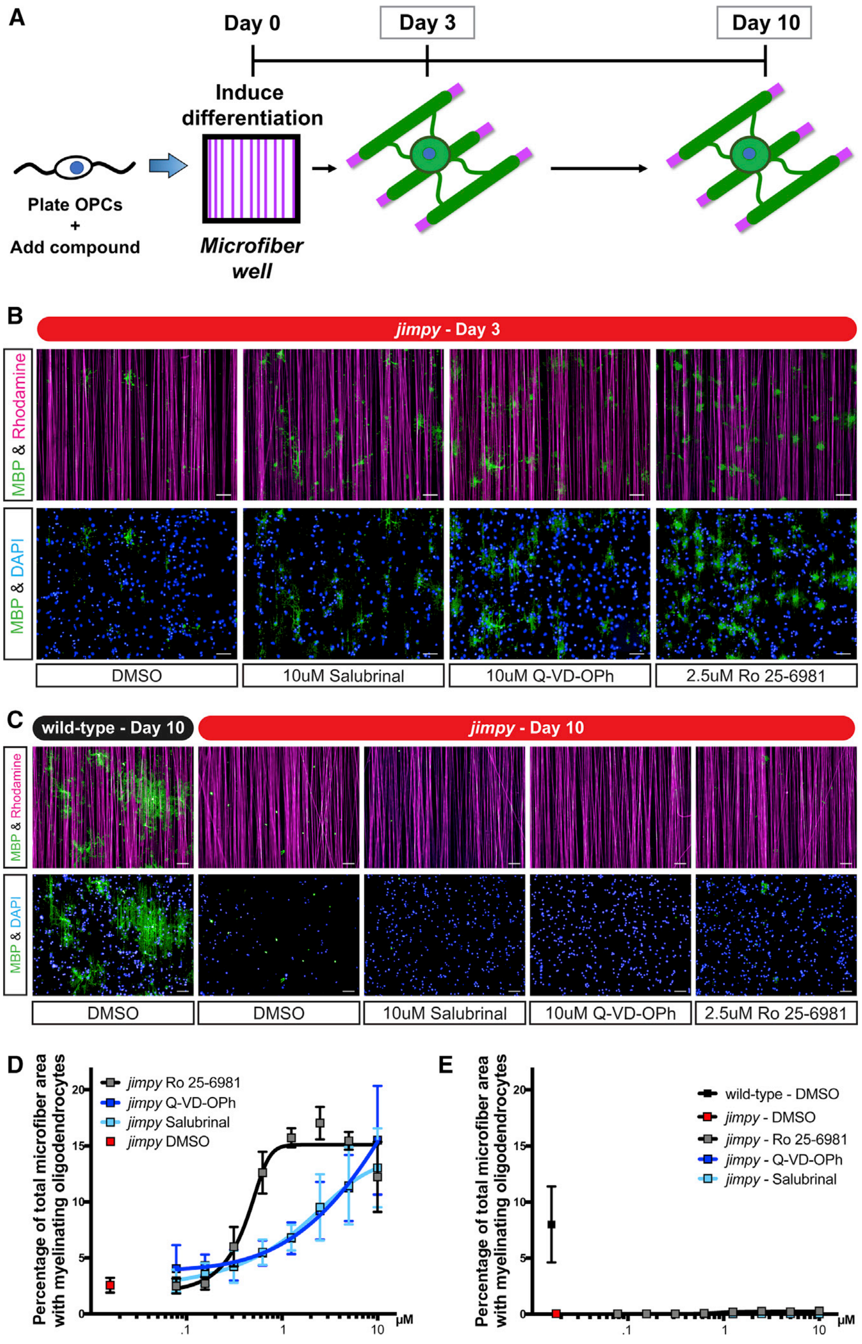


**Figure 7. Increased *Jimpy* Oligodendrocyte Survival during Differentiation Unmasks a Second Pathological Phase during Myelination**

(A) Schematic of the *in vitro* myelination assay using 4-μm diameter synthetic microfiber fibers detailing the examination of myelinating oligodendrocytes at day 3 and 10.

(B and C) Representative immunocytochemistry images of MBP+ myelinating oligodendrocytes (green), rhodamine+ microfibers (purple), and total DAPI+ cells (blue) at (B) day 3 and (C) day 10. Compound-treated cultures shown at their maximum effective concentration.

(D and E) Quantification of percent total microfiber area overlap with MBP+ myelinating oligodendrocytes at (D) day 3 and (E) day 10 with indicated treatment and genotype. Scale bars, 50 μm. n = 3 replicate wells per compound treatment. n = 16 replicate wells per vehicle control.

Error bars represent mean ± SD. See also Figure S7.

human PMD mutations by targeting common pathology in this disease (Nevin et al., 2017). We tested Ro 25–6981 on PMD patient-derived human oligocortical spheroids and demonstrated a robust increase in mutant oligodendrocytes, confirming efficacy in human disease-relevant tissue and suggesting modulation of shared disease components.

Given the striking enhancement of mutant oligodendrocytes across multiple contexts (cellular, genotypic, and species), we expected to observe a substantial increase in myelin after Ro 25–6981 treatment, *in vivo*. While Ro 25–6981 increased the quantity of *jimpy* oligodendrocytes, it did not restore myelination to wild-type levels. As oligodendrogenesis and myelination are thought to be distinct processes (Mayoral and Chan, 2016), we examined myelination in *jimpy* with Ro 25–6981 treatment using non-biologic, synthetic microfibers. These data revealed that Ro 25–6981 was unable to restore myelination in

*jimpy*, revealing a second stage of disease, refractory to treatment that enhances early oligodendrocyte survival. As such, our results establish a dual therapeutic paradigm: (1) restore oligodendrocyte loss during differentiation and then (2) enhance myelination capacity of surviving, mutant oligodendrocytes. Importantly, approaches that simply target these rare, surviving oligodendrocytes are unlikely to be successful without first enhancing the survival during the early OPC to oligodendrocyte transition. This understanding has substantial impact on therapeutic development for PMD and potentially other disorders where ER stress is implicated.

The dichotomy of compound-mediated increased mutant oligodendrocyte survival without increased myelination could be explained by the unique demands of the latter process, which involves immense synthesis of myelin lipids and proteins (Baron and Hoekstra, 2010), including PLP which has been estimated to constitute approximately 50% of the total myelin protein (Baumann and Pham-Dinh, 2001). As such the myelination stage may necessitate engagement of completely separate targets for pharmacological interventions. Prior studies by us and others have shown, paradoxically, that modulating opposing responses in the UPR pathway, including inhibition of PERK or GADD34, leads to phenotypic benefit in PMD and other myelinating disorders (D'Antonio et al., 2013; Musner et al., 2016; Nevin et al., 2017). Future studies will need to parse the precise target of Ro 25–6981's UPR-modulating effect and assess the potential use of combinatorial therapies.

In addition, the presence mutant PLP protein could lead to specific sequelae (Koizume et al., 2006; Kramer-Albers et al., 2006; Roussel et al., 1987), distinct from the pathology that develops immediately after *jimpy* OPCs begin to differentiate to oligodendrocytes. Interestingly, *PLP1* duplications, which constitute ~70% of human patients (Inoue, 2005), may be especially amendable to UPR-modulating compounds such as Ro 25–6981, as these cases are typified by overly abundant but normal PLP protein. As such, moderating ER overload may be a viable strategy to restore oligodendrocytes and myelin in these patients.

More broadly, given that ER stress is a fundamental component of many diseases of myelin including other leukodystrophies and multiple sclerosis (Clayton and Popko, 2016; Way et al., 2015), *jimpy* modulators such as Ro 25-6981 may provide effects beyond PMD, to other genetic disorders of myelin and multiple sclerosis.

## EXPERIMENTAL PROCEDURES

### Animal Studies

All animal procedures and experimentation was approved by Case Western Reserve University's Institutional Animal Care and Use Committee.

### Generation of *Jimpy* iPSCs

Tail-tip fibroblasts were derived from *jimpy* mutant and wild-type littermates reprogrammed to iPSCs using a lentivirus encoding a doxycycline-inducible polycistronic Oct4, Sox2, Klf4, and c-Myc construct as described previously (Somers et al., 2010; Sommer et al., 2009).

### Generation of *jimpy* OPCs

iPSCs were differentiated to high purity, expandable OPCs as described previously (Najm et al., 2011) (Lagar et al., 2018). OPCs were propagated in DMEM/F12 (11320082, Thermo Fisher Scientific), 1× N2 supplement (AR009, R&D Systems), 1× B-27 without vitamin A supplement (12,587-010, Thermo Fisher Scientific), and 1× Glutamax, supplemented with 20 ng/mL fibroblast growth factor 2 (233-FB, R&D Systems) and 20 ng/mL platelet-derived growth factor-AA (221-AA, R&D Systems).

### Differentiation of *Jimpy* OPCs to Oligodendrocytes

OPCs were differentiated to oligodendrocytes with differentiation medium that consisted of DMEM/F12, 1× N2 supplement, 1× B-27 without vitamin A supplement, supplemented with 100 ng/mL noggin (3344-NG, R&D Systems), 10 ng/mL neurotrophin-3 (NT-3) (267-N3, R&D Systems), 50 μM cAMP (D0260, Sigma), 100 ng/mL insulin-like growth factor-1 (291-G1, R&D Systems) NT-3, and 40 ng/mL triiodothyronine (thyroid hormone; T-6397, Sigma).

### Compound Screening

OPCs were seeded on 384-well poly-D-lysine CellCarrier Ultra plates (6057500, PerkinElmer), compound was added, and oligodendrocyte differentiation was induced. Three days later plates were fixed with 4% paraformaldehyde (PFA), and immunostained using rat anti-MBP and goat anti-Sox10, followed by counterstaining with DAPI. Images were captured and quantified using the Operetta High Content Imaging and Analysis system (PerkinElmer), Harmony software (PerkinElmer), and Acapella software (PerkinElmer).

### qRT-PCR

RNA was prepared with the RNeasy Mini Kit (74104, QIAGEN). qRT-PCR was performed using pre-designed TaqMan gene expression assays (Thermo Fisher Scientific) and probes values were normalized to an *Actb* endogenous control.

### Human PMD Cultures

Human PMD patient (*PLP1*$^{c.254T>G}$) and gene-corrected (isogenic control) iPSCs were previously generated as described (Nevin et al., 2017) and used to generate oligocortical spheroids (Madhavan et al., 2018). Spheroids were treated with DMSO vehicle or Ro 25–6981 from days 60 to 90. Spheroids were fixed with 4% PFA, and immunostained using rabbit anti-MyRF and rat anti-PLP, followed by counterstaining with DAPI. Images were captured and quantified using Adobe Photoshop (Abobe Systems).

### In Vivo *Jimpy* Studies

*Jimpy* and wild-type control mice were administered Ro 25–6981 (1594, R&D Systems) or saline vehicle from postnatal day 5

to 14. Mice were sacrificed and tissue sections were immunostained using rabbit anti-MyRF and rat anti-MBP antibody. Complete corpus callosum (medial, sagittal sections) images were captured and quantified using Adobe Photoshop.

### *In Vitro* Myelination Assay

OPCs were seeded on Mimetix 384-well plates containing synthetic microfibers (AMS-TECL-015, AMSBio), compound was added, and oligodendrocyte differentiation was induced. At day 3 and 10 after seeding, plates were fixed with 4% PFA, and immunostained using rat anti-MBP, followed by counterstaining with DAPI. Images were captured and quantified using the Operetta High Content Imaging and Analysis system, Harmony software, and Acapella software.

### Quantitation and Statistical Analyses

GraphPad Prism was used to perform all statistical analyses unless otherwise indicated. Statistical tests as well as replicate descriptions are detailed in each figure legend. A p value < 0.05 was considered significant for all analyses, unless otherwise denoted.

## ACCESSION NUMBERS

The accession number for the RNA-seq data reported in this paper is GEO: GSE111605.

## SUPPLEMENTAL INFORMATION

Supplemental Information includes Supplemental Experimental Procedures, seven figures, six tables, and one video and can be found with this article online at https://doi.org/10.1016/j.stemcr.2018.07.015.

## AUTHOR CONTRIBUTIONS

M.S.E. and P.J.T. conceived and initiated the project. H.E.S. and M.S.E. harvested animal tissues. D.C.F. and T.E.M. generated and analyzed the RNA-seq data, and generated the associated figures. C.W., Y.L., and F.J. performed Drop-Seq, data analysis, and generated the associated figures. B.L.L.C. and M.S.E. executed the astrocyte studies. K.C.A., L.B., and M.S.E. performed qRT-PCR and analyzed the data. H.E.S., M.M., and M.S.E. isolated primary cell cultures. D.J.A. and M.S.E. designed the high-throughput primary compound screen and analyzed the data. M.M., L.B., and B.S.N. carried out immunohistochemistry and quantifications. M.M., Z.S.N, M.S.E., and H.E.S. cultured human spheroids and generated associated figures. M.S.E. and P.J.T. wrote the manuscript with feedback from all authors.

## ACKNOWLEDGMENTS

This research was supported by grants from: the European Leukodystrophy Association (to P.J.T.); the NIH R01NS093357 (to P.J.T.), R01HG009658 (to F.J.), and T32GM007250 (to M.S.E.); the New York Stem Cell Foundation (to P.J.T); and philanthropic support from the Peterson, Fakhouri, Long, Goodman, Geller, Galbut/Heil, and Weidenthal families and the Research Institute for Children's Health. Additional support was provided by the Cytometry & Microscopy, Genomics, Imaging, and Small Molecule Drug Development core facilities of the Case Western Reserve University (CWRU) Comprehensive Cancer Center (P30CA043703), the High Performance Computing Resource in the Core Facility for Advanced Research Computing at CWRU, the Cleveland Clinic Lerner Research Institute Imaging Core, and the CWRU Light Microscopy Core Facility (NIH grant S10-OD016164). We are grateful to Zita Hubler, Yuriy Fedorov, and Brian Popko for technical assistance and/or discussion. P.J.T. and D.J.A. are co-founders and consultants for Convelo Therapeutics, which has licensed patents from CWRU inventors (P.J.T., D.J.A., and M.S.E.). P.J.T., D.J.A., and CWRU hold equity in Convelo Therapeutics. D.C.F. became an employee of Convelo Therapeutics subsequent to completion of these studies. P.J.T. is a consultant and on the Scientific Advisory Board of Cell Line Genetics. P.J.T. is Chair of the Scientific Advisory Board (volunteer position) for the Pelizaeus-Merzbacher Disease Foundation.

Received: June 22, 2018
Revised: July 30, 2018
Accepted: July 30, 2018
Published: August 23, 2018

## REFERENCES

Baron, W., and Hoekstra, D. (2010). On the biogenesis of myelin membranes: sorting, trafficking and cell polarity. FEBS Lett. *584*, 1760–1770.

Baumann, N., and Pham-Dinh, D. (2001). Biology of oligodendrocyte and myelin in the mammalian central nervous system. Physiol. Rev. *81*, 871–927.

Bechler, M.E., Byrne, L., and Ffrench-Constant, C. (2015). CNS myelin sheath lengths are an intrinsic property of oligodendrocytes. Curr. Biol. *25*, 2411–2416.

Bonkowsky, J.L., Nelson, C., Kingston, J.L., Filloux, F.M., Mundorff, M.B., and Srivastava, R. (2010). The burden of inherited leukodystrophies in children. Neurology *75*, 718–725.

Boyce, M., Bryant, K.F., Jousse, C., Long, K., Harding, H.P., Scheuner, D., Kaufman, R.J., Ma, D., Coen, D.M., Ron, D., et al. (2005). A selective inhibitor of eIF2alpha dephosphorylation protects cells from ER stress. Science *307*, 935–939.

Bradl, M., Bauer, J., Inomata, T., Zielasek, J., Nave, K.A., Toyka, K., Lassmann, H., and Wekerle, H. (1999). Transgenic Lewis rats overexpressing the proteolipid protein gene: myelin degeneration and its effect on T cell-mediated experimental autoimmune encephalomyelitis. Acta Neuropathol. *97*, 595–606.

Cerghet, M., Bessert, D.A., Nave, K.A., and Skoff, R.P. (2001). Differential expression of apoptotic markers in jimpy and in Plp overexpressors: evidence for different apoptotic pathways. J. Neurocytol. *30*, 841–855.

Clayton, B.L., and Popko, B. (2016). Endoplasmic reticulum stress and the unfolded protein response in disorders of myelinating glia. Brain Res. *1648*, 594–602.

D'Antonio, M., Musner, N., Scapin, C., Ungaro, D., Del Carro, U., Ron, D., Feltri, M.L., and Wrabetz, L. (2013). Resetting translational homeostasis restores myelination in Charcot-Marie-Tooth disease type 1B mice. J. Exp. Med. *210*, 821–838.

Duncan, I.D., Kondo, Y., and Zhang, S.C. (2011). The myelin mutants as models to study myelin repair in the leukodystrophies. Neurotherapeutics *8*, 607–624.

Fischer, G., Mutel, V., Trube, G., Malherbe, P., Kew, J.N., Mohacsi, E., Heitz, M.P., and Kemp, J.A. (1997). Ro 25-6981, a highly potent and selective blocker of N-methyl-D-aspartate receptors containing the NR2B subunit. Characterization in vitro. J. Pharmacol. Exp. Ther. *283*, 1285–1292.

Garbern, J.Y. (2007). Pelizaeus-Merzbacher disease: genetic and cellular pathogenesis. Cell. Mol. Life Sci. *64*, 50–65.

Gow, A., Southwood, C.M., and Lazzarini, R.A. (1998). Disrupted proteolipid protein trafficking results in oligodendrocyte apoptosis in an animal model of Pelizaeus-Merzbacher disease. J. Cell Biol. *140*, 925–934.

Green, A.J., Gelfand, J.M., Cree, B.A., Bevan, C., Boscardin, W.J., Mei, F., Inman, J., Arnow, S., Devereux, M., Abounasr, A., et al. (2017). Clemastine fumarate as a remyelinating therapy for multiple sclerosis (ReBUILD): a randomised, controlled, double-blind, crossover trial. Lancet *390*, 2481–2489.

Helman, G., Van Haren, K., Escolar, M.L., and Vanderver, A. (2015). Emerging treatments for pediatric leukodystrophies. Pediatr. Clin. North Am. *62*, 649–666.

Hubler, Z., Allimuthu, D., Bederman, I., Elitt, M.S., Madhavan, M., Allan, K.C., Shick, H.E., Garrison, E., T Karl, M., et al. (2018). Accumulation of 8,9-unsaturated sterols drives oligodendrocyte formation and remyelination. Nature https://doi.org/10.1038/s41586-018-0360-3.

Hussien, Y., Podojil, J.R., Robinson, A.P., Lee, A.S., Miller, S.D., and Popko, B. (2015). ER chaperone BiP/GRP78 is required for myelinating cell survival and provides protection during experimental autoimmune encephalomyelitis. J. Neurosci. *35*, 15921–15933.

Ikeda, M., Hossain, M.I., Zhou, L., Horie, M., Ikenaka, K., Horii, A., and Takebayashi, H. (2018). Histological detection of dynamic glial responses in the dysmyelinating Tabby-jimpy mutant brain. Anat. Sci. Int. *93*, 119–127.

Inoue, K. (2005). PLP1-related inherited dysmyelinating disorders: Pelizaeus-Merzbacher disease and spastic paraplegia type 2. Neurogenetics *6*, 1–16.

Karadottir, R., Cavelier, P., Bergersen, L.H., and Attwell, D. (2005). NMDA receptors are expressed in oligodendrocytes and activated in ischaemia. Nature *438*, 1162–1166.

Karim, S.A., Barrie, J.A., McCulloch, M.C., Montague, P., Edgar, J.M., Iden, D.L., Anderson, T.J., Nave, K.A., Griffiths, I.R., and McLaughlin, M. (2010). PLP/DM20 expression and turnover in a transgenic mouse model of Pelizaeus-Merzbacher disease. Glia *58*, 1727–1738.

Knapp, P.E., Bartlett, W.P., and Skoff, R.P. (1987). Cultured oligodendrocytes mimic in vivo phenotypic characteristics: cell shape, expression of myelin-specific antigens, and membrane production. Dev. Biol. *120*, 356–365.

Knapp, P.E., Skoff, R.P., and Redstone, D.W. (1986). Oligodendroglial cell death in jimpy mice: an explanation for the myelin deficit. J. Neurosci. *6*, 2813–2822.

Koizume, S., Takizawa, S., Fujita, K., Aida, N., Yamashita, S., Miyagi, Y., and Osaka, H. (2006). Aberrant trafficking of a proteolipid protein in a mild Pelizaeus-Merzbacher disease. Neuroscience *141*, 1861–1869.

Kramer-Albers, E.M., Gehrig-Burger, K., Thiele, C., Trotter, J., and Nave, K.A. (2006). Perturbed interactions of mutant proteolipid protein/DM20 with cholesterol and lipid rafts in oligodendroglia: implications for dysmyelination in spastic paraplegia. J. Neurosci. *26*, 11743–11752.

Krebs, C., Fernandes, H.B., Sheldon, C., Raymond, L.A., and Baimbridge, K.G. (2003). Functional NMDA receptor subtype 2B is expressed in astrocytes after ischemia in vivo and anoxia in vitro. J. Neurosci. *23*, 3364–3372.

Lager, A.M., Corradin, O., Cregg, J.M., Elitt, M.S., Shick, H.E., Clayton, B.L., Allan, K.C., Olsen, H.E., Madhavan, M., and Tesar, P.J. (2018). Rapid functional genetics of the oligodendrocyte lineage using pluripotent stem cells. Nat. Commun. https://doi.org/10.1038/41467-018-06102-7.

Lee, S., Chong, S.Y., Tuck, S.J., Corey, J.M., and Chan, J.R. (2013). A rapid and reproducible assay for modeling myelination by oligodendrocytes using engineered nanofibers. Nat. Protoc. *8*, 771–782.

Madhavan, M., Nevin, Z.S., Shick, H.E., Garrison, E., Clarkson-Paredes, C., Karl, M., Clayton, B.L.L., Factor, D.C., Allan, K.C., Barbar, L., et al. (2018). Induction of myelinating oligodendrocytes in human cortical spheroids. Nat. Methods https://doi.org/10.1038/s41592-018-0081-4.

Mallon, B.S., Shick, H.E., Kidd, G.J., and Macklin, W.B. (2002). Proteolipid promoter activity distinguishes two populations of NG2-positive cells throughout neonatal cortical development. J. Neurosci. *22*, 876–885.

Mayoral, S.R., and Chan, J.R. (2016). The environment rules: spatiotemporal regulation of oligodendrocyte differentiation. Curr. Opin. Neurobiol. *39*, 47–52.

Musner, N., Sidoli, M., Zambroni, D., Del Carro, U., Ungaro, D., D'Antonio, M., Feltri, M.L., and Wrabetz, L. (2016). Perk ablation ameliorates myelination in S63del-Charcot-Marie-Tooth 1B neuropathy. ASN Neuro. *8*. https://doi.org/10.1177/1759091416642351.

Najm, F.J., Madhavan, M., Zaremba, A., Shick, E., Karl, R.T., Factor, D.C., Miller, T.E., Nevin, Z.S., Kantor, C., Sargent, A., et al. (2015). Drug-based modulation of endogenous stem cells promotes functional remyelination in vivo. Nature *522*, 216–220.

Najm, F.J., Zaremba, A., Caprariello, A.V., Nayak, S., Freundt, E.C., Scacheri, P.C., Miller, R.H., and Tesar, P.J. (2011). Rapid and robust generation of functional oligodendrocyte progenitor cells from epiblast stem cells. Nat. Methods *8*, 957–962.

Nevin, Z.S., Factor, D.C., Karl, R.T., Douvaras, P., Laukka, J., Windrem, M.S., Goldman, S.A., Fossati, V., Hobson, G.M., and Tesar, P.J. (2017). Modeling the mutational and phenotypic landscapes of Pelizaeus-Merzbacher disease with human iPSC-derived oligodendrocytes. Am. J. Hum. Genet. *100*, 617–634.

Numasawa-Kuroiwa, Y., Okada, Y., Shibata, S., Kishi, N., Akamatsu, W., Shoji, M., Nakanishi, A., Oyama, M., Osaka, H., Inoue, K., et al. (2014). Involvement of ER stress in dysmyelination of Pelizaeus-Merzbacher disease with PLP1 missense mutations shown by iPSC-derived oligodendrocytes. Stem Cell Reports *2*, 648–661.

Numata, Y., Morimura, T., Nakamura, S., Hirano, E., Kure, S., Goto, Y.I., and Inoue, K. (2013). Depletion of molecular chaperones from

the endoplasmic reticulum and fragmentation of the Golgi apparatus associated with pathogenesis in Pelizaeus-Merzbacher disease. J. Biol. Chem. *288*, 7451–7466.

Parikh, S., Bernard, G., Leventer, R.J., van der Knaap, M.S., van Hove, J., Pizzino, A., McNeill, N.H., Helman, G., Simons, C., Schmidt, J.L., et al. (2015). A clinical approach to the diagnosis of patients with leukodystrophies and genetic leukoencephelopathies. Mol. Genet. Metab. *114*, 501–515.

Phillips, R.J. (1954). Jimpy, a new totally sexlinked gene in the house mouse. Z. Indukt. Abstamm. Vererbungsl. *86*, 322–326.

Prukop, T., Epplen, D.B., Nientiedt, T., Wichert, S.P., Fledrich, R., Stassart, R.M., Rossner, M.J., Edgar, J.M., Werner, H.B., Nave, K.A., et al. (2014). Progesterone antagonist therapy in a Pelizaeus-Merzbacher mouse model. Am. J. Hum. Genet. *94*, 533–546.

Readhead, C., Schneider, A., Griffiths, I., and Nave, K.A. (1994). Premature arrest of myelin formation in transgenic mice with increased proteolipid protein gene dosage. Neuron *12*, 583–595.

Roussel, G., Neskovic, N.M., Trifilieff, E., Artault, J.C., and Nussbaum, J.L. (1987). Arrest of proteolipid transport through the Golgi apparatus in Jimpy brain. J. Neurocytol. *16*, 195–204.

Salter, M.G., and Fern, R. (2005). NMDA receptors are expressed in developing oligodendrocyte processes and mediate injury. Nature *438*, 1167–1171.

Skoff, R.P. (1976). Myelin deficit in the Jimpy mouse may be due to cellular abnormalities in astroglia. Nature *264*, 560–562.

Somers, A., Jean, J.C., Sommer, C.A., Omari, A., Ford, C.C., Mills, J.A., Ying, L., Sommer, A.G., Jean, J.M., Smith, B.W., et al. (2010). Generation of transgene-free lung disease-specific human induced pluripotent stem cells using a single excisable lentiviral stem cell cassette. Stem Cells *28*, 1728–1740.

Sommer, C.A., Stadtfeld, M., Murphy, G.J., Hochedlinger, K., Kotton, D.N., and Mostoslavsky, G. (2009). Induced pluripotent stem cell generation using a single lentiviral stem cell cassette. Stem Cells *27*, 543–549.

Southwood, C.M., Garbern, J., Jiang, W., and Gow, A. (2002). The unfolded protein response modulates disease severity in Pelizaeus-Merzbacher disease. Neuron *36*, 585–596.

van der Knaap, M.S., and Bugiani, M. (2017). Leukodystrophies: a proposed classification system based on pathological changes and pathogenetic mechanisms. Acta Neuropathol. *134*, 351–382.

Way, S.W., Podojil, J.R., Clayton, B.L., Zaremba, A., Collins, T.L., Kunjamma, R.B., Robinson, A.P., Brugarolas, P., Miller, R.H., Miller, S.D., et al. (2015). Pharmaceutical integrated stress response enhancement protects oligodendrocytes and provides a potential multiple sclerosis therapeutic. Nat. Commun. *6*, 6532.

Williams, W.C., 2nd, and Gard, A.L. (1997). In vitro death of jimpy oligodendrocytes: correlation with onset of DM-20/PLP expression and resistance to oligodendrogliotrophic factors. J. Neurosci. Res. *50*, 177–189.

Yool, D.A., Edgar, J.M., Montague, P., and Malcolm, S. (2000). The proteolipid protein gene and myelin disorders in man and animal models. Hum. Mol. Genet. *9*, 987–992.

Zhang, J.H., Chung, T.D., and Oldenburg, K.R. (1999). A simple statistical parameter for use in evaluation and validation of high throughput screening assays. J. Biomol. Screen *4*, 67–73.

Zhang, Y., Chen, K., Sloan, S.A., Bennett, M.L., Scholze, A.R., O'Keeffe, S., Phatnani, H.P., Guarnieri, P., Caneda, C., Ruderisch, N., et al. (2014). An RNA-sequencing transcriptome and splicing database of glia, neurons, and vascular cells of the cerebral cortex. J. Neurosci. *34*, 11929–11947.

Stem Cell Reports, Volume *11*

## Supplemental Information

# Chemical Screening Identifies Enhancers of Mutant Oligodendrocyte Survival and Unmasks a Distinct Pathological Phase in Pelizaeus-Merzbacher Disease

**Matthew S. Elitt, H. Elizabeth Shick, Mayur Madhavan, Kevin C. Allan, Benjamin L.L. Clayton, Chen Weng, Tyler E. Miller, Daniel C. Factor, Lilianne Barbar, Baraa S. Nawash, Zachary S. Nevin, Angela M. Lager, Yan Li, Fulai Jin, Drew J. Adams, and Paul J. Tesar**

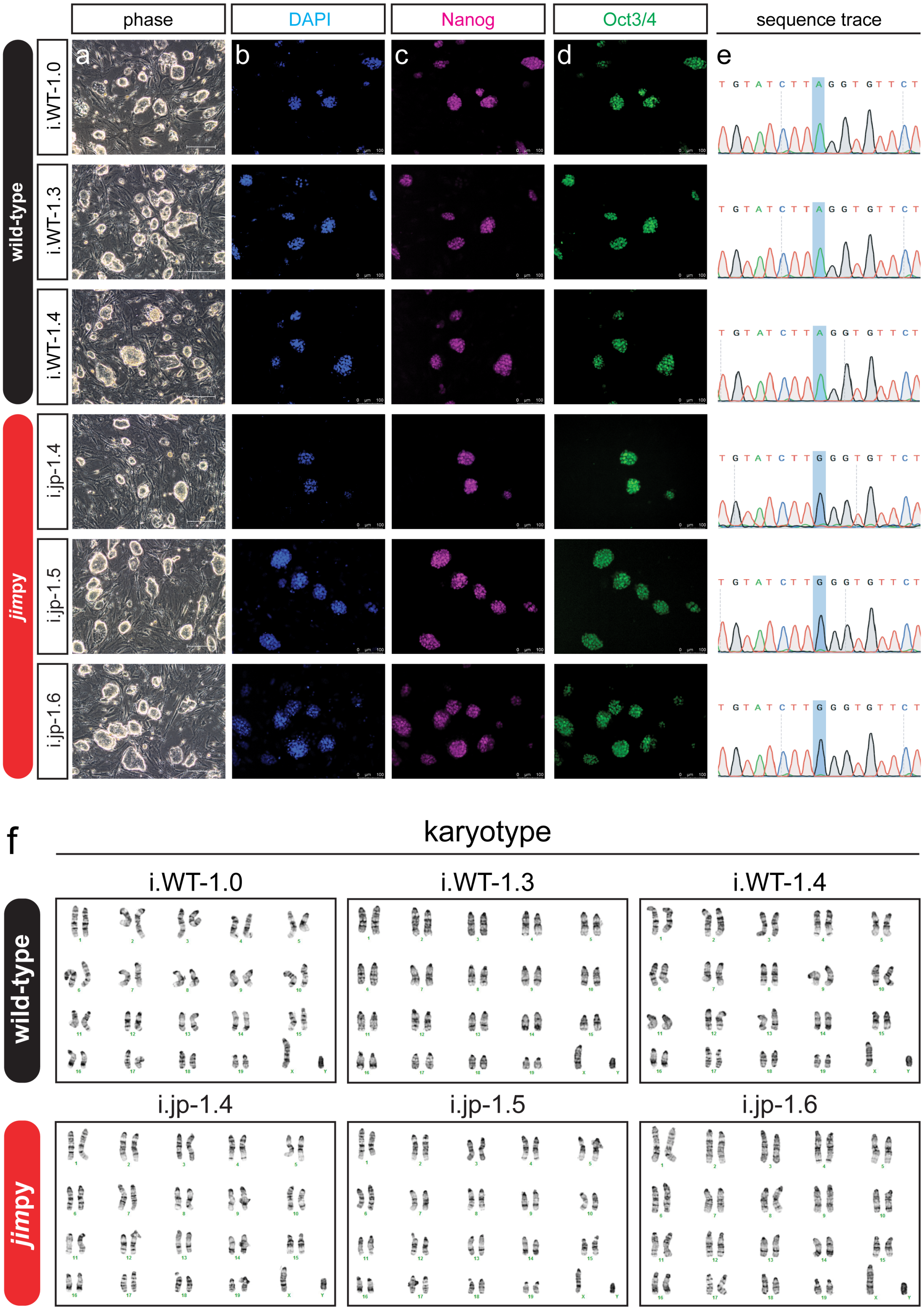
phase
DAPI
Nanog
Oct3/4
sequence trace
a
b
c
d
e
wild-type
jimpy
i.WT-1.0
i.WT-1.3
i.WT-1.4
i.jp-1.4
i.jp-1.5
i.jp-1.6
T G T A T C T T A G G T G T T C T
T G T A T C T T G G G T G T T C T
f
karyotype
i.WT-1.0
i.WT-1.3
i.WT-1.4
i.jp-1.4
i.jp-1.5
i.jp-1.6

**Figure S1. Wild-type and *jimpy* iPSCs exhibit grossly normal morphology and karyotypes, and express canonical pluripotency markers. Related to Figure 1**

**(a)** Representative phase contrast images of wild-type and *jimpy* iPSC lines. Scale bar, 200µm. **(a-d)** Representative immunocytochemistry images of wild-type and *jimpy* iPSC lines showing **(b)** DAPI, **(c)** Nanog, and **(d)** Oct3/4 staining. Scale bar, 100µm. **(e)** 5' to 3' Sanger sequencing traces showing *jimpy* mutation status (highlighted with blue bar, A=wild-type and G=*jimpy*) for wild-type and *jimpy* iPSC lines. **(f)** Metaphase spreads for wild-type and *jimpy* iPSC lines displaying grossly normal karyotypes. Three lines exhibited loss or gain of the Y chromosome, a common occurrence in mouse iPSC lines, in a subset of cells (2, 5, 6, and 2 out of 20 for i.wt-1.0, i.wt-1.4 i.jp-1.4, i.jp-1.5, respectively).

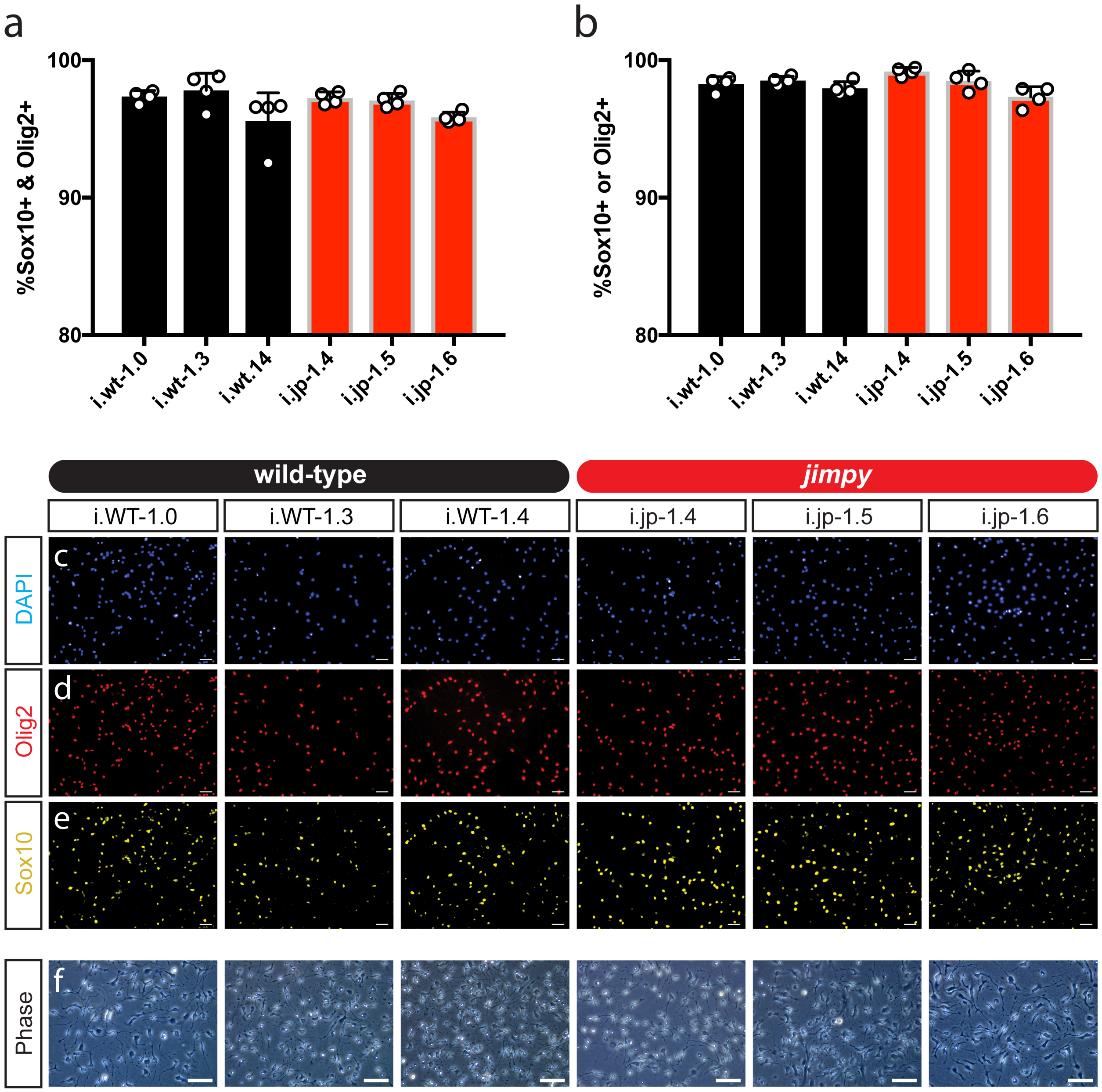


**Figure S2. Wild-type and *jimpy* iPSC-derived OPCs lines are highly pure based on canonical OPC markers expression. Related to Figure 1**

**(a)** Percentage of double Sox10+ & Olig2+ OPCs in wild-type (black) and *jimpy* (red) cell lines. **(b)** Percentage of Sox10+ or Olig2+ OPCs in wild-type (black) and *jimpy* (red) cell lines. **(c-e)** Representative immunocytochemistry images of wild-type and *jimpy* OPCs lines showing **(c)** DAPI, **(d)** Olig2, and **(e)** Sox10 staining. Scale bar, 50µm. **(f)** Representative phase contrast images of wild-type and *jimpy* OPCs lines. Scale bar, 100µm**.** Error bars represent mean ± SD. n=4 replicate wells quantified for each cell line. Each replicate value represented by a white circle.

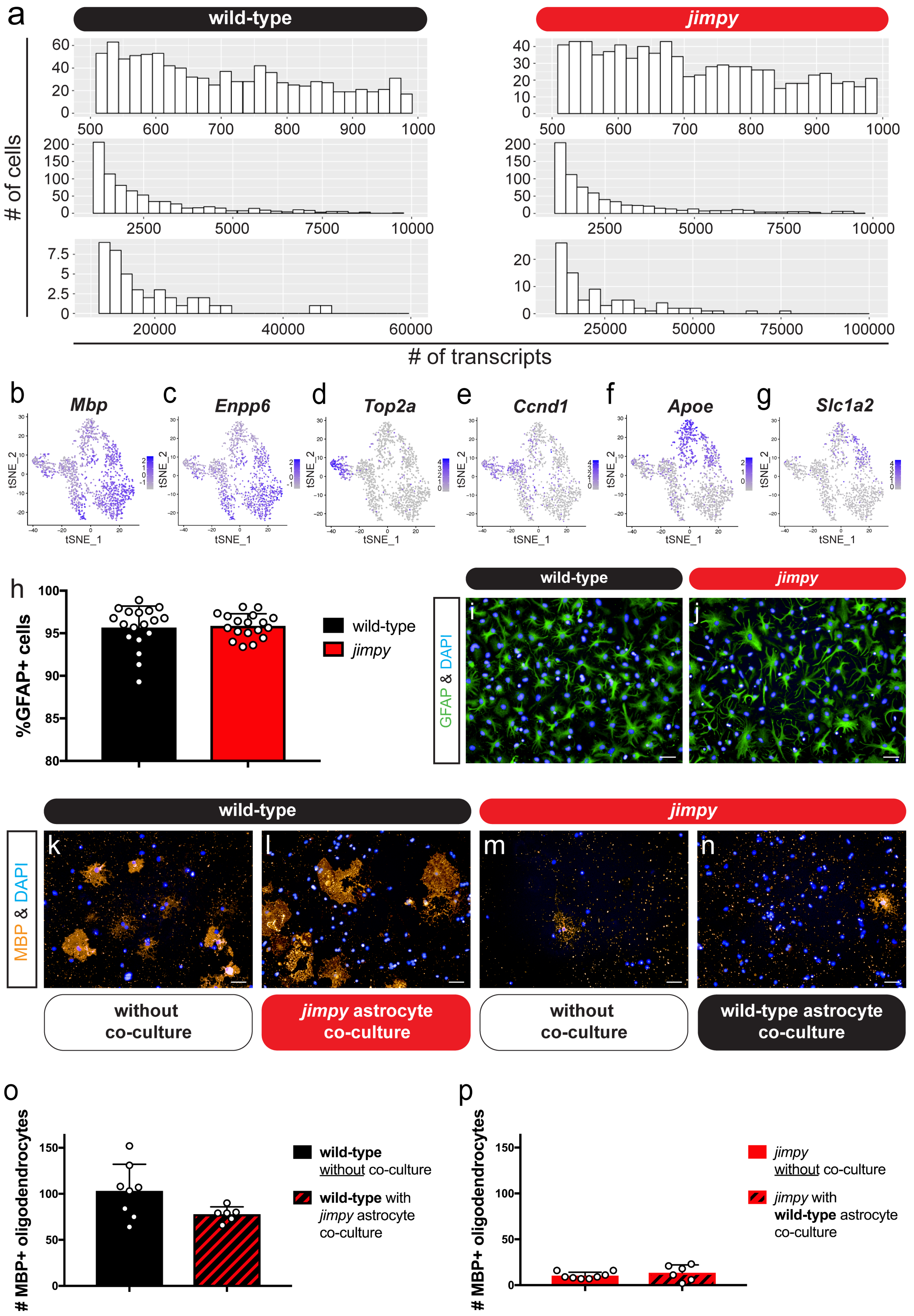
a
wild-type
jimpy
# of cells
# of transcripts
b Mbp
c Enpp6
d Top2a
e Ccnd1
f Apoe
g Slc1a2
tSNE_1
tSNE_2
h
%GFAP+ cells
wild-type
jimpy
GFAP & DAPI
i
j
MBP & DAPI
k
l
m
n
without co-culture
jimpy astrocyte co-culture
without co-culture
wild-type astrocyte co-culture
o
# MBP+ oligodendrocytes
wild-type without co-culture
wild-type with jimpy astrocyte co-culture
p
# MBP+ oligodendrocytes
jimpy without co-culture
jimpy with wild-type astrocyte co-culture

**Figure S3. scRNA-seq quality control metrics and astrocyte characterization. Related to Figure 2**
**(a)** Histogram of individual transcripts sequenced per cell at day one of oligodendrocyte differentiation for wild-type and *jimpy* cultures. **(b-g)** tSNE plots of both wild-type and *jimpy* cells at day one of oligodendrocyte differentiation showing normalized expression levels of **(b)** *Mbp* or **(e)** *Enpp6*, **(c)** *Top2a* or **(f)** *Ccnd1*, and **(d)** *Apoe* or **(g)** *Slc1a2* to inform identification of nascent oligodendrocytes, OPCs, and nascent astrocytes, respectively, after unsupervised clustering. **(h)** Quantification of %GFAP+ cells after 3 day astrocyte differentiation of iPSC-derived wild-type and *jimpy* OPCs. n=18 replicate wells per genotype. **(i-j)** Representative immunocytochemistry images after 3 day astrocyte differentiation of iPSC-derived **(i)** wild-type and **(j)** *jimpy* OPCs showing GFAP+ astrocytes (green) and total DAPI+ cells (blue). **(k-n)** Representative immunocytochemistry images after 3 day oligodendrocyte differentiation of iPSC-derived wild-type and *jimpy* OPCs showing MBP+ oligodendrocytes (orange) and total DAPI+ cells (blue) in wild-type cultures **(k)** alone and **(l)** with *jimpy* astrocyte co-culture and in *jimpy* cultures **(m)** alone and **(n)** with wild-type astrocyte co-culture. **(o)** Quantification of MBP+ oligodendrocytes after 3 day oligodendrocyte differentiation of iPSC-derived wild-type OPCs with (n=6 replicate wells) and without (n=8 replicate wells) *jimpy* astrocyte co-culture. **(p)** Quantification of MBP+ oligodendrocytes after 3 day oligodendrocyte differentiation of iPSC-derived *jimpy* OPCs with (n=6 replicate wells) and without (n=8 replicate wells) wild-type astrocyte co-culture. Error bars represent mean ± SD. Each replicate value represented by a white circle.

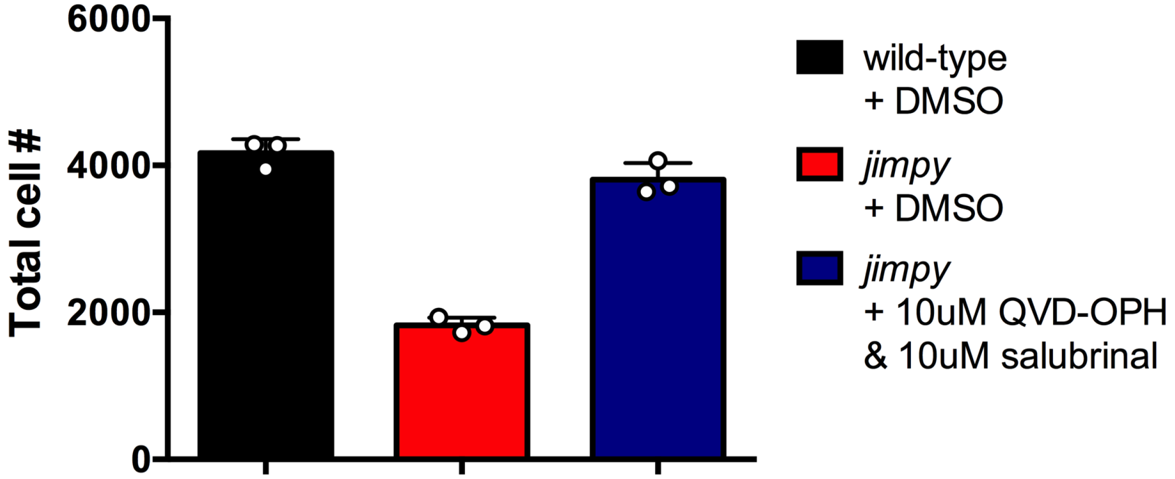


**Figure S4. Q-VD-OPh and salubrinal, in combination, restore *jimpy* cell number back to wild-type levels. Related to Figure 3.**

Quantification of total cell number after 3 day oligodendrocyte differentiation of wild-type OPCs treated with DMSO vehicle, and *jimpy* OPCs treated with DMSO vehicle or 10µM Q-VD-OPh & 10µM salubrinal. n=3 replicate wells per treatment. Error bars represent mean ± SD. Each replicate value represented by a white circle.

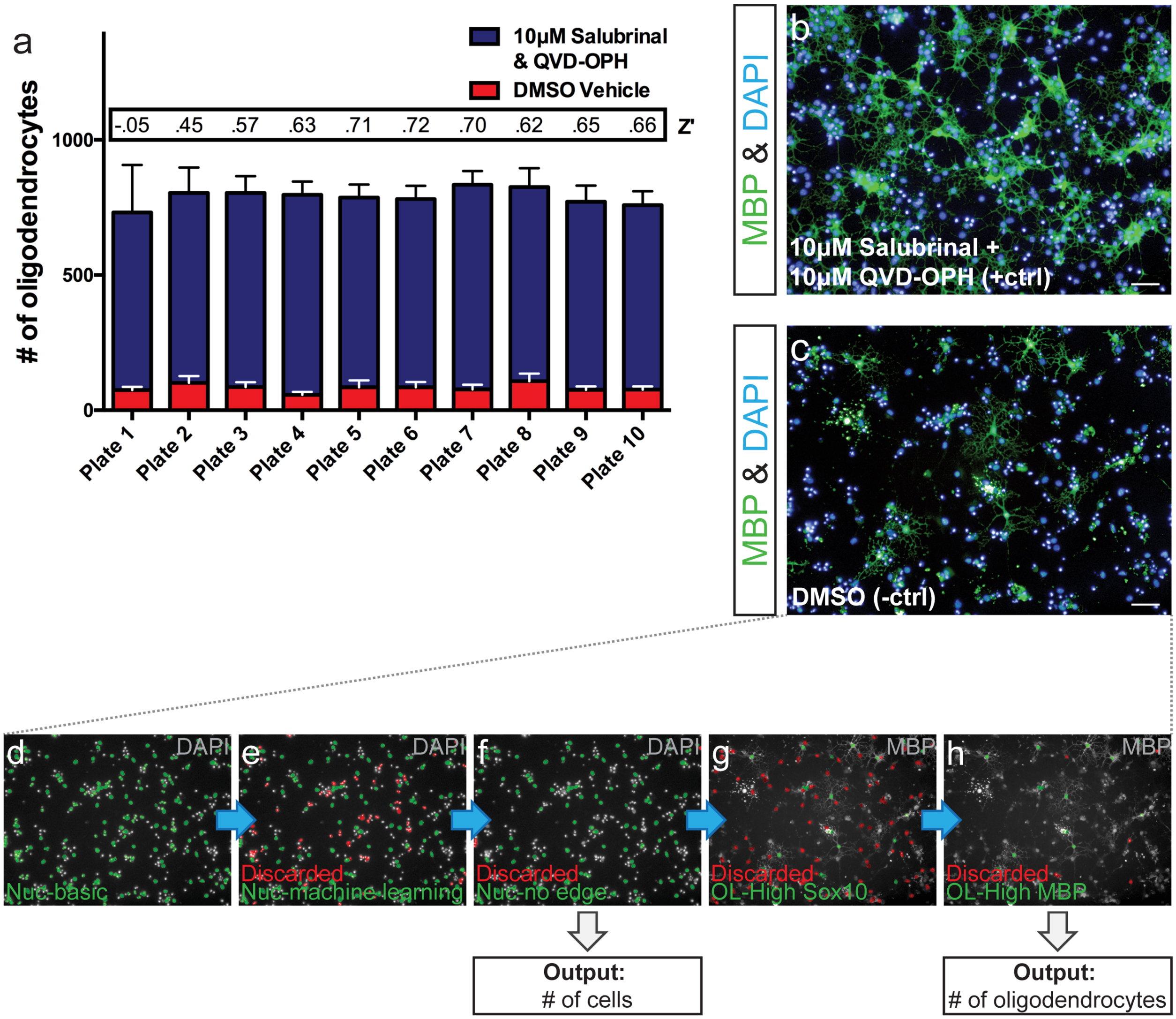


**Figure S5. Primary screening controls and automated image analysis display robust performance. Related to Figure 4**

**(a)** Primary screen negative control (DMSO vehicle) and positive control (10μM Q-VD-OPh and 10μM salubrinal) metrics on a per plate basis with corresponding Z' factor for oligodendrocyte number. n=16 replicate wells per control, per screening plate. Error bars represent mean ± SD. **(b and c)** Representative immunocytochemistry images of primary screen controls for **(b)** 10μM Q-VD-OPh and 10μM salubrinal and **(c)** DMSO vehicle treated *jimpy* OPCs after oligodendrocyte differentiation showing oligodendrocytes (MBP, green) and total cells (DAPI, blue). Scale bar, 50μm. **(d to h)** Image analysis pipeline**. (d)** Single-field capture of putative DAPI+ nuclei (green) with a basic nuclei finding analysis, further refined with **(e)** machine learning to discard pyknotic nuclei (red) and **(f)** remove nuclei contacting the image border (red) for a final output of bona-fide DAPI+ nuclei (green), defined as total cell number for the analyzed field. **(g)** DAPI+ nuclei with high levels of Sox10 expression (green) defined a putative oligodendrocyte population which was further refined using **(h)** high MBP expression (green) to define the oligodendrocyte number for the analyzed field.

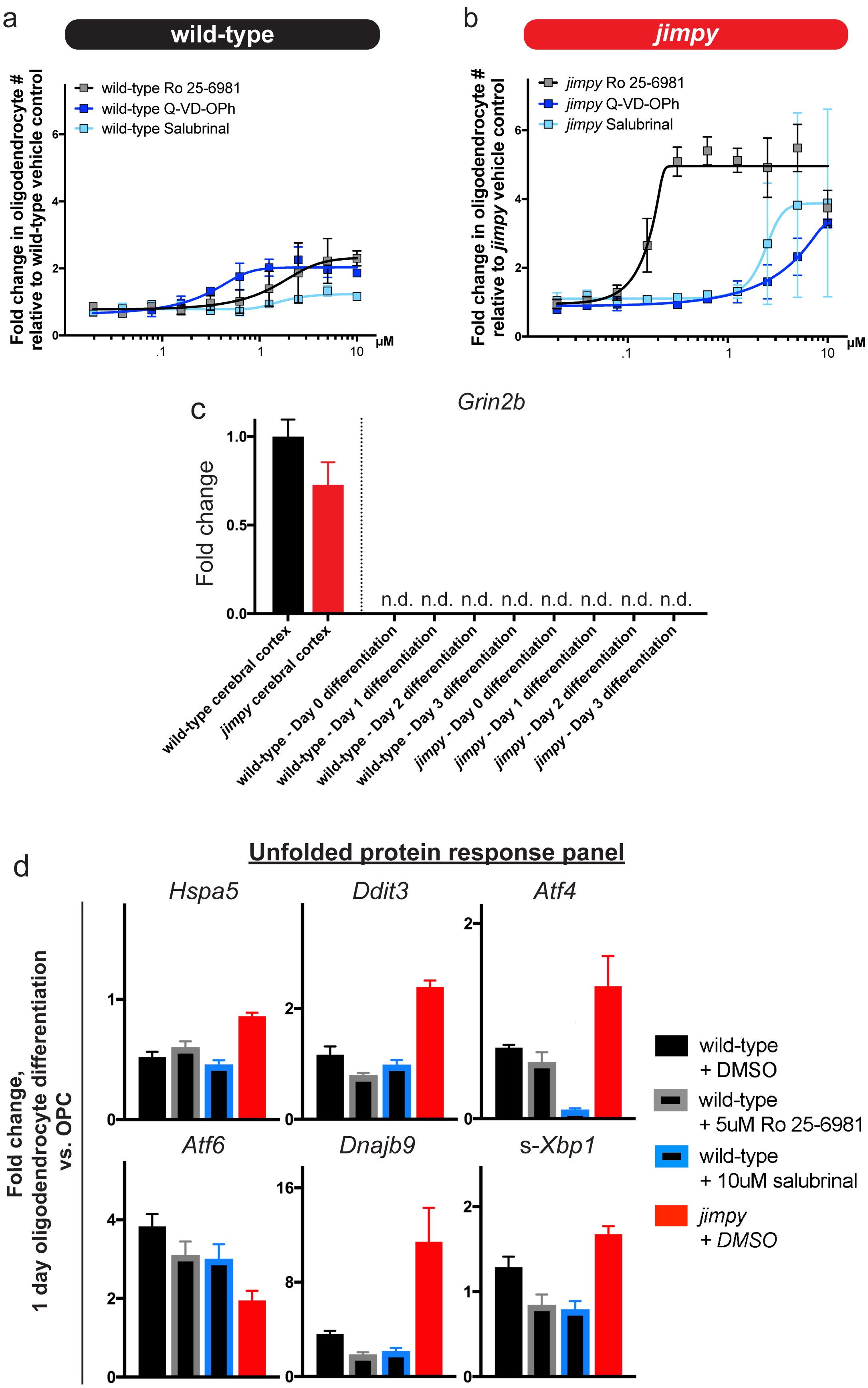

a
wild-type
wild-type Ro 25-6981
wild-type Q-VD-OPh
wild-type Salubrinal
Fold change in oligodendrocyte # relative to wild-type vehicle control
μM
b
jimpy
jimpy Ro 25-6981
jimpy Q-VD-OPh
jimpy Salubrinal
Fold change in oligodendrocyte # relative to jimpy vehicle control
μM
c
Grin2b
Fold change
n.d.
wild-type cerebral cortex
jimpy cerebral cortex
wild-type - Day 0 differentiation
wild-type - Day 1 differentiation
wild-type - Day 2 differentiation
wild-type - Day 3 differentiation
jimpy - Day 0 differentiation
jimpy - Day 1 differentiation
jimpy - Day 2 differentiation
jimpy - Day 3 differentiation
d
Unfolded protein response panel
Hspa5
Ddit3
Atf4
Atf6
Dnajb9
s-Xbp1
Fold change, 1 day oligodendrocyte differentiation vs. OPC
wild-type + DMSO
wild-type + 5uM Ro 25-6981
wild-type + 10uM salubrinal
jimpy + DMSO

**Figure S6. Evaluation of *jimpy* modulators during oligodendrocyte differentiation in wild-type and *Grin2b* expression assessment. Related to Figure 5**

**(a)** Fold change in MBP+ oligodendrocytes (relative to DMSO vehicle control) in 10-point (10µM-20nM) dose response testing for Ro 25-6981, salubrinal, and Q-VD-OPh after 3 day oligodendrocyte differentiation of iPSC-derived wild-type OPCs. n=3 replicate wells per compound treatment. n=16 replicate wells for vehicle control. **(b)** Fold change in MBP+ oligodendrocytes (relative to DMSO vehicle control) in 10-point (10µM-20nM) dose response testing for Ro 25-6981, salubrinal, and Q-VD-OPh after 3 day oligodendrocyte differentiation of iPSC-derived *jimpy* OPCs. n=3 replicate wells per compound treatment. n=16 replicate wells for vehicle control. **(c)** RT-qPCR of *Grin2b* for cerebral cortex harvested from postnatal day 19 wild-type (black) and *jimpy* (red) mice along with differentiating wild-type and *jimpy* OPCs collected at day 0, 1, 2, and 3 of oligodendrocyte differentiation. n=3 technical replicates for wild-type day 0 differentiation. n=4 technical replicates for all other samples. Samples where *Grin2b* was not detected (n.d.) lacked amplification through 36 thermocycles. **(d)** RT-qPCR of *Dnajb9*, *s-Xbp1*, *Hspa5*, *Ddit3*, *Atf4*, *Atf6* for wild-type and *jimpy* OPCs collected at day 1 of oligodendrocyte differentiation with indicated treatment. Vehicle treated controls same as Figure 5. *Atf6* n=3 technical replicates per sample. For all other probes n=4 technical replicates per sample. Error bars represent mean ± SD.

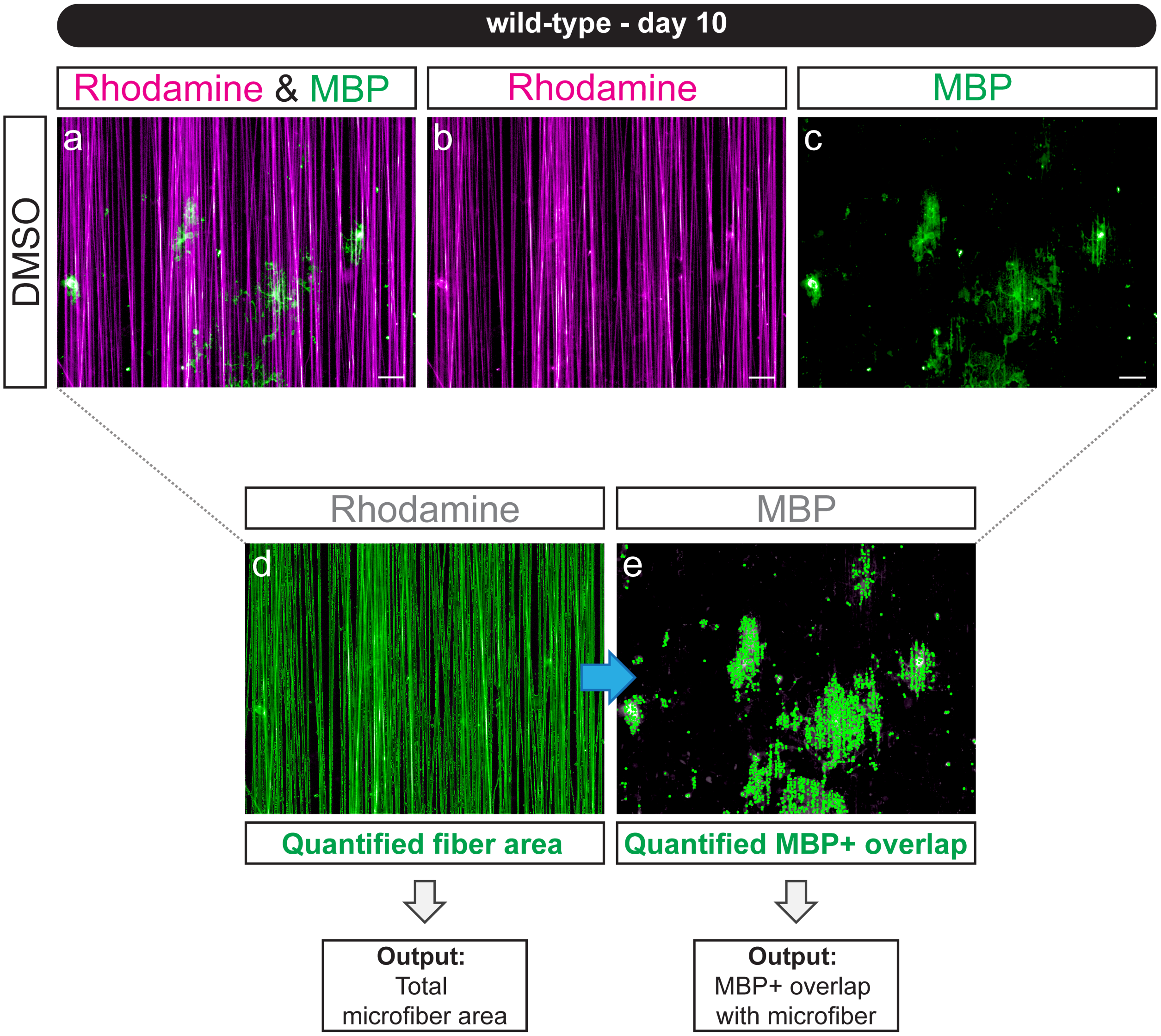


**Figure S7. Automated image analysis accurately identifies myelinating oligodendrocytes. Related to Figure 7**
**(a-c)** Single-field capture, representative immunocytochemistry input images are shown with the **(a)** composite of MBP+ myelinating oligodendrocytes (green) with rhodamine+ microfibers (purple) as well as single channel images of **(b)** rhodamine+ microfibers (purple) and **(c)** MBP+ myelinating oligodendrocytes (green) at day 10 for wild-type cultures treated with DMSO vehicle. **(d)** Detection of regions of high rhodamine+ (grey) intensity to define the total microfiber area (green). **(e)** Detection of regions of high MBP+ (grey) intensity to quantify MBP+ overlap with microfibers (green).

| Compound Name | 1° Screen Rank | Confirm in distinct *jimpy* lines (#) | 10 uM | 5 uM | 2.5 uM | 1.25 uM | 625 nM | 313 nM | 156 nM | 78 nM | Max. value |
|---|---|---|---|---|---|---|---|---|---|---|---|
| Ro 25-6981 | 45 | 2 | 28.6 | 48.4 | 50.7 | 52.0 | 55.4 | 53.3 | 29.2 | 23.6 | **55.4** |
| Vesamicol | 29 | 2 | 43.2 | 43.4 | 30.3 | 9.1 | 10.4 | 6.1 | 3.0 | 3.3 | **43.4** |
| Medroxyprogesterone | 32 | 2 | 42.9 | 10.9 | 8.2 | 5.9 | 2.5 | -0.9 | -1.3 | 2.1 | **42.9** |
| Bifonazole | 2 | 2 | 16.9 | 29.4 | 41.7 | 26.1 | 11.9 | 9.3 | 7.6 | 5.1 | **41.7** |
| Ketoconazole | 51 | 1 | 32.1 | 35.9 | 41.3 | 12.0 | 26.6 | 6.3 | 17.2 | 9.0 | **41.3** |
| SB408124 | 22 | 2 | 21.2 | 20.5 | 26.5 | 34.6 | 40.0 | 29.5 | 8.2 | 1.1 | **40.0** |
| Raltegravir | 1 | 2 | 38.2 | 34.6 | 29.8 | 14.9 | 4.0 | 4.2 | 1.4 | 1.6 | **38.2** |
| Estrone | 7 | 2 | 36.4 | 31.0 | 37.4 | 34.9 | 35.0 | 23.0 | 6.2 | -0.2 | **37.4** |
| Fenspiride | 15 | 1 | 36.6 | 17.5 | 15.1 | 11.5 | 4.2 | 6.2 | 4.9 | 6.3 | **36.6** |
| EPZ005687 | 25 | 2 | 20.2 | 21.9 | 30.8 | 34.2 | 21.4 | 14.5 | 8.8 | 1.7 | **34.2** |
| Climbazole | 6 | 2 | 32.2 | 14.4 | 8.8 | 5.2 | 1.5 | 0.5 | -0.1 | 2.0 | **32.2** |
| Ziprasidone | 13 | 2 | 19.0 | 31.8 | 20.6 | 10.5 | 3.3 | 1.4 | 2.8 | 0.8 | **31.8** |
| Anisotropine | 8 | 2 | 31.5 | 22.4 | 12.4 | 9.4 | 4.2 | 4.2 | 1.7 | 2.9 | **31.5** |
| Ifenprodil | 37 | 1 | 4.6 | 15.6 | 24.8 | 31.2 | 31.2 | 29.4 | 23.0 | 13.6 | **31.2** |
| Ro 8-4304 | 42 | 2 | 31.1 | 10.9 | -0.1 | 0.7 | -1.2 | -1.8 | -0.6 | -0.1 | **31.1** |
| U-101958 | 23 | 2 | 29.2 | 20.7 | 11.6 | 6.6 | 4.2 | 0.1 | 1.0 | 0.0 | **29.2** |
| Amorolfine | 50 | 2 | 10.3 | 19.6 | 22.5 | 22.8 | 25.9 | 26.9 | 22.9 | 22.9 | **26.9** |
| JNJ-40418677 | 10 | 2 | 24.0 | 6.0 | 5.6 | -0.2 | 2.5 | -0.2 | 0.8 | 2.0 | **24.0** |
| SANT-1 | 54 | 1 | 20.9 | 19.7 | 11.2 | 11.7 | 4.5 | 5.0 | 3.2 | 4.7 | **20.9** |
| Q-VD-Oph & salubrinal | Positive control | | 20.8 | | | | | | | | **20.8** |
| m-Iodobenzylguanidine | 18 | 2 | 19.5 | 20.3 | 13.7 | 14.5 | 9.2 | 3.6 | 6.1 | 1.7 | **20.3** |
| Luliconazole | 53 | 2 | 20.1 | 10.7 | 4.5 | 3.8 | 2.6 | 0.8 | -0.2 | -1.1 | **20.1** |
| Mestranol | 59 | 2 | 18.2 | 11.7 | 7.1 | 5.3 | 4.1 | 4.5 | 1.5 | 0.9 | **18.2** |
| Ondansetron | 44 | 2 | 18.2 | 7.6 | 12.5 | 7.9 | 3.8 | 4.1 | 2.5 | 4.5 | **18.2** |
| Dofetilide | 60 | 2 | 17.6 | 6.8 | 6.3 | 0.3 | 0.4 | 3.8 | -0.5 | 5.8 | **17.6** |
| Megestrol | 33 | 2 | 17.2 | 5.9 | 2.2 | 2.4 | 1.6 | 3.6 | 2.5 | 3.6 | **17.2** |
| Vitamin D2 | 16 | 1 | 17.1 | 12.4 | 5.9 | 1.5 | 0.8 | 4.2 | 3.0 | 2.2 | **17.1** |
| Clobetasol | 43 | 1 | 17.0 | 10.9 | 16.5 | 8.4 | 3.7 | 8.6 | 2.4 | 3.3 | **17.0** |
| Halobetasol | 11 | 2 | 12.9 | 10.3 | 12.8 | 15.7 | 10.3 | 4.0 | 13.2 | 7.8 | **15.7** |
| Idebenone | 63 | 1 | 13.6 | 9.0 | 3.3 | 1.7 | 2.1 | 2.4 | -0.5 | 0.7 | **13.6** |
| Oxybutynin | 30 | 2 | 13.2 | 10.7 | 11.6 | 7.3 | 6.2 | 2.1 | 2.8 | 1.1 | **13.2** |
| Budesonide | 3 | 1 | 12.2 | 10.0 | 5.4 | 5.0 | 6.3 | 6.1 | 6.1 | 5.3 | **12.2** |
| Wnt-C59 | 28 | 1 | 10.8 | 3.4 | 1.4 | 2.1 | 2.3 | 1.7 | -0.2 | -0.2 | **10.8** |
| Palmatine | 61 | 1 | 10.5 | 6.2 | 7.4 | 6.7 | 3.6 | 1.7 | 3.0 | 3.2 | **10.5** |
| Dexlansoprazole | 14 | 2 | 10.4 | 6.7 | 4.3 | 2.6 | -0.1 | 0.9 | 2.4 | 0.1 | **10.4** |
| Malotilate | 24 | 1 | 9.8 | 2.0 | 2.5 | 0.5 | -0.6 | 1.3 | 0.8 | 0.1 | **9.8** |
| GW3965 | 21 | 2 | 9.4 | 8.2 | 5.1 | 1.5 | 2.6 | 0.4 | 0.8 | -0.2 | **9.4** |
| Mosapride | 9 | 2 | 9.2 | 2.3 | 6.6 | 5.0 | 1.8 | 1.1 | 3.0 | 1.1 | **9.2** |
| SB216763 | 34 | 2 | 0.5 | 5.0 | 8.5 | 4.1 | 2.9 | 1.5 | 2.9 | 1.9 | **8.5** |
| Butenafine | 49 | 1 | 8.1 | 5.6 | 2.6 | 2.5 | 1.1 | 1.4 | -1.0 | 0.6 | **8.1** |
| Betamethasone | 36 | 2 | 7.9 | 4.5 | 2.4 | 5.9 | 2.9 | 3.0 | 2.6 | 3.3 | **7.9** |
| Diphemanil | 35 | 1 | 7.9 | 2.9 | 2.2 | 0.5 | -1.4 | 1.0 | 0.2 | -0.2 | **7.9** |
| Enzalutamide | 12 | 1 | 7.8 | 5.7 | 4.7 | 5.4 | 4.5 | 2.5 | 4.3 | 4.9 | **7.8** |

| | | | | | | | | | | | |
|---|---|---|---|---|---|---|---|---|---|---|---|
| Q-VD-OPh | 4 | 2 | 7.5 | 6.1 | -3.4 | 1.6 | 0.4 | 0.1 | -0.1 | -0.6 | **7.5** |
| AGI-6780 | 55 | 1 | 6.2 | 3.6 | 2.5 | 0.7 | 1.7 | -0.1 | 1.0 | -0.6 | **6.2** |
| Isoconazole | 17 | 2 | 25.9 | 18.2 | 14.0 | 9.9 | 10.4 | 5.9 | 4.2 | 4.1 | **6.2** |
| AZD7545 | 52 | 2 | 6.0 | 3.7 | 2.1 | 4.4 | 0.6 | -0.2 | 0.1 | 2.9 | **6.0** |
| Salubrinal | 57 | 1 | 3.3 | 5.0 | 6.0 | 5.2 | 4.8 | 2.3 | 3.2 | 0.4 | **6.0** |
| Emricasan | 40 | 1 | 5.5 | -1.7 | -0.4 | 2.0 | 3.3 | 0.9 | 1.2 | 0.0 | **5.5** |
| OSI-930 | 46 | 1 | -1.4 | 5.0 | -0.5 | 1.7 | 1.8 | -0.1 | -1.4 | 0.5 | **5.0** |
| Tubastatin A | 47 | 1 | 4.9 | 1.5 | 0.9 | 3.4 | 5.0 | 3.7 | 2.1 | 0.3 | **5.0** |
| IU1 | 36 | 1 | 4.7 | 2.2 | 1.0 | 1.7 | -0.2 | 0.5 | -1.3 | -0.4 | **4.7** |
| Progesterone | 27 | 1 | 4.5 | 1.8 | 1.5 | 1.1 | 1.6 | 0.0 | -0.1 | -0.5 | **4.5** |
| Pyrimethamine | 5 | 1 | 2.8 | 4.2 | 1.6 | 3.4 | 3.8 | 2.3 | 4.3 | 3.0 | **4.3** |
| Ouabain | 20 | 1 | 0.0 | 1.6 | 1.1 | 3.0 | -0.3 | 0.0 | 0.7 | 4.1 | **4.1** |
| Allylthiourea | 48 | 0 | | | | | | | | | |
| Biperiden | 39 | 0 | | | | | | | | | |
| Cilostazol | 64 | 0 | | | | | | | | | |
| Cyproterone | 31 | 0 | | | | | | | | | |
| Fluorometholone | 41 | 0 | | | | | | | | | |
| Lubiprostone | 26 | 0 | | | | | | | | | |
| Praziquantel | 19 | 0 | | | | | | | | | |
| Sorafenib | 58 | 0 | | | | | | | | | |
| Ticlopidine | 56 | 0 | | | | | | | | | |
| ZM 336372 | 62 | 0 | | | | | | | | | |

**Table S5. Hit confirmation and lead compound selection. Related to Figure 4**
64 compound hits from primary (>3000) bioactive screen were tested in two additional iPSC-derived *jimpy* OPC lines at 10µM, single replicate per cell line. Hits that met the primary screening hit selection criterion in at least one cell line highlighted in black and considered confirmed compounds. Compounds that failed confirmation testing are highlighted in red. Confirmed hits were tested in the iPSC-derived *jimpy* OPC line used in the primary screen over an eight-point dose response (from 10µM to 78nM), with a single replicate well per compound dose. Values indicate the number of SDs above the mean of the negative control (DMSO vehicle) for quantified MBP+ oligodendrocytes. Values that met the MBP+ oligodendrocyte primary screening criterion are indicated by green. Compounds were then rank ordered based on their maximal responses, highlighting the lead compound Ro 25-6981 (indicated in grey).

| Compound Name | 10uM | 5uM | 2.5uM | 1.25 uM | 625 nM | 313 nM | 156 nM | 78nM | 39nM | 20nM |
|---|---|---|---|---|---|---|---|---|---|---|
| Eliprodil | 1.3 | 15.8 | 9.3 | 1.0 | 0.7 | -0.2 | 0.0 | -0.5 | 0.1 | -0.3 |
| Ifenprodil | 28.2 | 5.3 | 18.4 | 10.7 | 6.8 | 0.7 | 0.6 | 0.7 | 0.0 | 0.3 |
| Lubeluzole | 2.0 | -0.5 | -0.6 | 0.4 | -0.3 | -0.5 | 0.1 | 0.1 | 0.1 | -0.2 |
| Rislenemdaz | -0.3 | 0.0 | 1.5 | 1.5 | -0.3 | -0.9 | -0.2 | -0.6 | 0.4 | 0.0 |
| Ro 8-4304 | 3.8 | 0.0 | -0.2 | 1.2 | 0.0 | -0.9 | -0.3 | -0.8 | 0.1 | 0.1 |
| Ro 25-6981 | 22.9 | 40.4 | 34.0 | 14.2 | 3.3 | 0.7 | -0.2 | -0.8 | -0.2 | -0.3 |
| TCS 46b | 22.9 | 6.4 | 0.6 | -0.5 | -0.8 | -0.5 | -0.2 | -0.9 | 0.0 | -0.5 |
| TCN 237 | 2.3 | -0.6 | 0.4 | 0.0 | 0.3 | 0.6 | 0.1 | 0.9 | 0.6 | -0.2 |
| (±)-2-Amino-3-phosphonopropionic acid | 1.8 | 0.7 | 1.2 | -0.5 | 0.0 | 0.0 | 0.6 | 2.4 | 0.7 | 2.3 |
| (±)-2-Amino-4-phosphonobutyric acid | 2.6 | 1.3 | 0.9 | 1.8 | 0.6 | -0.3 | 0.0 | -0.6 | -0.2 | -0.5 |
| (±)-2-Amino-5-phosphonopentanoic acid | -0.5 | 1.6 | 0.4 | 0.9 | 2.0 | -0.3 | -0.6 | 1.5 | 0.0 | -0.3 |
| D(-)-2-Amino-5-phosphonopentanoic acid | 0.7 | 0.0 | 1.2 | 0.1 | 0.9 | 0.1 | -0.5 | -0.3 | -0.5 | -0.5 |
| Arcaine | 1.0 | 0.7 | 0.0 | 0.1 | -0.2 | -0.5 | -0.3 | -0.3 | 0.0 | -0.5 |
| CNS-1102 | -0.9 | -0.6 | -0.6 | -0.2 | -0.2 | -0.5 | -0.6 | -0.8 | -0.3 | 0.1 |
| (±)-CPP | 0.6 | 0.3 | -0.2 | 1.8 | 0.4 | 1.3 | 0.3 | 1.0 | -0.3 | 0.4 |
| Dextromethorphan | -0.5 | -0.2 | -0.6 | -0.6 | -0.3 | -0.3 | 0.0 | -0.3 | -0.3 | -0.5 |
| 5,7-Dichlorokynurenic acid | -0.5 | 1.2 | 0.4 | 0.0 | 0.4 | 0.7 | 0.1 | 0.0 | -0.5 | 0.3 |
| 5-Fluoroindole-2-carboxylic acid | 0.3 | -0.6 | 0.1 | 1.2 | -0.2 | 0.3 | 1.0 | -0.6 | 0.1 | -0.5 |
| Flupirtine | 0.6 | -0.2 | 0.9 | 0.3 | -0.2 | -0.3 | 0.1 | -0.3 | -0.6 | -0.2 |
| (±)-HA-966 | -0.5 | -0.3 | -0.5 | 0.1 | 1.0 | 0.3 | -0.3 | -0.5 | -0.9 | -0.2 |
| L-701,324 | -0.5 | 0.4 | 0.4 | 1.3 | 1.0 | -0.2 | -0.2 | -0.5 | 0.3 | -0.5 |
| Kynurenic acid | 0.4 | 0.9 | 0.0 | 0.1 | 0.7 | 0.0 | 0.0 | -0.2 | 1.0 | 0.4 |
| Memantine | -0.2 | -0.6 | -0.3 | 0.3 | 0.0 | 0.6 | 2.3 | 0.0 | 1.2 | 0.0 |
| MK-801 | -0.9 | 0.9 | 2.3 | -0.3 | 0.3 | 1.5 | 2.0 | 0.1 | 1.3 | 0.7 |
| O-Phospho-L-serine | 0.0 | 0.1 | 0.3 | 0.3 | 0.7 | 0.6 | 0.4 | 0.6 | -0.2 | 1.6 |

| | | | | | | | | | | |
|---|---|---|---|---|---|---|---|---|---|---|
| Pentamidine | -0.9 | -0.8 | 1.0 | 0.0 | -0.6 | -0.2 | -0.9 | 0.0 | 0.7 | -0.3 |
| Quinolinic acid | 1.3 | -0.2 | 0.0 | -0.5 | 0.4 | 0.1 | 0.4 | 0.0 | 0.7 | -0.6 |
| Spermidine | 2.6 | 0.9 | 0.7 | 0.7 | 0.0 | 0.4 | 1.5 | 0.3 | 1.2 | -0.2 |
| Spermine | 1.5 | 0.7 | 1.3 | 0.6 | 0.1 | -0.2 | 2.0 | 0.3 | 0.1 | 0.6 |

**Table S6. 10-point dose testing of 29 NMDA modulators on differentiating *jimpy* OPCs. Related to Figure 5**
Effects of 29 NMDA receptor modulators (from 10µM to 19.5nM) on *jimpy* iPSC-derived OPCs after 3 days of oligodendrocyte differentiation. n=1 replicate well per compound dose. Values indicate the number of SDs above the negative control (DMSO vehicle control) mean for MBP+ oligodendrocytes. Values that met the primary screening hit selection criterion for the MBP+ cell metric are highlighted in green. All other values highlighted in red. NR2B-NMDA receptor-selective antagonists highlighted in grey.

**Supplemental Experimental Procedures**

Mice

All animal work was sanctioned by Case Western Reserve University's Institutional Animal Care and Use Committee (IACUC). Wild-type (B6CBACa/J $A^{w-J}$/A; 001201, Jackson Laboratory) and *jimpy* mutant (B6CBACa-$A^{w-J}/A$-$Plp1^{jp}$ $Eda^{Ta}$/J; 000287, Jackson Laboratory) mice were housed and bred at Case Western Reserve University in a temperature-controlled environment with a 12-hour light / 12-hour dark cycle. Mice were provided a standard diet and water, *ad libitum*. Genotyping was performed by a quantitative PCR assay (Transnetyx) or Sanger sequencing using the forward and reserve primers AACGCAAAGCAGCACATTTCA and AGTGCAGCTCTGGGGTTAAT, respectively.

Immunochemistry and staining

Samples were fixed in 4% paraformaldehyde (PFA; 15710, Electron Microscopy Sciences), permeabilized, and blocked with 10% normal donkey serum (NDS; 017-000-121, Jackson Labs). The following primary antibodies used: rabbit anti-GFAP (1:5000; Z033429-2, Dako), rat anti-GFAP (1:1000; 13-0300, Thermo Fisher), mouse anti-MBP (1:500; 808401, Biolegend), rat anti-MBP (1:100; ab7349, Abcam), rabbit anti-MyRF antibody (1:1000; kindly provided by Dr. Michael Wegner), rabbit anti-Nanog (1:500; AB21624, Abcam), mouse anti-O4 (1:10; Lerner Research Institute Hybridoma Core), mouse anti-Oct3/4 (1:500; SC-5279, Santa Cruz), rabbit anti-Olig2 (1:1000; 13999-1-AP, ProteinTech), mouse anti-O4 (1:10; Lerner Research Institute Hybridoma Core), rat anti-PLP (1:500; Lerner Research Institute Hybridoma Core), goat anti-Sox10 (1:100; AF2864, R&D Systems), and rat anti-PLP (1:500; Lerner Research Institute Hybridoma Core). For secondary immunostaining Alex Fluor antibodies (ThermoFisher) were used at 1:500. To identify nuclei cells were stained with 100ng/ml DAPI (D8417, Sigma).

Tissue and cell culture

All cell and tissue cultures were maintained at 37° C with 5% CO2 in a humidified chamber. All OPC, oligodendrocyte, and astrocyte cultures were maintained on plates with coated with 100 µg/mL poly(L-ornithine) (P3655, Sigma), followed by 10 µg/ml laminin (L2020, Sigma). Plates were also purchased with a poly(D-lysine) which substituted for poly(L-ornithine).

Imaging and quantification of *in vitro* cultures

Unless otherwise noted immunochemistry images were captured using the Operetta® High Content Imaging and Analysis system (PerkinElmer) and Harmony® software (PerkinElmer). Images were analyzed and quantified using Columbus™ software (PerkinElmer) and a custom Acapella® (PerkinElmer) script. All phase images were captured on a Leica DM IL LED inverted microscope.

Isolation of tail-tip fibroblasts

Tail tips of approximately two millimeters were obtained from postnatal day nine *jimpy* mutant mice (B6CBACa-$A^{w-J}/A$-$Plp1^{jp}$ $Eda^{Ta}$/J; Jackson Laboratory) as well as wild-type (B6CBACa-$A^{w-J}$; Jackson Laboratory) littermates. Tissue was bisected, placed on Nunclon-Δ 12-well plates (150628, ThermoFisher), and covered with a circular glass coverslip (12-545-102; Fisher Scientific) to maintain tissue contact with the plate and enable fibroblast outgrowth. Tail-tip fibroblasts were cultured in 'fibroblast medium' consisting of DMEM (11960069, ThermoFisher) with 10% fetal bovine serum (FBS; 16000044, ThermoFisher), 1x non-essential amino acids (11140050, ThermoFisher), 1x Glutamax (35050061, ThermoFisher), and 0.1 mM 2-mercaptoethanol (M3148, Sigma Aldrich) supplemented with 100U/mL penicillin-streptomycin (15070-063, ThermoFisher). Medium was changed every third day.

TTF reprograming and iPSC generation

TTFs were lifted with Accutase (AT104, Innovative Cell Technologies) and were seeded at 1.3-2.0x$10^4$ cells/$cm^2$ on Nunclon-Δ dishes in fibroblast medium and were allowed to equilibrate overnight. The following day medium was removed and replaced with an equal volume of pHAGE2-TetOminiCMV-STEMCCA-W-loxp (Somers et al., 2010; Sommer et al., 2009) lentivirus encoding a floxed, doxycycline-inducible polycistronic Oct4, Sox2, Klf4, and c-Myc construct (Takahashi et al., 2007; Welstead et al., 2008) and pLVX-Tet-On-Puro (632162, Clontech) lentivirus supplemented with 8µg/mL polybrene (107689, Sigma). Lentivirus was prepared using the *Lenti*-X Packaging Single *Shots (631275, Clontech) according to manufacturer's instructions*. Three hours later lentivirus medium was removed and replaced with fibroblast medium supplemented with 2 µg/ml doxycycline (631311, Clontech). The following day media was removed and replaced with an equal volume of pHAGE2-TetOminiCMV-STEMCCA-W-loxp and pLVX-Tet-On-Puro lentivirus supplemented with 8µg/mL polybrene. Three hours later lentivirus media was

removed and replaced with fibroblast medium supplemented with 2 μg/ml doxycycline and $10^3$ units/ml LIF. After four days medium was changed with fibroblast medium supplemented with 2 μg/ml doxycycline and $10^3$ units/ml LIF. After expansion tail-tip fibroblasts were lifted using Accutase and seeded at 6.25-12.5x$10^4$ cells/$cm^2$ on Nunclon-Δ plates, atop a feeder layer of irradiated mouse embryonic fibroblasts (iMEFs; produced in-house) previously plated at 3.3x$10^4$ cells/$cm^2$ on 0.1% gelatin (1890, Sigma) coated Nunclon-Δ plates in "pluripotency medium" consisting of Knockout DMEM (10829-018, ThermoFisher), 5% FBS, 15% knockout replacement serum (10828028, ThermoFisher), 1x Glutamax, 1x nonessential amino acids, 0.1 mM 2-mercaptoethanol, and $10^3$ units/ml LIF (LIF; ESG1107, EMD Millipore). Medium was changed every day until iPSC colonies began to emerge. Individual colonies were picked and dissociated in 0.25% Trypsin-EDTA (25200056, ThermoFisher) and were individually plated in single wells of Nunclon-Δ 12-well plates, atop an iMEF feeder layer. iPSCs were expanded in pluripotency medium supplemented with doxycycline and medium was changed every day. Three individual iPSC clones per genotype were expanded and cryopreserved for future use (wild-type line identifiers = i.wt-1.0, i.wt-1.3, and i.wt-1.4 and *jimpy* lines identifiers = i.jp-1.4, i.jp-1.5, and i.jp-1.6). G-banded chromosomal analysis was performed on all iPSC lines at the seventh passage after derivation (Cell Line Genetics; Madison, WI). Approximately twenty cells were chosen per line and chromosomes were analyzed in metaphase spreads. Wild-type or *jimpy* genotypes were verified by Sanger sequencing.

<u>OPC derivation from iPSCs</u>

iPSCs were differentiated to OPCs as previously described (Najm et al., 2011, Lager et al., 2018). In brief, iPSC were isolated from their iMEF feeder layer using 1.5mg/mL collagenase type IV (17104019, ThermoFisher) and dissociated with either 0.25% Typsin-EDTA or Accutase and seeded at 7.8x$10^4$ cells/$cm^2$ on Costar Ultra-Low attachment 6-well plates (3471, Corning). Cultures were then directed through a 9-day differentiation process to generate "unenriched OPCs". Unenriched OPCs were further purified over three passages using OPC-selective medium. OPCs were maintained in "OPC medium" consisting of DMEM/F12 (11320082, ThermoFisher), 1x N2 supplement (AR009, R&D Systems), 1x B-27 without vitamin A supplement (12587-010, ThermoFisher), and 1x Glutamax (collectively "N2B27 medium"), supplemented with 20 ng/mL fibroblast growth factor 2 (FGF2; 233-FB, R&D Systems) and 20 ng/mL platelet-derived growth factor-AA (PDGF-AA; 221-AA, R&D Systems). Medium was changed every other day. The expected genotype for each wild-type or *jimpy* OPC line was confirmed by Sanger sequencing.

<u>Characterization and purity assessment of iPSC-derived OPCs</u>

For characterization of OPCs from all wild-type and *jimpy* iPSC lines, OPCs were seeded at 2.6 x$10^5$ cells/$cm^2$ in quadruplicate wells on Nunclon-Δ plates. Actively growing OPC cultures were fixed with 4% PFA and OPCs were immunostained using goat anti-Sox10, and rabbit anti-Olig2, followed by counterstaining with DAPI. 20 fields were captured per well and the total number of DAPI+, Sox10+, and Olig2+ cells were used to quantify the percentage of Olig2 and Sox10 double or single positive OPCs for each cell line.

<u>Oligodendrocyte assessment after oligodendrocyte differentiation</u>

Cultures of all wild-type and *jimpy* iPSC-derived OPCs (n=3 independent cell lines per genotype) were harvested in parallel using Accutase and seeded at 30,000 cells per well (n=4 wells per cell line) on 96-well Nunclon-Δ plates in "oligodendrocyte differentiation medium" which consisted of N2B27 medium supplemented with 100ng/mL noggin (3344-NG, R&D Systems), 10ng/mL neurotrophin-3 (NT-3; 267-N3, R&D Systems), 50 μM cAMP (D0260, Sigma), 100ng/mL insulin-like growth factor-1 (IGF-1; 291-G1, R&D Systems) NT-3, and 40ng/mL triiodothyronine (thyroid hormone; T-6397, Sigma). At 3 day post-plating medium was removed and cells were fixed with 4% PFA. Cells were immunostained using rat anti-MBP, followed by counterstaining with DAPI. 8 fields were captured per well and the total number of MBP+ cells were quantified for each cell line. A two-way, unpaired t-test was performed using Graphpad Prism software to compare statistical differences between genotypes.

<u>Temporal dynamics of OPC differentiation to oligodendrocytes</u>

Cultures of wild-type and *jimpy* iPSC-derived OPCs (from iPSC lines i.wt-1.0 and i.jp-1.6, respectively) were harvested in parallel using Accutase and seeded at 30,000 cells per well in 96-well poly-D-lysine Viewplates (6005710, PerkinElmer) in oligodendrocyte differentiation medium (n=6 replicate wells per genotype). At 2hr (plating control), 1 day, 2 day, and 3 day post-plating medium was removed and cells were live stained with mouse anti-O4 followed by fixation with 4% PFA. Cells were then immunostained using rat anti-MBP, followed by counterstaining with DAPI. 10 fields were captured per well and the total number of MBP+ cells were quantified for each cell line. To control for differences in cell plating, cell counts were normalized to the 2hr plating control.

Live-cell imaging of OPC differentiation
Parallel cultures of wild-type and *jimpy* iPSC-derived OPCs (from iPSC lines i.wt-1.0 and i.jp-1.6, respectively) were harvested in parallel using Accutase and seeded at $3.6x10^4$ cells/cm$^2$ in oligodendrocyte differentiation medium on a Nunclon-Δ plate. This plate was then positioned in an environmental chamber with temperature, humidity, and $CO_2$ control, and regions of interest were marked manually using Leica Application Suite X software. Phase images were automatically captured every 15 minutes during 3 days of oligodendrocyte differentiation using a DMI6000 microscope (Leica). A single image series for each genotype was stitched together to generate a movie using Leica Application Suite X software, and each movie was processed for background correction and aligned using Image Pro Premier 9 software (Media Cybernetics). Wild-type and *jimpy* time-lapse movies were placed side-by-side in iMovie (version 10.1.5).

RNA-seq and Gene Ontology Enrichment Analysis
Parallel cultures of OPCs from wild-type and *jimpy* iPSC lines (n=3 independent cells lines, per genotype) were seeded at $2.3x10^4$ cells/cm$^2$ in oligodendrocyte differentiation or OPC medium on Nunclon-Δ flasks. One day later cells were homogenized in TRIzol (15596026, ThermoFisher). Samples were chloroform extracted and phase separated. RNA was isolated using the miRNeasy Mini kit (217004, Qiagen). Sequencing libraries were generated using the TruSeq Stranded Total RNA kit (20020596, Illumina) after rRNA depletion with RiboZero Gold (MRZG126, Illumina). 100bp paired-end sequencing was performed on an Illumina HiSeq 2500 at the Case Western Reserve University School of Medicine Genomics Core Facility. Reads were aligned to the iGenomes mm9 genome using default settings in Tophat v2.0.6 (Trapnell et al., 2009), without providing a reference transcriptome. Abundances of transcripts in the iGenomes mm9 RefSeq transcriptome were calculated using Cufflinks v2.0.2 (Trapnell et al., 2010) to generate data normalized FPKM values.

Expressed genes were used for gene ontology analysis. All biological replicates for each condition were averaged and genes with a FPKM >0.25 in at least one condition were considered expressed. Expressed genes were tabled by converting FPKM values <0.25 to 0.25. Day one oligodendrocyte differentiation conditions were normalized to their respective OPC condition. Normalized expression values were then compared between genotypes: [(*jimpy* D1 oligodendrocyte/*jimpy* OPC)]/[(wild-type D1 oligodendrocyte/wild-type OPC)]. Normalized, differential expression values were evaluated using Gene Set Enrichment Analysis software (Subramanian et al., 2005), generating gene set enrichment scores using Hallmark, C2.all, and C5.all Molecular Signatures Database (MSigDB) datasets. Gene sets with an FDR of <0.05 were processed with Cytoscape v3.2.1 (Shannon et al., 2003) and the Enrichment Map plug-in (Merico et al., 2010) to generate pathway networks. *Plp1* expression in OPCs and day 1 oligodendrocyte differentiation samples were assessed using data normalized FPKM values. Each day 1 oligodendrocyte differentiation sample was further normalized by dividing by the FPKM value of its respective OPC line.

scRNA-seq by Drop-Seq and GSEA
Wild-type and *jimpy* iPSC-derived OPCs (from iPSC lines i.wt-1.0 and i.jp-1.6, respectively) were harvested in parallel using Accutase and seeded at $6.6x10^4$ cells/cm$^2$ in oligodendrocyte differentiation medium on Nunclon-Δ flasks. One day later cells were harvested using Accutase and resuspended in N2B27 containing 0.1% bovine serum albumin (15260037, ThermoFisher) and immediately processed for Drop-Seq as previously described (Macosko et al., 2015). In brief, three individual syringes containing cell suspension at 200,000 cells/mL barcoded mRNA capture beads (MACOSKO-2011-10, ChemGenes) in lysis buffer (prepared according to instructions in Drop-Seq protocol (Macosko et al., 2015) at 200,000 beads/mL, and QX200 droplet generation oil (186-4006, Bio-Rad), respectively, were placed in a Legato™ 100 pump (788100, Analytical West). Each output was connected to a custom-made PDMS co-flow microfluidic droplet generation device (Nanoshift) under microscope supervision. Cell and bead flow was set to 4,000uL/hr, and oil flow was set to 15,000uL/hr during droplet generation. These settings were optimized to allow at most one bead per one cell. Droplets were collected and then broken to release beads. First strand cDNA synthesis with incorporation of a PCR handle was performed on the collected beads using the Maxima H Minus Reverse Transcriptase (EP0752, ThermoFisher). The resulting full-length cDNA library was prepared for sequencing as described (Macosko et al., 2015) and sequenced on an Illumina HiSeq 2500 at the Case Western Reserve University School of Medicine Genomics Core Facility. Raw reads were processed as described (Macosko et al., 2015). The resulting reads were aligned to iGenomes mm9 RefSeq transcriptome using STAR v.2.5 (Dobin et al., 2013) with default settings. Mapped reads were filtered to contain only uniquely mapped, exonic reads. Reads were then grouped by cell barcode, generating a digital UMI-count matrix quantifying the number of unique transcripts per gene. We confirmed that wild-type and *jimpy* cells had similar profiles of sequenced transcripts per cell (Supplementary Figure 3a).

To perform unsupervised clustering we first identified 500 genes with high inter-cell expression variability. To identify this gene set we employed cells with at least 1000 total transcripts. We summed all transcripts per gene and ordered genes by expression level (highest to lowest). The top 10,000 genes were divided into 10 bins by expression level, with each bin containing 1,000 genes. Coefficient of variation (CV) was calculated within each bin for each gene. We selected the 50 genes per bin with the highest variability and combined them into a final list of 500 highly-variable genes.

Basic clustering was executed using the Seurat package (Macosko et al., 2015; Satija et al., 2015) using default filtering parameters. Next we performed principle component analysis (PCA) PCA using our 500 highly-variable gene list (see above). With PC dimension from PC1 to PC10, cells were embedded in a K-nearest neighbor (KNN) graph. Smart local moving algorithm (SLM) was applied for grouping cells into communities. tSNE was performed with PC1 to PC10 as input and cell clusters were viewed in tSNE space.

To define cell identity, we used the Seurat *FindMarker* function to identify genes with high expression and cluster exclusivity. Expression of specific markers (*Top2a* and *Ccnd1* for OPCs, *Apoe* and *Slc1a2* for nascent astrocytes, *Mbp* and *Enpp6* for nascent oligodendrocytes) was used to apply cell-type classifications to the different clusters. For differential expression analysis we extracted the expression matrix for wild-type and *jimpy* OPCs, nascent oligodendrocytes, and nascent astrocytes.

We employed a generalized linear model using the R package *MASS* by command ***glm.nb*** as shown below:

$$\log(\mathrm{UMI}) = \beta_0 + \beta_1 C + \beta_2 D + \log(sf)$$

where **log(UMI)** is assumed to have a negative binomial distribution

**UMI** represents the number of transcripts
$\beta_0$ is the regression intercept
$\beta_1$ is the parameter for C
**C** represents the cell type, a factor data type in R to describe the cell type for a given cell
**D** represents the donor, a factor data type in R to describe the origin of a given cell
**sf** is the size factor. This is calculated by command ***computeSumFactors*** in Bioconductor package *scran* sf to describe the size of a given cell, as previously described (Lun et al., 2016)

The response variable is UMI, which is regressed on two predictors: C; D. The key factor is C, which models the variance between cell types for a given gene. D and sf are used to regress out the influence of different individual and the difference in cell origin and cell library size, respectively. With regards to the estimation for sf, since the dropout is zero in single cell data, we made use of deconvolution strategy to normalize and calculate cell library size using the command ***computeSumFactors*** in Bioconductor package ***scran*** **sf** as previously described (Lun et al., 2016). We modeled all genes across all cell types iteratively and used the Wald test to calculate per gene p-values. We adjusted each p-value with the Bioconductor package ***qvalue*** to generate estimated q-values.

Per gene fold-change differences between wild-type and *jimpy* cell types (OPCs, nascent oligodendrocytes, and nascent astrocytes) were calculated in two ways: 1) Mean fold change and 2) fold change as a percentage of expression. We filtered genes using the q-value (<0.05) and ranked them by the fold change. For enrichment analysis we selected genes with a p value <0.01 and fold change >1.2 or <0.8 to capture the top up or down regulated genes, respectively. Finally we performed GSEA using MSigDB datasets to show pathway enrichment in *jimpy* nascent oligodendrocytes, OPCs, or nascent astrocytes relative to wild-type nascent oligodendrocytes, OPCs, or nascent astrocytes, respectively.

<u>Astrocyte differentiation and co-culture</u>
Wild-type and *jimpy* iPSC-derived OPCs (from iPSC lines i.wt-1.0 and i.jp-1.6, respectively) were harvested in parallel using Accutase and seeded at 10,000 OPCs per well in two 96-well Nunclon-Δ plates in 'astrocyte medium' which consisted of 1:1 neurobasal media (211030-049, ThermoFisher) and DMEM (11960-044, ThermoFisher) with 1mM sodium pyruvate (ThermoFisher Cat#: 11360-070), 1x Glutamax, 1x N2 supplement, and 30uM N-acetyl-cysteine (A8199, Sigma) supplemented with 10ng/ml of CNTF (557-NT, R&D Systems) and 50ng/ml of BMP4 (314-BP, R&D Systems) (Mabie et al., 1999; Scholze et al., 2014). Three days later one astrocyte differentiation plate

(astrocyte characterization plate) was fixed with 4% PFA and immunostained using rabbit anti-GFAP followed by counterstaining with DAPI (n=18 replicate wells per genotype). Medium was changed to N2B27 medium on the remaining astrocyte differentiation plate (astrocyte co-culture plate). After three days separate cultures of wild-type and *jimpy* iPSC-derived OPCs (from iPSC lines i.wt-1.0 and i.jp-1.6, respectively) were harvested in parallel using Accutase and seeded at 30,000 OPCs per well in oligodendrocyte differentiation medium on *jimpy* or wild-type iPSC-derived astrocytes, respectively (astrocyte co-culture plate), or on a 96-well Nunclon-Δ plate (oligodendrocyte differentiation control plate). Three days later astrocyte co-culture and oligodendrocyte differentiation control plates were fixed with 4% PFA. The oligodendrocyte differentiation control (n=8 replicate wells per genotype) and astrocyte co-culture (n=6 replicate wells per genotype) plates were immunostained using mouse anti-MBP followed by counterstaining with DAPI. 9 fields were captured per well and the total number of DAPI+ and GFAP+ cells were used to quantify the percentage of GFAP+ cells for the astrocyte characterization plate. The total number of DAPI+ and MBP+ cells were used to quantify the number of oligodendrocytes for the oligodendrocyte differentiation control and astrocyte co-culture plates.

Compound screening and assessment

384-well poly-D-lysine CellCarrier Ultra plates (6057500, PerkinElmer) were coated with 10 μg/ml laminin solution in N2B27 using a Biotek EL406 Microplate Washer Dispenser with a 5μL dispenser cassette (7170011, Biotek) and incubated at 37°C. Bioactive compound libraries dissolved in DMSO were accessed from Case Western Reserve University's Small Molecule Drug Development Core. Compounds were added to the CellCarrier Ultra plates with a 50nL pin head coupled to a Perkin Elmer JANUS® automation workstation. The following compounds were used for compound screening and assessment: Salubrinal (SML0951, Sigma), Q-VD-OPh (OPH001, R&D systems), emricasan (HY-10396, MedChem Express), rislenemdaz (HY-106441A, MedChem Express), TCN 237 (4072, Tocris Bioscience), TCS 46b (2782, Tocris Bioscience). For the >3000 bioactive compound primary screen or compound cherry-picking, two separate compound libraries were used (L1700, Selleck Chemicals and LO1280, Sigma). Additionally DMSO vehicle negative control and 10μM Q-VD-OPh (R&D Systems) plus 10μM salubrinal (Sigma) positive control were added to column 23 and column 24 of each primary screen plate. For cell plating, OPCs were harvested using Accutase and seeded at 15,000 cells per well using the Biotek EL406 Microplate Washer Dispenser with a 5μL dispenser cassette. All assays were performed using i.jp-1.6 or i.wt-1.0 OPCs unless otherwise indicated. Final medium consisted of oligodendrocyte differentiation medium and appropriate concentration of compound. After three days plates were fixed and immunostained with rat anti-MBP and goat anti-Sox10, and counterstained with DAPI using a Biotek EL406 Washer Dispenser outfitted with a 96-well aspiration manifold and a 5μL dispenser cassette. Plates were imaged on the Operetta® High Content Imaging and Analysis system (PerkinElmer). 5 fields were captured and the total number of cells and MBP+ oligodendrocytes were quantified using a robust Acapella® (PerkinElmer) script (Figure S5d-h). All staining, imaging, and analysis steps were performed simultaneously for each screen using identical procedures to reduce plate-to-plate variability.

Isolation and expansion of primary *jimpy* OPCs

Postnatal day two *jimpy* pups were cyro-anesthetized and euthanized by rapid decapitation and whole brains were isolated in ice cold DMEM/F12 with 50U penicillin-streptomycin. Cerebral cortex was dissected from surrounding tissue and meninges were removed. Tissue was transferred to tissue dissociation solution (from kit 130-095-929, Miltenyi Biotec) and incubated at 37° C for 10 minutes with gentle trituration every 5 minutes. Cell solution was filtered through a 70μm Corning Falcon™ cell strainer (08-771-2, ThermoFisher) and cells were seeded at 2.6-7.1 x $10^4$ cells/$cm^2$ in OPC medium supplemented with 100U/mL penicillin-streptomycin, on Nunclon-Δ plate. Medium was changed every other day. At 80-95% confluence cells were passaged 1:2 with Accutase for one passage in OPC selection medium, followed by one additional passage in OPC medium. Medium was changed every other day. Primary cortical cultures were used immediately after passage 2.

NR2B-NMDA receptor assessment by qRT-PCR

Parallel cultures of OPCs from wild-type and *jimpy* iPSC lines (i.wt-1.0 and i.jp-1.6, respectively) were simultaneously harvested using Accutase and seeded at $5.2x10^4$ with oligodendrocyte differentiation medium on Nunclon-Δ plates. Cultures were harvested at days 0, 1, 2, and 3 using TRIzol reagent. Positive control samples consisting of cerebral cortex from age-matched postnatal week 3 wild-type and *jimpy* mice were collected using TRIzol reagent. All samples were chloroform extraction and phase separated in parallel. RNA was prepared with the RNeasy Mini kit (74104, Qiagen) according to the manufacturer's instructions. cDNA was generated using 1μg of total RNA per reaction using the iScript cDNA synthesis kit (1708890, Bio-Rad) according to the manufacturer's instructions. Quantitative RT-PCR was performed using 2.5ng RNA per reaction and the following pre-designed Taqman gene expression assays

(ThermoFisher): *Grin2b* (Mm00433820_m1) and *Actb* (Mm00607939_s1). *Actb* was used as an endogenous control to normalize expression values. n=3 technical replicates for the i.wt-1.0 day 0 sample and n=4 technical replicates for all other samples were analyzed on the Applied Biosystems 7300 Real-time PCR system. Samples were normalized to the wild-type cerebral cortical positive control.

Ro 25-6981 mechanistic studies by qRT-PCR

Parallel cultures of OPCs from wild-type and *jimpy* iPSC lines (i.wt-1.0 and i.jp-1.6, respectively) were simultaneously harvested using Accutase and seeded at $7.8 \times 10^4$ cells/$cm^2$ in oligodendrocyte differentiation or OPC medium containing DMSO vehicle on Nunclon-Δ plates. Additionally wild-type and *jimpy* OPCs were plated in oligodendrocyte differentiation medium containing 5μM Ro 25-6981 (R7150, Sigma) or 10μM salubrinal (Sigma). One day later RNA was harvested from all conditions using TRIzol reagent followed by chloroform extraction and phase separation. RNA and cDNA were prepared as described above. Quantitative RT-PCR was performed using 10ng RNA per reaction the following pre-designed Taqman gene expression assays (ThermoFisher): *Plp1* (Mm01297210_m1), *Ddit3* (Mm01135937_g1), *Atf4* (Mm00515325_g1), *Dnajb9* (Mm01622956_s1), *s-Xbp1* (Mm03464497_s1), *Hspa5* (Mm00517691_m1), and *Atf6* (Mm01295319_m1). *Actb* was used as an endogenous control to normalize expression values. n=3 technical replicates per *Atf6* sample and n=4 technical replicates for all other samples were analyzed on the Applied Biosystems 7300 Real-time PCR system. To assess for differentiation-specific expression changes all samples in oligodendrocyte differentiation medium were normalized to their respective OPC control lines.

Treatment of Human PMD Oligocortical Spheroids with Ro 25-6981

Human PMD cortical spheroids were generated using PMD $PLP1^{c.254T>G}$ and gene-corrected (isogenic control) iPSCs according to an established cortical spheroid protocol (Pasca et al., 2015) with modifications described in (Madhavan et al., 2018). From days 60-90 medium was supplemented with DMSO vehicle or 1 μM Ro 25-6981. Ro 25-6981 dose was chosen based its *in vitro* efficacy in *jimpy* cultures while also minimizing the possibility of potential toxicities from long-term culturing with the compound. At day 90 four spheroids (cultured in independent wells) per treatment were collected, fixed in 4% PFA for 12 minutes, cryoprotected in 30% sucrose, embedded in Tissue-Tek® Optimum Cutting Temperature compound (O.C.T.; 25608-930, VWR) and cryosectioned to produce 10 μm sections. Sections were immunostained using rat anti-PLP and rabbit anti-MyRF antibody, and counterstained with DAPI. Images were captured using a Leica SP8 Gated STED Microscope or a Leica DMi8 inverted microscope (for cell counts). Image analysis and cell counting was accomplished using Adobe Photoshop (Abobe Systems). The total number of DAPI+ and MyRF+ cells from equally sized and spaced fields per spheroid were counted and n=4 spheroids for each treatment condition were used to calculate the percentage of MyRF+ oligodendrocytes in each treatment group.

Treatment of postnatal *jimpy* pups with Ro 25-6981

From the onset of corpus callosal myelination at postnatal day 5 to day 14 jimpy pups were administered 1 mg/kg or 10mg/kg Ro 25-6981 (1594, R&D Systems), or saline vehicle control by intraperitoneal (i.p.) injection once a day. As an additional control wild-type male littermates were administered saline by i.p. injection once a day. Experimenters were blinded to the treatment being administered. At day 14 all injected mice were deeply anesthetized using isoflurane and transcardially perfused with PBS followed by fixation with 4% PFA. Tissue was cyroprotected with 30% sucrose, embedded in O.C.T. and cryosectioned at 20 μm thickness. Sections were immunostained using rabbit anti-MyRF and rat anti-MBP antibody. Images of the complete corpus callosum (medial, sagittal sections) were captured using a Leica SP8 Gated STED Microscope or a Leica DMi8 inverted microscope (for cell counts). Image analysis and cell counting of MyRF+ cells across the entire corpus callosum was accomplished using Adobe Photoshop, n=4 independent animals per treatment. One-way ANOVA with Holm-Sidak correction for multiple comparisons was performed using Graphpad Prism software to compare statistical differences between treatments.

Myelination assessment of Ro 25-6981 using synthetic microfibers

Mimetix® 384-well plates containing aligned 4 μm diameter poly-lactide fibers labeled with rhodamine-6G (AMS-TECL-015, AMSBio) were incubated in 70% ethanol. Next fibers were coated with 100 μg/mL poly(L-ornithine) followed by 10 μg/ml laminin. Wild-type and *jimpy* iPSC-derived OPCs (i.wt-1.0 and i.jp-1.6, respectively) were harvested using Accutase and, after removal of laminin solution, seeded at 15,000 OPCs per well in oligodendrocyte differentiation medium using a Biotek EL406 Microplate Washer Dispenser with a 5μL dispenser cassette (Biotek) and incubated at 37°C. Ro 25-6981 (R&D systems), as well as the additional modulators of *jimpy* pathology including salubrinal (Sigma) and Q-VD-OPh (R&D Systems), were all dissolved in DMSO. Compounds were added to the microfiber plates with a 50nL pin head coupled to a Perkin Elmer JANUS® automation workstation to achieve the

appropriate final concentration in each well. From days 0-3 medium was supplemented with 40ng/mL thyroid hormone to induce oligodendrocyte differentiation. On day 3 thyroid hormone was removed, and medium was subsequently changed every third day. Compounds or DMSO vehicle were added at each medium change. At day 3 and day 10 plates were fixed. All plates were immunostained with rat anti-MBP and counterstained with DAPI using a Biotek EL406 Washer Dispenser outfitted with a 96-well aspiration manifold and a 5μL dispenser cassette. Plates were imaged on the Operetta® High Content Imaging and Analysis system. A total of five fields were captured at 20x using Acapella® software, and images were analyzed using Harmony® software and Columbus™ software. Using this analysis software we developed an Acapella® script to quantify the total fiber area surrounded by MBP+ oligodendrocytes (Figure S7a-e). First, rhodamine signal was detected, quantified, and used as a mask in subsequent steps (Figure S7b,d). Next MBP signal was detected and a threshold was set to reduce false positive calls (Figure S7c,e). Finally the percent MBP+ coverage relative to total microfiber area was calculated. All staining, imaging, and analysis steps were performed simultaneously for each microfiber plate using identical procedures to reduce plate-to-plate variability.

Data Availability

All RNA-seq data have been deposited to the Gene Expression Omnibus (GEO) database under the accession number GSE111605.

**Supplemental References**

Dobin, A., Davis, C.A., Schlesinger, F., Drenkow, J., Zaleski, C., Jha, S., Batut, P., Chaisson, M., and Gingeras, T.R. (2013). STAR: ultrafast universal RNA-seq aligner. Bioinformatics *29*, 15-21.

Lun, A.T., Bach, K., and Marioni, J.C. (2016). Pooling across cells to normalize single-cell RNA sequencing data with many zero counts. Genome Biol *17*, 75.

Mabie, P.C., Mehler, M.F., and Kessler, J.A. (1999). Multiple roles of bone morphogenetic protein signaling in the regulation of cortical cell number and phenotype. The Journal of neuroscience : the official journal of the Society for Neuroscience *19*, 7077-7088.

Macosko, E.Z., Basu, A., Satija, R., Nemesh, J., Shekhar, K., Goldman, M., Tirosh, I., Bialas, A.R., Kamitaki, N., Martersteck, E.M.*, et al.* (2015). Highly Parallel Genome-wide Expression Profiling of Individual Cells Using Nanoliter Droplets. Cell *161*, 1202-1214.

Merico, D., Isserlin, R., Stueker, O., Emili, A., and Bader, G.D. (2010). Enrichment map: a network-based method for gene-set enrichment visualization and interpretation. PLoS One *5*, e13984.

Pasca, A.M., Sloan, S.A., Clarke, L.E., Tian, Y., Makinson, C.D., Huber, N., Kim, C.H., Park, J.Y., O'Rourke, N.A., Nguyen, K.D.*, et al.* (2015). Functional cortical neurons and astrocytes from human pluripotent stem cells in 3D culture. Nature methods *12*, 671-678.

Satija, R., Farrell, J.A., Gennert, D., Schier, A.F., and Regev, A. (2015). Spatial reconstruction of single-cell gene expression data. Nature biotechnology *33*, 495-502.

Scholze, A.R., Foo, L.C., Mulinyawe, S., and Barres, B.A. (2014). BMP signaling in astrocytes downregulates EGFR to modulate survival and maturation. PLoS One *9*, e110668.

Shannon, P., Markiel, A., Ozier, O., Baliga, N.S., Wang, J.T., Ramage, D., Amin, N., Schwikowski, B., and Ideker, T. (2003). Cytoscape: a software environment for integrated models of biomolecular interaction networks. Genome Res *13*, 2498-2504.

Subramanian, A., Tamayo, P., Mootha, V.K., Mukherjee, S., Ebert, B.L., Gillette, M.A., Paulovich, A., Pomeroy, S.L., Golub, T.R., Lander, E.S.*, et al.* (2005). Gene set enrichment analysis: a knowledge-based approach for interpreting genome-wide expression profiles. Proceedings of the National Academy of Sciences of the United States of America *102*, 15545-15550.

Takahashi, K., Tanabe, K., Ohnuki, M., Narita, M., Ichisaka, T., Tomoda, K., and Yamanaka, S. (2007). Induction of pluripotent stem cells from adult human fibroblasts by defined factors. Cell *131*, 861-872.

Trapnell, C., Pachter, L., and Salzberg, S.L. (2009). TopHat: discovering splice junctions with RNA-Seq. Bioinformatics *25*, 1105-1111.

Trapnell, C., Williams, B.A., Pertea, G., Mortazavi, A., Kwan, G., van Baren, M.J., Salzberg, S.L., Wold, B.J., and Pachter, L. (2010). Transcript assembly and quantification by RNA-Seq reveals unannotated transcripts and isoform switching during cell differentiation. Nature biotechnology *28*, 511-515.

Welstead, G.G., Brambrink, T., and Jaenisch, R. (2008). Generating iPS cells from MEFS through forced expression of Sox-2, Oct-4, c-Myc, and Klf4. J Vis Exp.
